# Hematopoietic stem cells retain functional potential and molecular identity in hibernation cultures

**DOI:** 10.1016/j.stemcr.2021.04.002

**Published:** 2021-05-06

**Authors:** Caroline A. Oedekoven, Miriam Belmonte, Daniel Bode, Fiona K. Hamey, Mairi S. Shepherd, James Lok Chi Che, Grace Boyd, Craig McDonald, Serena Belluschi, Evangelia Diamanti, Hugo P. Bastos, Katherine S. Bridge, Berthold Göttgens, Elisa Laurenti, David G. Kent

**Affiliations:** 1 Wellcome MRC Cambridge Stem Cell Institute, University of Cambridge, Hills Road, Cambridge CB2 0XY, UK; 2 Department of Haematology, University of Cambridge, Cambridge CB2 0XY, UK; 3 York Biomedical Research Institute, Department of Biology, University of York, York YO10 5DD, UK

**Keywords:** hematopoietic stem cells, quiescence, cell cycle, stem cell niche, single-cell RNA sequencing, single-cell assays, transplantation

## Abstract

Advances in the isolation and gene expression profiling of single hematopoietic stem cells (HSCs) have permitted in-depth resolution of their molecular program. However, long-term HSCs can only be isolated to near purity from adult mouse bone marrow, thereby precluding studies of their molecular program in different physiological states. Here, we describe a powerful 7-day HSC hibernation culture system that maintains HSCs as single cells in the absence of a physical niche. Single hibernating HSCs retain full functional potential compared with freshly isolated HSCs with respect to colony-forming capacity and transplantation into primary and secondary recipients. Comparison of hibernating HSC molecular profiles to their freshly isolated counterparts showed a striking degree of molecular similarity, further resolving the core molecular machinery of HSC self-renewal while also identifying key factors that are potentially dispensable for HSC function, including members of the AP1 complex (*Jun*, *Fos*, and *Ncor2*), *Sult1a1* and *Cish*. Finally, we provide evidence that hibernating mouse HSCs can be transduced without compromising their self-renewal activity and demonstrate the applicability of hibernation cultures to human HSCs.

## Highlights

• Fully functional single HSCs are maintained in the absence of a hematopoietic niche
• scRNA-seq identifies the molecular regulators of HSC hibernation and SCF activation
• Human HSC activity can be retained in hibernation cultures
• Single HSCs can be transduced with lentiviral constructs during hibernation

## Teaser

In this study, the groups of David Kent and Elisa Laurenti show that fully functional mouse and human hematopoietic stem cells (HSCs) can be kept in a non-dividing state outside of their native microenvironment. Single-cell transplantations and RNA sequencing demonstrate a high degree of similarity to freshly isolated HSCs, but also identify molecular networks involved in HSC resilience.

## Introduction

The blood-forming system is sustained by a rare subset of hematopoietic stem cells (HSCs) with the potential to differentiate into all mature blood cell types and to create equally potent daughter HSCs to maintain tissue homeostasis (Doulatov et al., 2012; Eaves, 2015; Ganuza et al., 2020; Laurenti and Göttgens, 2018). As the seed cells for the blood system, their clinical potential for cellular therapies is vast and the need to understand their molecular program in different physiological states is critical for their therapeutic application. Recently, cell culture conditions have been reported to produce large numbers of functional mouse and human HSCs (hHSCs) (Fares et al., 2017; Wilkinson et al., 2019) but, in all cases, the substantial majority of cells produced are non-HSCs (Bak et al., 2018; Gundry et al., 2016; Shepherd and Kent, 2019; Wagenblast et al., 2019).

In the absence of robust purification strategies for functional HSCs in culture, it becomes virtually impossible to study the molecular profile of HSCs removed from their *in vivo* microenvironment. Previous studies have highlighted the potential for retaining long-term HSC (LT-HSC) function in cultures with low amounts of proliferation in the absence of excessive cytokine-induced signaling (Kobayashi et al., 2019; Yamazaki et al, 2006, 2009), although these cultures were still predominantly non-HSCs. An *in vitro* system that could retain highly purified single HSCs would offer the potential to molecularly profile niche-independent HSCs and to resolve the essential components of self-renewal *in vitro*.

Here, we describe such a system, demonstrating that fully functional mouse LT-HSCs can be maintained in minimal cytokine conditions over a period of 7 days without undergoing cell division. This novel cell culture system preserves the core features of HSCs, including the speed of quiescence exit, subsequent cell-cycle kinetics, mature cell production, and HSC self-renewal activity in serial transplantation assays. The functional properties of these hibernating HSCs are virtually indistinguishable from freshly isolated HSCs and molecular profiling by single-cell RNA sequencing (scRNA-seq) shows a high degree of overlap with freshly isolated HSCs, but also reveals a number of molecular changes that identify genes potentially dispensable for retaining HSC function.

## Results

### Single LT-HSCs can retain multipotency *in vitro* under minimal cytokine stimulation

**Figure 1:**
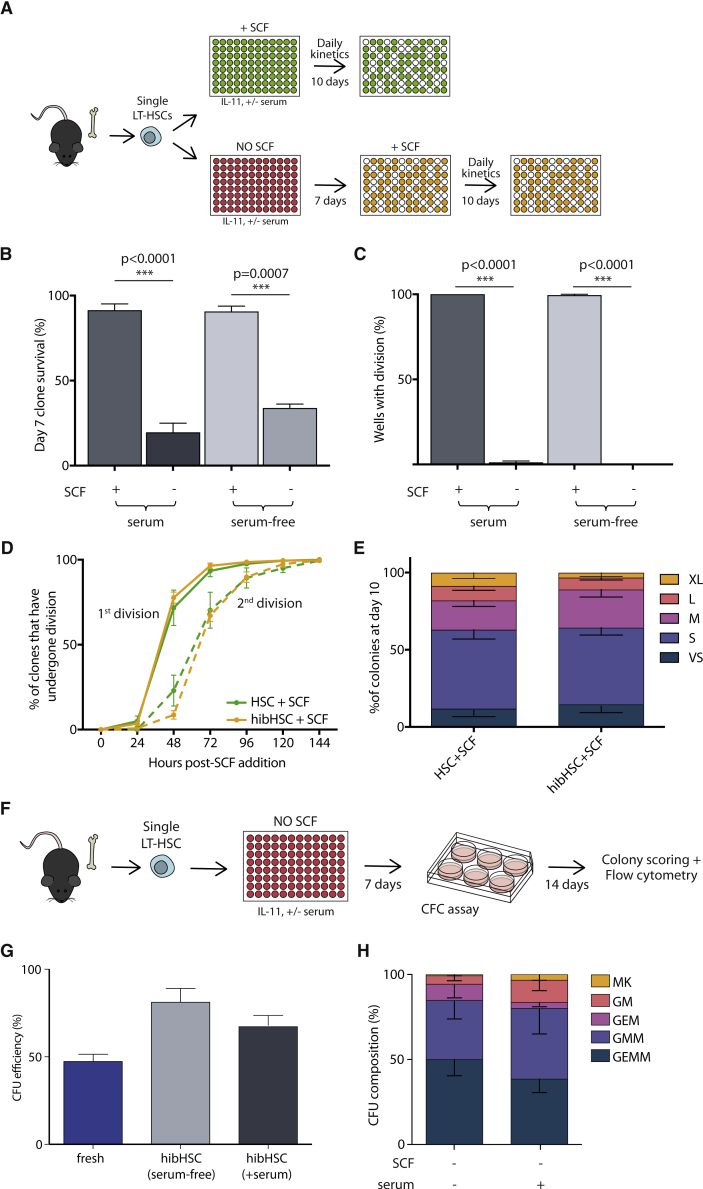
Absence of SCF and TPO maintains HSCs as single multi-potent cells *in vitro* (A) Single CD45^+^EPCR^+^CD48^−^CD150^+^Sca1^high^ LT-HSCs were sorted into individual wells and cultured in the presence of IL-11, in serum-supplemented or serum-free medium and in the presence or absence of SCF. For SCF-supplemented cultures (green plate), daily cell counts were performed for 10 days. For cultures only containing IL-11 (red plate), HSCs were supplied with SCF on day 7 post-isolation after which daily cell counts were performed for an additional 10 days. In all cases, clone size was assessed at day 10 post-SCF addition. (B) HSC survival is decreased in the absence of SCF compared with SCF-supplemented medium (+serum/+SCF n = 355, 5 biological replicates; +serum/−SCF n = 1,722, 7 independent experiments; −serum/+SCF, N = 144, 2 independent experiments, −serum/−SCF n = 284, 3 independent experiments). (C) Numbers of wells with >2 cells were scored to determine the number of clones that had divided. At day 7 post-isolation, only culture conditions without SCF maintained HSCs as single cells. (D) Cell division kinetics post-SCF addition. Entry into cell cycle was comparable between freshly isolated HSCs (green solid line) and cells that had been maintained as single cells for 7 days (orange solid line) in serum-supplemented media. Time to subsequent cell division (dotted lines) was not significantly different between conditions (SCF added at day 0, n = 355, 5 independent experiments; SCF added at day 7, n = 1,722, 7 independent experiments). (E) Colony size was measured on day 10 post-SCF addition and no difference in clone size distribution was observed between HSCs cultured in the presence of SCF from day 0 and post-hibernation HSCs (day 7 + 10). (F) Single LT-HSCs were cultured for 7 days in IL-11 alone, in serum-supplemented or serum-free medium. After 7 days, single hibernating LT-HSCs were individually transferred into a cytokine-rich methylcellulose CFC assay and cultured for an additional 14 days. On day 14, lineage composition of individual colonies was assessed by flow cytometry. (G) Colony-forming efficiency for freshly isolated single LT-HSCs, single LT-HSCs cultured in serum-supplemented and serum-free hibernating cultures (fresh, n = 300, 3 biological replicates; serum-free, n = 121, 5 independent experiments; +serum, n = 230, 6 independent experiments). (H) Colony subtype analysis showed that the majority of single cells (~80%) generated colonies of at least three lineages in colony-forming unit (CFU) assays (hibHSC serum-free, n = 70, 4 independent experiments; hibHSC + serum, n = 166, 3 independent experiments). Colonies were defined as MK (containing cells positive for megakaryocyte marker CD41), GM (containing cells positive for granulocyte/monocyte markers Gr1 and CD11b), GEM (positive for GM and erythrocyte markers Gr1, CD11b, and Ter-119), GMM (positive for GM and MK markers), and GEMM (positive for GM, MK, and E markers), as described in the Experimental procedures. Bars show mean with SEM. Unpaired t test: ^∗^p < 0.05, ^∗∗^p < 0.01, ^∗∗∗^p < 0.001.

Previous studies suggested that stem cell factor (SCF) and thrombopoietin (TPO) are essential for HSC self-renewal and proliferation, but potentially dispensable for stem cell maintenance (Yamazaki et al., 2006, 2009; De Graaf and Metcalf, 2011). A number of studies use gp130 family members (e.g., interleukin-11 [IL-11], IL-6) in HSC maintenance conditions, including our own studies which typically use 20 ng/mL of IL-11 alongside 300 ng/mL of SCF (Kent et al., 2008b; Kent et al., 2013; Shepherd et al., 2018). To test the absence of SCF and TPO, we cultured single mouse bone marrow CD45^+^EPCR^+^CD48^−^CD150^+^Sca1^high^ LT-HSCs, which are ∼60% functional HSCs by single-cell transplantation (Wilson et al., 2015), in the presence of 20 ng/mL IL-11 alone in both serum-containing (Kent et al., 2013; Shepherd et al., 2018) and serum-free conditions (Wilkinson et al., 2019) (Figure 1A). Between 20% and 40% of single LT-HSCs survived 7 days of culture (Figure 1B), making them considerably more resilient to cytokine depletion than single sorted progenitor cell fractions (Lin^−^Sca1^+^c-Kit^+^), where no cells survived past 2 days (data not shown). Interestingly, 99.2% (634 of 639 cells) of the surviving input LT-HSCs were maintained as single cells for the 7-day period (Figure 1C), and single-cell time-lapse imaging and tracking confirmed that cells did not undergo division followed by death of one daughter cell (Video S1). Together this prompted us to term the minimal cytokine condition as a “hibernation” condition, similar to the cellular state of LT-HSCs described after the addition of lipid raft inhibitors (Yamazaki et al., 2006).

Video S1. Single-cell time-lapse imaging of single HSCs in hibernation cultures

To assess the functional potential of single LT-HSCs in the hibernation condition (hibHSCs), 300 ng/mL SCF was added to mirror cytokine combinations previously applied to freshly isolated LT-HSCs (Kent et al., 2008a; Kent et al., 2013; Shepherd et al., 2018). Time to first and second division was indistinguishable from freshly isolated LT-HSCs receiving SCF (Figure 1D), and clonal proliferation and survival over the subsequent 10 days was also similar, as indicated by clone size distribution being nearly identical to freshly isolated HSCs stimulated for 10 days (Figure 1E). In accordance with this, single hibHSCs also retained their multipotency in colony-forming cell (CFC) assays (Figures 1F and 1G) and 60%–70% of single cells generated at least three different lineages (Figure 1H), as determined by flow cytometry. Together, these data suggest that HSCs surviving cytokine depletion exist in a state of prolonged hibernation and can be revived to function indistinguishably from freshly isolated HSCs.

### Hibernating HSCs are fully functional in transplantation assays

**Figure 2:**
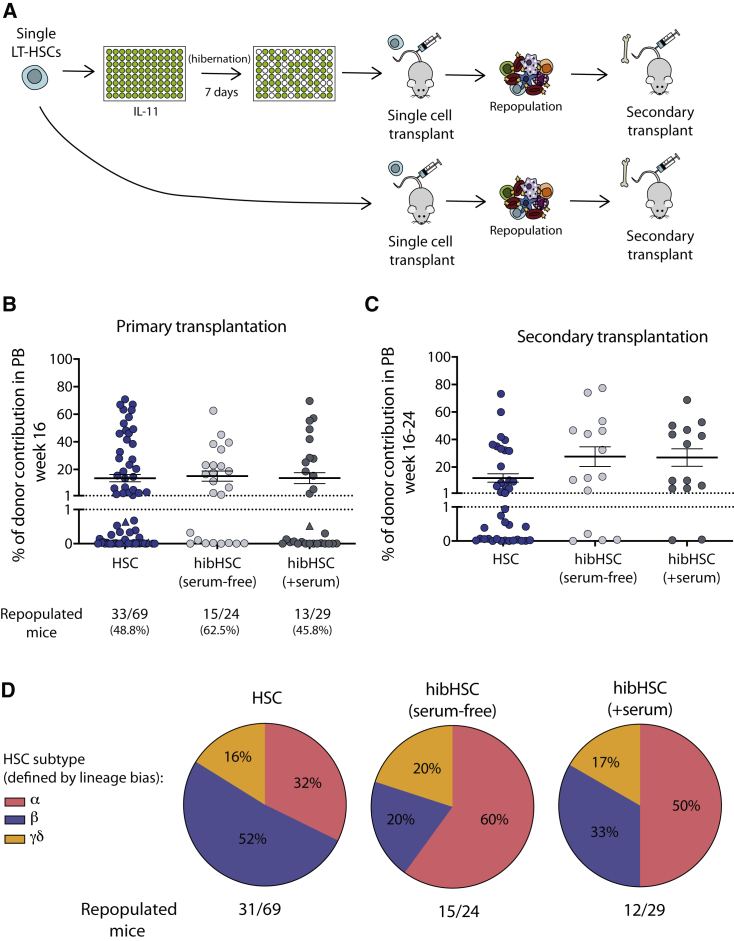
Hibernating HSCs maintain *in vivo* functional activity (A) HSCs were cultured in hibernation conditions in either serum-supplemented or serum-free medium. Single fresh or day 7 hibernating LT-HSCs were transplanted into W41-CD45.1 recipients (fresh n = 69; serum-free n = 24; +serum n = 29). Secondary transplantations were undertaken in all mice with donor engraftment (>1%) at 16–24 weeks post-transplantation. (B and C) Graphs show percent donor chimerism in the peripheral blood of primary (B) and secondary (C) recipient mice at 16–24 weeks post-transplantation. Recipients with chimerism >1% and at least 0.5% of GM, B, and T cells were considered to be repopulated. (Triangles represent mice where chimerism reached >1% at weeks 20–24 post-transplantation but had not done so by 16 weeks.) (D) No significant difference was observed in the balance of mature cell outputs between freshly isolated and post-hibernation HSCs. Based on donor myeloid (M) to lymphoid (L) ratio at 16 weeks in primary recipients, the founder HSC was retrospectively assigned one of the following subtypes: α (alpha, M:L > 2), β (beta, M:L > 0.25 < 2), γ (gamma, M:L < 0.25), δ (delta, M:L < 0.25 and failure to contribute to myeloid lineage past 16 weeks) in accordance with Dykstra et al. (2007) (HSC n = 31/69; hibHSC (+serum) n = 12/29; hibHSC (serum-free) n = 15/24).

To assess whether cells cultured in the absence of SCF or TPO retained their HSC self-renewal expansion capability, single day 7 hibHSCs were transplanted and their repopulation capacity was compared with freshly isolated HSCs (Figure 2A); 62.5% (15/24) and 45.8% (13/29) of primary recipients transplanted with single hibHSCs (without serum and with serum, respectively) had >1% multi-lineage donor chimerism at 16–24 weeks post-transplantation compared with 48.8% (33/69) of freshly isolated HSCs (Figure 2B). Secondary transplantation efficiency was also high (Figure 2C), suggesting that the period of 7 days *in vitro* had no impact on HSC self-renewal. This was further supported by the observation of no significant differences in mature cell production between hibHSCs and freshly isolated HSCs, as determined by the relative proportions of the HSC subtype produced in single-cell transplantation experiments (Figure 2D). Notably, despite these high functional purities, the total yield of functional HSCs was slightly lower considering that some HSCs do not survive hibernation. These data provide formal evidence that, following 7 days of SCF and TPO depletion and in the complete absence of a supportive stem cell niche, LT-HSCs can retain full functional potential as assessed by serial transplantation.

### High CD150 expression prospectively enriches for resilient HSCs

**Figure 3:**
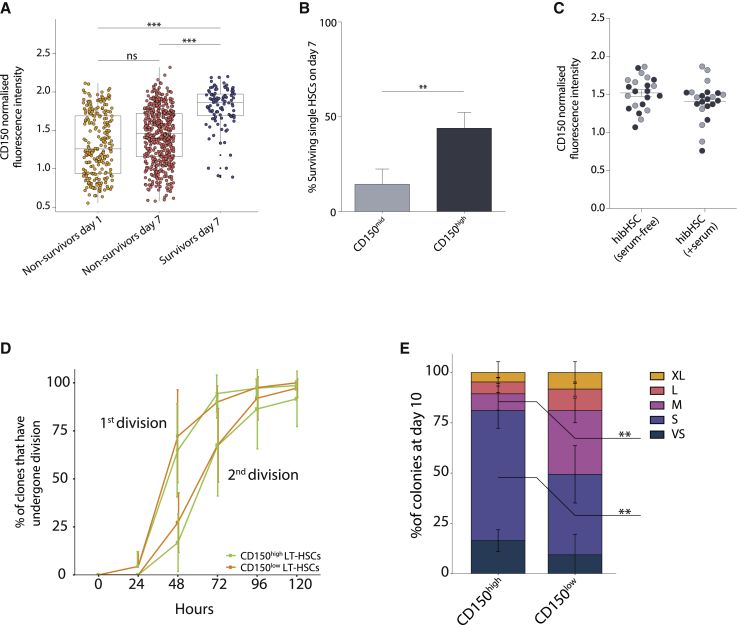
Higher expression of CD150 identifies resilient LT-HSCs (A) Flow cytometric index-sort data were used to determine the CD150 expression level of LT-HSCs at the time of isolation. Cells that did not survive at day 1 and day 7 were compared with those that survived out to day 7, with the latter population of cells correlating with higher CD150 expression. A boxplot shows the median with interquartile range. Vertical lines represent outermost quartiles. Black dots, if present, are extreme outliers. Unpaired t test: ^∗^p < 0.05, ^∗∗^p < 0.01, ^∗∗∗^p < 0.001. (B) Prospectively sorted CD150^high^ LT-HSCs show 4.2-fold higher survival than CD150^mid^ LT-HSCs (n = 480, 5 independent experiments). Paired two-tailed t test. (C) Hibernating HSCs in serum-free and serum-supplemented conditions were transplanted, and their CD150 levels retrospectively assessed. Cells able to repopulate a recipient (black) did not differ in initial CD150 expression levels compared with cells unable to repopulate (gray). (D) HSCs with high or low expression of CD150 were determined using index-sorting data from freshly isolated HSCs that were cultured for 7 days in serum-free medium supplemented with 20 ng/mL IL-11 and 300 ng/mL SCF. Three biological replicates were analyzed, and in each case the top third and bottom third of CD150 expressers were analyzed as CD150^high^ and CD150^low^, respectively. Daily cell counts were performed to assess cell division kinetics. Entry into cell cycle and the second division were not significantly altered between CD150^high^ and CD150^low^ LT-HSCs. (E) Using the same experimental data from Figure 3D, colony sizes from single LT-HSCs were measured on day 10 and clone sizes from single LT-HSCs with high expression of CD150 were significantly reduced compared with those with low CD150 expression (bars show mean with SD. Sidak's multiple comparison test: ^∗∗^p < 0.01).

Since only a proportion of phenotypic LT-HSCs survive hibernation conditions, we used flow cytometric index-sort data to determine whether levels of specific cell surface markers might associate with survival. Expression levels of the SCF receptor (c-Kit) did not select for surviving HSCs, while higher CD45 and EPCR expression were modestly increased on hibHSCs compared with cells that did not survive hibernation conditions (data not shown). High CD150 expression strongly associated with higher survival at day 7 (Figure 3A). To verify whether CD150 could be used to prospectively enrich for resilient HSCs, single LT-HSCs were sorted as CD150^mid^ or CD150^high^ and cultured in hibernation conditions. CD150^high^ HSCs show significantly higher (4.2-fold) survival on day 7 compared with CD150^mid^ HSCs, confirming that CD150 expression can enrich for phenotypic LT-HSCs that could survive hibernation conditions (Figure 3B). We next assessed whether CD150 levels on surviving LT-HSCs associated with successful transplantation and found no significant differences in CD150 intensity between HSCs that successfully repopulated recipients versus those that did not (Figure 3C). Interestingly, when we compared the cell division kinetics and 10-day colony size of single HSCs with high versus low expression of CD150, we observed smaller colonies from cells expressing high levels of CD150 (Figures 3D and 3E). Together these data suggest that, while higher CD150 expression can isolate cells enriched for resilient LT-HSCs with lower *in vitro* proliferation, the cells with lower CD150 expression that survive do not have compromised transplantation ability, which is supported by previous datasets examining CD150 expression in freshly isolated and transplanted HSCs (Beerman et al., 2010; Morita et al., 2010; Wilson et al., 2015).

### Hibernating LT-HSCs can be transduced without undergoing division

**Figure 4:**
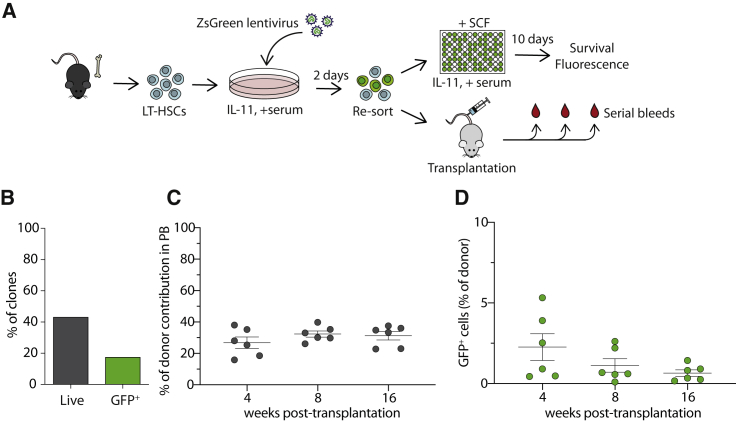
Single hibernating HSCs can be manipulated by lentiviral transduction (A) CD45^+^EPCR^+^CD48^−^CD150^+^ (ESLAM) cells were isolated and transduced with ZsGreen lentivirus and cultured together for 2 days in StemSpan supplemented with 10% fetal calf serum and IL-11. Cells were collected and virus was removed by collecting and re-sorting the cells into single wells and cultured in SCF-supplemented media for additional 10 days. A total of 4,001 total viable cells (a mixture of transduced and non-transduced cells) were re-sorted and transplanted into W41-CD45.1 (n = 6 recipients), and donor contribution and GFP expression were assessed by serial bleeds and flow cytometry analysis. (B) Graph shows the percentage of clones surviving after 10 days post-addition of SCF, and the green bar indicated the percentage of GFP^+^ clones. (C and D) Chimerism levels (20%–40%) were stable across all recipients at all time points (C), and an average of 1%–2% of donor cells was positive for GFP at 16 weeks post-transplantation (D). Bars show mean with SEM.

To further explore the experimental and clinical potential of hibHSC culture conditions, we next assessed whether transgenes could be delivered during the hibernation period. Small bulk populations of LT-HSCs were isolated and exposed to a GFP-containing lentivirus for 2 days and then re-sorted into single-cell cultures to determine single-cell transduction efficiencies and survival (Figure 4A). After 10 days, 40% of the original sorted clones (284/657) successfully produced colonies, with ∼17.6% (50/284) of the surviving clones being GFP^+^ (Figure 4B). In a second experiment to assess the *in vivo* functional potential of transduced hibernating LT-HSCs, bulk cells were transplanted following the 2-day transduction and assessed for GFP^+^ donor cell repopulation at 4, 8, and 16 weeks post-transplantation (Figure 4A). All recipient mice were positive with initial reconstitution levels ranging from 2% to 6% GFP^+^ cells (Figures 4C and 4D), and this contribution was stable throughout the monitoring period, although early time points appear slightly higher, suggesting that HSCs with less-durable self-renewal might be preferentially transduced. Together, these data demonstrate that lentiviral constructs can be successfully delivered to LT-HSCs in hibernation cultures without cell division.

### Hibernating LT-HSCs share a core gene expression program with freshly isolated LT-HSCs

LT-HSCs deprived of SCF and TPO in hibernation conditions retain their functional properties, including the ability to reconstitute primary and secondary recipients (Figures 2B and 2C). Aside from IL-11, these LT-HSCs were cultured without signals from the hematopoietic niche or neighboring cells, making the transcriptome of these LT-HSCs a useful comparator for determining which genes might be dispensable for LT-HSC function. To address this question, we performed scRNA-seq on LT-HSCs cultured in serum-free hibernating conditions for 7 days (n = 106) and compared them to freshly isolated single LT-HSCs (n = 165) and also to LT-HSCs stimulated with SCF for 16 h (from both HSC + SCF [n = 63] and hibHSC + SCF [n = 127]) to determine the common pathways of activation upon SCF stimulation.

**Figure 5:**
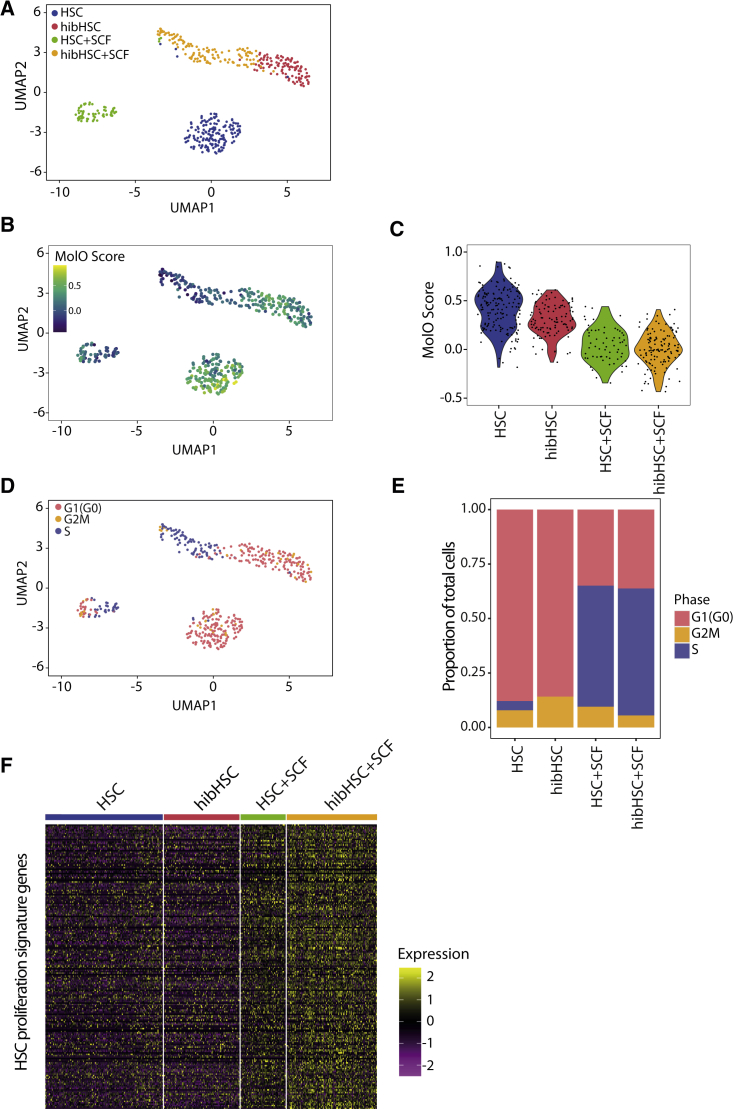
Gene expression profiling reveals a common transcriptional program between freshly isolated and hibernating HSCs (A) Uniform Manifold Approximation and Projection (UMAP) of scRNA-seq profiles derived from four distinct populations (HSC, blue dots; hibHSC, red dots; HSC + SCF, green dots; hibHSC + SCF, orange dots). (B) The HSC-specific Molecular Overlap (MolO) gene signature score was computed based on average expression of signature genes and projected onto the UMAP distribution. (C) MolO scores for the individual HSCs in each physiological state with the HSCs and hibHSCs having the highest overall scores. (D) Cell-cycle scores were computed for each cell and identified states were projected on the UMAP display from 5A (G1(G0), pink; G2/M, orange; S, blue). (E) A proportional representation of cell-cycle stages of all cells within each distinct population (G1(G0), pink; G2/M, orange; S, blue). (F) Heatmap of previously identified HSC-specific proliferation signature genes (Venezia et al., 2004) sorted by cell type with low expression in HSCs and hibHSCs and high expression in both sets of SCF-stimulated cells.

To determine broad differences between cell fractions, we performed dimensionality reduction using Uniform Manifold Approximation and Projection (UMAP) on single cells from all four conditions. Cells from each physiological setting clustered together in a unique space (Figures 5A and S1A). These data indicate that, while there is substantial similarity to the molecular profile of freshly isolated HSCs, there are some molecular changes that result from being removed from the *in vivo* microenvironment for 7 days.

To assess the similarity of hibHSCs to freshly isolated HSCs further, we compared the expression levels of key HSC regulators that comprise the previously reported Molecular Overlap (MolO) gene signature (Wilson et al., 2015). Overlaying MolO scores on the UMAP plot shows that the highest MolO scores are present in the freshly isolated HSCs, followed by the hibHSCs, and then their SCF-stimulated counterparts (Figure 5B). This pattern is mirrored in the violin plots displaying individual single-cell MolO scores by physiological condition (Figure 5C). Individual genes comprising the MolO score and their relative expression across the four biological states are provided in Figure S1B. The relatively high MolO scores in hibHSCs indicates the utility of the MolO score for identifying functional HSCs irrespective of their physiological state. The similarity in these molecular features also suggests that other factors must be contributing to the clear separation between freshly isolated HSCs and hibHSCs.

Another example of molecular similarity between hibHSCs and freshly isolated HSCs was evident when components of the cell-cycle machinery were assessed to predict the cell-cycle stage of each profiled LT-HSC (Nestorowa et al., 2016; Hamey and Göttgens, 2019). Again, UMAP clustering (Figure 5D) shows that cell-cycle status is not the primary driver of molecular differences between freshly isolated and hibHSCs, with the vast majority of cells in both cases being in the G_0_/G_1_ phase of the cell cycle (Figure S1C). Overall, more than 80% of freshly isolated HSCs and hibHSCs had molecular profiles consistent with being in the G_0_/G_1_ phase of the cell cycle (Figure 5E), whereas both SCF-stimulated HSC fractions had fewer than 40% G_0_/G_1_ cells. These data also accord with the cell-cycle kinetics observed in Figure 1D, where cells that divide early in the curve (i.e., between 20 and 30 h after stimulation) would be expected to have progressed to the S or G_2_ phase by 16 h after stimulation. This is further emphasized by the heatmap in Figure 5E which displays the HSC proliferation gene signature from Venezia et al. (2004), where both freshly isolated and hibHSCs express low levels of proliferation-related genes (Figures 5F and S2A–S2E). Finally, we also assessed markers of autophagy and senescence and in neither case did we observe a significant enrichment (Figures S3A and S3B).

### Hibernation cultures resolve common pathways of cytokine activation

**Figure 6:**
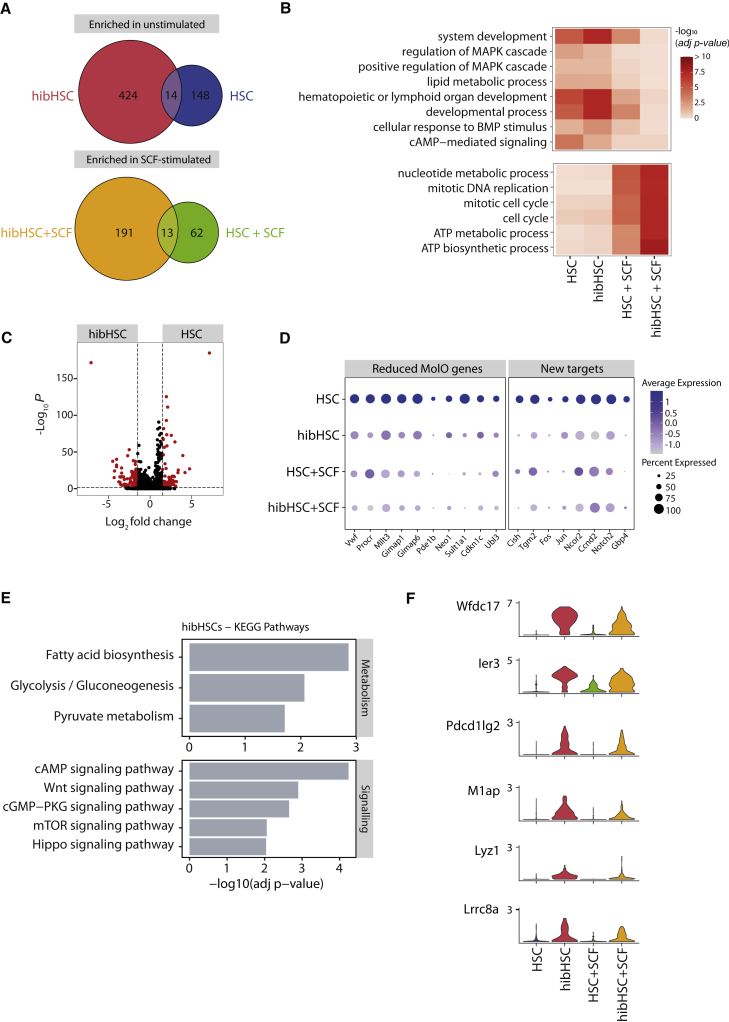
Hibernating HSCs have a unique molecular profile of stress response (A) Differential gene expression (DGE) was computed for two separate comparisons: (1) comparison of fresh HSCs (HSC) against SCF-stimulated HSCs (HSC + SCF); (2) comparison of hibernating HSC (hibHSCs) against hibHSCs post-SCF-stimulation (hibHSC + SCF) (negative binomial distribution, adjusted with Benjamini-Hochberg correction). Venn diagrams represent the number of genes commonly enriched in unstimulated populations (HSC and hibHSC) and SCF-stimulated populations (HSC + SCF and hibHSC + SCF) from both separate DGE computations. (B) Gene ontology term enrichment was computed based on differentially expressed genes, as outlined in (A). Minimum p value > 0.05 to be considered significantly enriched. (C) Volcano plot of differentially expressed genes (red dots), comparing fresh HSCs (HSC) and hibernating HSCs (hibHSC) (negative binomial distribution, adjusted with Benjamini-Hochberg correction). (D) Dot plot representing the average normalized expression of genes across the four distinct populations. Genes of interest and MolO signature genes were selected from DGE in (C). The size of each dot indicates the proportion of cells with normalized expression level >0 (scaled expression represented by color intensity). (E) KEGG pathway enrichment in unstimulated hibernating HSCs (hibHSC), showing selected metabolic and signal transduction pathways (enrichment cutoff: adjusted p value > 0.05). (F) Violin plots of normalized gene expression of selected differentially expressed genes, enriched in unstimulated hibernating HSCs (hibHSCs).

Historically, the molecular impact of adding specific cytokines to HSCs has been performed following their direct isolation from the *in vivo* microenvironment. However, the impact that membrane dynamics, protein turnover, and transcriptional priming would have on the response of an HSC to a particular extracellular signal remains unclear. Hibernation cultures offer a different physiological state of highly purified HSCs from which to understand the direct impact of cytokine addition to a functional HSC. First, we observed the impact of culturing HSCs in IL-11 alone during the hibernation condition, allowing us to resolve the pathways activated or suppressed in response to IL-11 (Figures S3C–S3E). Next, using SCF as a stimulant, we profiled freshly isolated HSCs and hibHSCs to identify individual gene expression patterns associated with SCF stimulation (HSC + SCF, hibHSC + SCF). We first generated differentially expressed gene lists from the HSC versus HSC + SCF and hibHSC versus hibHSC + SCF (Figure 6A). Twenty-seven genes were commonly differentially expressed (13 up and 14 down) upon addition of SCF with an expected activation of ATP generation and nucleotide metabolism alongside a number of positive cell-cycle mediators (*Mcm2*, *Mcm4*, *Mcm10*, *Rad51*, and *Rad51ap1*) and a reduction in developmental and MAPK-mediated signaling (Figures 6B and S4A). In addition to these expected changes, we also identified SCF targets specifically induced in HSCs (Figure S4A) and show that expression of Mif (Ohta et al., 2012) (an inflammatory cytokine promoting survival and proliferation) and Txn1 (Schenk et al., 1994) (regulator of AP-1 signaling) are directly promoted upon SCF addition to functional HSCs.

### Hibernating HSCs downregulate the AP1 complex and other stem cell regulators

Despite the strong overlap in cell-cycle and MolO gene signature expression, hibHSCs form a distinct cluster away from freshly isolated HSCs (Figures 5A and Figure S4B). While some of this distance could be attributable to downregulation of specific MolO genes (including *Sult1a1* and *Gimap1*, Figure 6D), global differential gene expression analysis between HSCs and hibHSCs identified 116 upregulated and 138 downregulated genes (Figure 6C). Among those additional genes whose expression was significantly reduced, a number of AP-1 complex members were identified, including *Jun* and *Fos* and their co-regulator *Ncor2* as well as molecules with previously described roles in HSC biology, such as *Cish* (Schepers et al., 2012) and *Vwf* (Figures 6D and S4C). Since hibHSCs retain their functional properties *in vivo*, these data suggest that high levels of these genes are not a requirement for HSC function. On the other hand, pathways that were highly upregulated in hibHSCs were associated with stress response and nutrient deprivation, consistent with being kept in minimal cytokine conditions, and KEGG pathway analysis identified cAMP and mTOR signaling (Dhawan and Laxman, 2015) alongside glycolysis and fatty acid biosynthesis (Figure 6E). This accords with enrichment of HSC pro-survival genes *Ier3* and *Pdcd1lg2* expression in hibernating HSCs. Of additional interest, multiple HLF target genes, including *Lyz1* and *Lrrc8a*, were overexpressed in hibernated HSCs, potentially supporting the notion that HSCs are exerting a stress response to maintain survival/quiescence (Komorowska et al., 2017) in response to cytokine deprivation (Figures 6F and S5).

### Human HSCs can be retained as single cells in hibernation conditions

**Figure 7:**
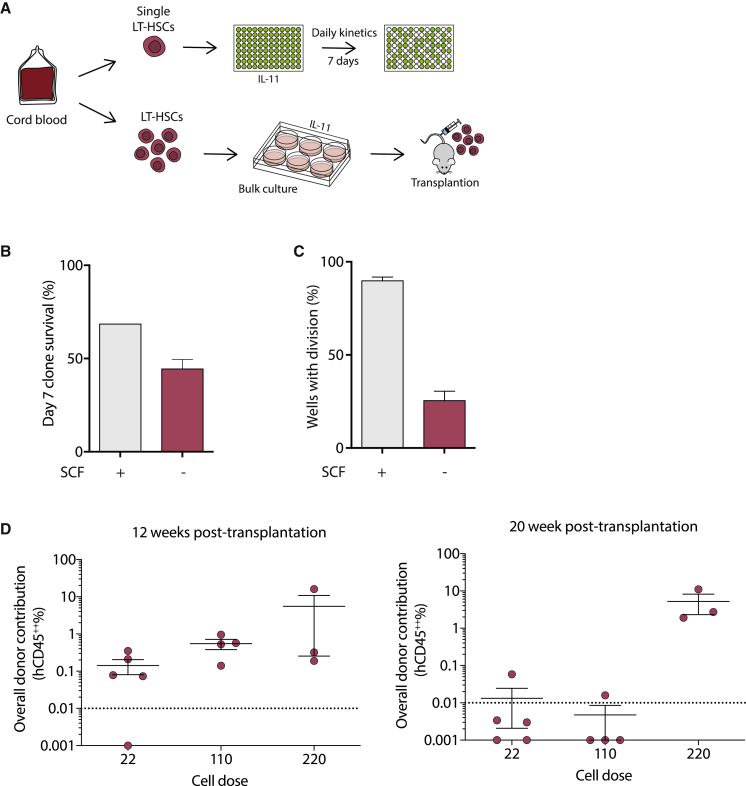
Hibernation conditions keep the majority of human HSCs as single cells (A) Single human HSCs (CD34^+^CD38^−^CD90^+^CD45RA^−^CD19^−^CD49f^+^) from umbilical cord blood were sorted into individual wells and cultured in the presence of IL-11 with or without SCF. In parallel, human HSCs were bulk-cultured for 7 days in the absence of SCF and transplanted at three different cell doses (22, 110, and 218) into immunodeficient recipients and monitored for engraftment. (B) Survival of HSCs in the presence or absence of SCF over 7 days, where absence results in 1.5-fold reduced survival compared with SCF-supplemented cultures (fresh n = 192; post-hibernation, n = 672; 5 independent experiments) (C) The proportion of cells divided at 5–7 days in culture with and without the addition of SCF is displayed. Significantly more cells divide in the presence of SCF with the majority of cells in hibernation conditions remaining as single cells (fresh, three independent experiments, post-hibernation, five independent experiments). Bars show mean with SEM. (D) The graphs show the percentage of human cell engraftment (%CD45^++^) in PB from transplanted mice at 12 and 20 weeks post-transplantation (cell dose 22, n = 5; 110, n = 4; 218, n = 3). The threshold for events considered as positive was >0.01% with a minimum of 30 analyzed events. Non-engrafted mice shown below the dashed line. CD45^++^ indicates cells positive for two distinct CD45 antibodies. Bars show mean with SEM.

To investigate whether cytokine deprivation had a similar effect on human HSCs (hHSCs), we isolated single human CD34^+^CD38^−^CD90^+^CD45RA^−^CD19^−^CD49f^+^ cells from cord blood and cultured them in serum-free medium with human IL-11 alone for 7 days (Figure 7A). Similar to mouse LT-HSCs, survival was lower with cytokine deprivation (Figure 7B) and, although some cells divided (∼25.6%, Figure 7C), a large proportion remained as single cells compared with hHSCs under standard cytokine conditions (Belluschi et al., 2018; Ortmann et al., 2015). The fact that some hHSCs divided may be due to the starting purity or activation state of HSCs from cord blood. Upon transplantation of limited numbers of day 7 cultured hHSCs, repopulation was stable out to 20 weeks post-transplantation, but donor repopulation was below detection for the lowest-dose recipients (Figure 7D). Together these results demonstrate that IL-11 alone can maintain a proportion of multi-potent hHSCs in a non-dividing state, but further culture optimization would be required to support retention of large numbers of fully functional hHSCs.

## Discussion

Recent studies have produced a substantial amount of single-cell gene expression data from normal and malignant hematopoietic cells isolated from the mouse bone marrow (Shepherd and Kent, 2019). As a result, the transcriptional program of a quiescent “steady-state” LT-HSC is firmly established. Which genes drive individual LT-HSC properties (e.g., quiescence, self-renewal, differentiation, stress response) is much less well understood, and is complicated by only being able to obtain highly purified functional LT-HSCs from a single physiological state (i.e., quiescent cells from the bone marrow niche). Indeed, studies that have compared LT-HSCs to their downstream progenitors have identified “cell-cycle” changes as the dominant molecular feature separating LT-HSCs from non-HSCs (Passegué et al., 2005; Wilson et al., 2015). Hibernation cultures allow us to isolate and maintain functional LT-HSCs for prolonged periods of time in the absence of other cells without undergoing cell division or differentiation, thereby allowing the resolution of the common molecular program of HSCs in different physiological state. We identify molecules potentially dispensable for HSC function and a common molecular program of SCF activation in purified HSCs from distinct states. Finally, our study also resolves a debate about the impact of serum exposure on the cell fate of LT-HSCs (Domen and Weissman, 2000; Ieyasu et al., 2017; Rogers et al., 2008), showing that LT-HSCs can be cultured in the presence of serum for 7 days without undergoing differentiation or proliferation.

Distinct endogenous signaling pathways have been shown to regulate LT-HSC survival, self-renewal, and proliferation in both mouse (Wohrer et al., 2014) and human (Knapp et al., 2017). A similar cellular phenomenon of hibernation was observed when LT-HSCs were exposed to inhibitors that blocked lipid raft clustering (even in the presence of SCF) and remained undifferentiated as single cells for 5–7 days in culture (Yamazaki et al., 2006). Despite being deprived completely of TPO and SCF signaling, our hibernation cultures contain IL-11, without which all cells die within 48 h. One of the key pathways activated by IL-11 is gp130, which has been historically implicated in a wide array of stem cell systems, including mouse embryonic stem cells with LIF (Nichols et al., 2001), the *Drosophila* germ stem cell niche with Upd (Amoyel and Bach, 2012), mouse neural stem cells with CTNF and LIF (Shimazaki et al., 2001), mouse muscle stem cells with OSM (Sampath et al., 2018), and mouse HSCs with IL-6 and IL-11 (Yoshida et al., 1996; Audet et al., 2001). Of particular interest, OSM was shown to promote muscle cell engraftment without inducing proliferation (Sampath et al., 2018), lending additional support to the hypothesis that gp130 stimulants may regulate survival of quiescent stem cells in multiple stem cell systems.

Whereas other *in vitro* conditions have been shown to maintain mouse LT-HSCs, these systems uniformly create populations of cells in which LT-HSCs are the vast minority of the final culture (Bak et al., 2018; Gundry et al., 2016; Wagenblast et al., 2019; Wilkinson et al., 2019). In the absence of a robust *in vitro* LT-HSC purification strategy, molecular studies are therefore compromised by large numbers of contaminating non-HSCs. Our study averts this issue by retaining functional LT-HSCs as single cells. The gene expression programs of single functional LT-HSCs in 7-day hibernation conditions show a high retention of known self-renewal regulators, and are consistent with the cells being in G_0_/G_1_. They also identify several regulators whose absence does not impact HSC engraftment or serially repopulation. One such set of factors was the AP1 complex, where expression of several members including *Jun*, *Fos*, and *Ncor2* was significantly reduced in hibernation cultures. This is potentially due to the hibernation cultures driving their extinguished expression and cells that do not have sufficient amounts of AP1 complex members do not survive. In contrast, i*n vivo* loss or reduced AP1 function leads to increased proliferation and differentiation (Santaguida et al., 2009). It may be that expression of these molecules is rescued upon transplantation when HSCs expand, although the SCF-induced entry into cell cycle does not on its own initiate their expression.

A previous study has reported that low cytokine concentration in culture facilitates the maintenance of engraftable mouse and hHSCs (Kobayashi et al., 2019) with reduced proliferation *in vitro* and this finding is supported by studies showing that slow-dividing LT-HSC clones were much more likely to retain HSC function (Dykstra et al., 2006; Laurenti et al., 2015). However, none of these studies were able to retain single LT-HSCs at high purities with indistinguishable properties from freshly isolated LT-HSCs, making it impossible to perform molecular studies on single functional HSCs or to manipulate them at the single-cell level. Hibernation cultures permit such analyses since single LT-HSCs do not lose any functional capacity with a highly similar, if not slightly improved, primary and secondary transplantation capacity compared with freshly isolated HSCs.

The finding that high CD150 expression levels prospectively identify resilient HSCs that survive hibernation are broadly consistent with data that implicate CD150 as a marker of LT-HSCs with more durable self-renewal capacity in serial transplantation assays (Beerman et al., 2010; Kent et al., 2009; Morita et al., 2010). The highest levels of CD150 also associated with a delayed engraftment in primary transplantations, an initial deficiency in making lymphoid cells (Kent et al., 2009; Morita et al., 2010), and an ability to create daughter HSCs with full multi-lineage potential (Dykstra et al., 2007; Komorowska et al., 2017). This further accords with the increased number of α-HSCs (myeloid-biased) observed in our transplantation data. The delay in engraftment observed generally in α-HSCs may be related to the dynamics of quiescence/activation of daughter LT-HSCs in a transplantation scenario and our *in vitro* hibernation system offers the chance to study HSC activation in a distinct physiological context with unprecedented resolution. This latter capacity is particularly important in the context of HSC transplantation where cells need to exit, and eventually return to, quiescence during any sort of *in vitro* culture period and subsequent re-seeding of recipient bone marrow.

Optimization of hibernation cultures for manipulating highly purified LT-HSCs would also have a wide range of applications in experimental and clinical research. The knowledge that LT-HSCs are fully functional during hibernation offers the opportunity to manipulate them at the single-cell level with precise assessment of the impact of specific modifications. Our data show that genetic modification can be undertaken in hibernation cultures which could potentially set the stage for the delivery of multiple viral constructs during the culture period. This would permit studies of combinatorial genetic modifications in highly purified LT-HSCs, as opposed to a heterogeneous pool of stem and progenitor cells typically assayed in such protocols. Finally, we provide proof-of-principle evidence that hibernation cultures can be adapted to the human setting, offering substantial potential for implementing genetic modifications in hHSCs and setting the stage for more precise interrogation of the functional properties of individual LT-HSCs.

## Experimental procedures

### Mice

C57BL/6-Ly5.2 (wild type) were purchased from Charles River (Saffron Walden, Essex, UK). C57BL/6w41/w41-Ly5.1 (W41) were bred and maintained at the University of Cambridge. Full details are available in the supplemental information.

### Isolation of mouse Sca1^high^ ESLAM HSCs, *in vitro* assays, and expression profiling

HSCs were isolated from the lineage-depleted cell suspension by using fluorescence-activated cell sorting using EPCR^high^, CD45^+^, Sca-1^high^, CD48^low/neg^, and CD150^+^ (or ESLAM), as described previously (Kent et al., 2009) with full details found in the supplemental information.

### Bone marrow transplantation assays and analysis

Donor cells were obtained from C56BL/6J mice (CD45.2). Recipient mice were C57Bl6W41/W41 (W41) mice as described previously (Benz et al., 2012; Kent et al., 2009).

Full details of transplantation and peripheral blood analysis are in the supplemental information.

### Lentiviral transduction of mouse HSCs

ESLAM HSCs (7,000 cells) were isolated and transduced with GFP-containing lentivirus; full details of the transduction method and assays are in the supplemental information.

### Isolation of human cord blood HSCs and *in vitro* assays

Cord blood samples were obtained from the Cambridge Blood and Stem Cell Biobank with informed consent from healthy donors in accordance with regulated procedures approved by the relevant Research and Ethics Committees. Details of HSC isolation and *in vitro* assays are given in the supplemental information.

### scRNA-seq

scRNA-seq analysis was performed as described previously in Picelli et al., 2014) (Smart-seq2), with full details given in the supplemental information. Data are publicly available using the GEO accession number: GSE160131. All code is available upon request.

### Xenotransplantation and analysis

Donor cells were obtained from CD34-enriched cord blood samples. Recipient mice were NSG. Full details of transplantation and peripheral blood analysis are given in the supplemental information.

## Author contributions

C.A.O., M.B., D.G.K., and E.L. conceived and designed the experiments. C.A.O., M.B., M.S.S., J.L.C.C., G.B., C.McD., and S.B. performed the experiments. C.A.O., M.B., D.B., F.K.H., E.D., and H.P.B. analyzed the data. M.B., D.B., and D.G.K. wrote the paper with input from E.L. and B.G.

## Declaration of interests

The authors declare no competing interests.

## Supplemental information

Document S1. Supplemental experimental procedures and Figures S1–S5

Table S1. JAK/STAT, MAPK, NKFB, PI3K/AKT gene sets JAK/STAT, MAPK, NFKB, PI3K/AKT gene sets manually curated to exclude ligand- and receptor-associated genes. See also Figure S1.

Document S2. Article plus supplemental information

## Footnotes

Supplemental information can be found online at https://doi.org/10.1016/j.stemcr.2021.04.002.
