# Supplementary material for: Hematopoietic stem cells retain functional potential and molecular identity in hibernation cultures
Source: Stem Cell Reports. 2021 May 6;16(6):1614–28. doi: 10.1016/j.stemcr.2021.04.002 (PMC8190576; doi:10.1016/j.stemcr.2021.04.002)
Supplement: Document S1. Supplemental experimental procedures and Figures S1–S5 [file mmc1.pdf]

Stem Cell Reports, Volume *16*

Supplemental Information

Hematopoietic stem cells retain functional potential and molecular identity in hibernation cultures

Caroline A. Oedekoven, Miriam Belmonte, Daniel Bode, Fiona K. Hamey, Mairi S. Shepherd, James Lok Chi Che, Grace Boyd, Craig McDonald, Serena Belluschi, Evangelia Diamanti, Hugo P. Bastos, Katherine S. Bridge, Berthold Göttgens, Elisa Laurenti, and David G. Kent

Supplementary Data Items

Mice

C57BL/6-Ly5.2 (WT) were purchased from Charles River Laboratory (Saffron Walden, Essex, UK). C57BL/6w41/w41-Ly5.1 (W41) were bred and maintained at the University of Cambridge. *NOD.Cg-Prkdc^{scid}Il2rg^{tm1Wjl}/SzJ* (NSG) mice were obtained from Charles River or bred in-house. Mice were maintained in the Central Biomedical Service (CBS) animal facility of Cambridge University and housed in specific pathogen-free environment, according to institutional guidelines.All the procedures performed were in compliance with the guidance on the operation of ASPA (Animals Scientific Procedures Act 1986), following ethical review by the University of Cambridge Animal Welfare and Ethical Review Body (AWERB).

Isolation of mouse Sca1^{high} ESLAM HSCs and *in vitro* assays

Bone marrow cells were isolated from spine, sternum, femora, tibiae and pelvic bones of both hind legs of WT mice. Bones were crushed in 2% Fetal Calf Serum (FCS, STEMCELL or Sigma Aldrich (Sigma)) and 1mM EDTA (Sigma) in PBS (Sigma). Red cell lysis was performed by treatment with Ammonium Chloride (NH_4Cl, STEMCELL). Depletion of mature lineage cells was performed using EasySep mouse hematopoietic progenitor cell enrichment kit (STEMCELL). HSCs were isolated from the lineage depleted cell suspension by using fluorescence-activated cell sorting (FACS) using EPCR^{high}, CD45^{+}, Sca-1^{high}, CD48$^{low/neg}$, CD150^{+} (or "ESLAM"), as described previously (Kent *et al.*, 2009), using CD45 FITC (Clone 30-F1,1 BD Biosciences, San Jose CA, USA (BD)), EPCR PE (Clone RMEPCR1560, STEMCELL), CD150 Pacific Blue (PB) or PE-Cy7 (Clone TC15-12F12.2, both from Biolegend, San Diego, USA (Biolegend)), CD48 APC (Clone HM48-1, Biolegend), Sca-1 Brilliant Violet (BV) 421 (Clone D7, Biolegend) and 7-Aminoactinomycin D (7AAD) (Life Technologies, Carlsbad, CA, USA (Life Technologies)). The cells were sorted in either purity or single sort mode on an Influx cell sorter (BD Biosciences, San Jose, CA, USA (BD)) using the following filter sets 488 530/40 (for FITC), 561 585/29 (for PE), 405 460/50 (for BV421), 640 670/30 (for APC), 561 750LP (for PE/Cy7), 640 750LP (for APC/Cy7), 405 520/35 (for BV510), 640 720/40 (for AF700), and 561 670/30 (for 7-AAD) or 405 450/50 (for DAPI). When single HSCs were required, the single-cell deposition unit of the sorter was used to place 1 cell into each well of a round bottom 96-well plate, each

well having been preloaded with 50uL medium which would be topped up with 50uL medium with 2X cytokines.

Normalisation of single cell index-sorting data

Surface marker intensity of single ESLAM HSCs across experiments were normalised and batch corrected by using the flowCore (version 1.42.3) and sva (version 3.24.4) R packages. HSCs were sorted in 96-well format and each plate was considered as an independent batch prior to batch correction. All recorded surface markers were arranged in a flow frame and subject to logicle transformation prior to batch correction. The analysis was computed in R (version 3.4.2) and performed by Daniel Bode. The original script was developed by Blanca Pijuan Sala.

Liquid cultures and clone size determination of mouse HSCs

Single HSCs were sorted and cultured into 100µL StemSpan SFEM (STEMCELL) supplemented with 300 ng/mL SCF (R&D Systems, Bio-Techne, Minneapolis, MI, USA, (R&D)), 20ng/mL human Interleukin-11 (IL-11, R&D), 2 mM L-Glutamine (Sigma), 1000 U/mL-100 µg/mL Penicillin-Streptomycin (Sigma), 100µM 2-Mercaptoethanol (Life Technologies). SCF concentration was 300ng/mL unless stated otherwise. 10% of FCS was supplemented when stated. For serum-free cultures, cells were sorted into Ham's F12 nutrient mixture (Gibco, ThermoFisher, Waltham, MA, USA (Gibco)) supplemented with 20 ng/mL human IL-11 (R&D), 300 ng/mL SCF (SCT or R&D), 2 mM L-Glutamine (Sigma), 1000U/mL-100 µg/mL Penicillin-Streptomycin (Sigma), 1% ITS-X (Insulin-Transferrin Selenium-Ethanolamine, Gibco), 100 mM HEPES (4-(2-hydroxyethyl)-1 piperazineethanesulfonic acid, Sigma), 100 mg/mL human serum albumin (HSA, Albumin Bioscience, Huntsville, AL, USA).

Cells were cultured at 37°C, 5% CO_2, 20% O_2. Cell counts were performed every 22-24 hours and cell cycle kinetics determined for the first and second division by visual inspection, scoring wells as having 1, 2, or 3-4 cells. Clone size at day 10 post-isolation was scored as very small (less than 50 cells), small (50-500 cells), medium (500-10,000 cells), or large (10,000 or more cells).

Time lapse of single mouse HSCs

Single cells were sorted into a 96 well plate and imaged on a Leica DMI3000 B microscope, housed inside an Okolab CO2 microscope cage incubator system. Custom written LabVIEW software was used to control a Prior Proscan III nanopositioning stage and acquire images via a Hamamatsu Orca Flash 4.0 camera. Cells were imaged every 50 minutes for the first 7 days, the fastest time resolution achievable with the system while allowing enough time for the autofocus routine to correctly execute at all 96 wells. On day 7, the plate was removed and 300ng/mL SCF was added to the 67 wells where there was a possibility of a viable cell, determined by eye. The reduction in well number allowed for an increase in time resolution to 35 minutes. By day 11, imaged well number was further reduced to 17 wells as it became more apparent in which wells cells were still viable. This allowed for a corresponding increase in time resolution to 20 minutes. Imaging continued until day 14.

Colony-forming assays of mouse HSCs

Single cultured cells (hibernated HSCs) were transferred from liquid culture into 600 µl of MethoCult GF M3434 (STEMCELL). Freshly isolated HSCs were isolated by FACS sorting (as described above) and plated into 3 mL Methocult GF M3434 (STEMCELL) and split across 2 wells of 6-well plates. Cells were cultured for 14 days and colony number was assessed by visual inspection and colony type scored by antibody staining with CD41 FITC (Clone MWReg30), CD61 PE (Clone 2C9.G2 (HMβ3-1), Ter119 PE-Cy7 (Clone TER-119), CD45.2 APC-Cy7 (Clone 104), Ly6G/Gr1 BV421 (Clone 1A8), CD11b/Mac1 APC (Clone M1/70). Samples were acquired on LSR Fortessa (BD) and flow cytometry data analysing by using FlowJo (Treestar, Ashland, OR, USA).

Bone Marrow Transplantation Assay and Peripheral Blood Analysis

Donor cells were obtained from C56BL/6J mice (CD45.2). Recipient mice were C57Bl6W41/W41 (W41) mice as described previously (Kent *et al.*, 2009; Benz *et al.*, 2012). Recipient mice were sub-lethally irradiated with a single dose (400cGy) of Cesium irradiation and all transplants were performed by intravenous tail vein injection using a 29.5G insulin syringe. Single HSCs were deposited by FACS into 100µL of medium in a 96-well U-bottom plate. All liquid was subsequently mixed with extra 100µL of PBS and aspirated into the insulin syringe (avoiding air bubbles) and injected into the tail vein. For secondary transplantations, whole bone marrow was obtained from primary recipient by flushing tibiae and femurs with

PBS + 2%FCS. Red cell lysis was performed and an equivalent of one femur (~$2x10^7$ cells) of each donor mouse was transplanted into at least two secondary recipients.

PB samples were collected in EDTA coated microvette tubes (Sarstedt AGF & Co, Nuembrecht, Germany). Blood was collected from the tail vein at week 8, 12, 16, 20, 24, post-transplantation, unless otherwise stated. Red cell lysis was performed by using NH_4Cl and samples were subsequently analysed for repopulation levels as previously described (Kent et al. 2016; Wilson et al. 2015). Cells were stained for lineage markers using Ly6g BV421 (Clone 1A8), B220 APC (Clone RA3-6B2), CD3e PE (Clone 17A2), CD11b/Mac1 PE-Cy7 or BV605 (Clone M1/70), CD45.1 AF700 (Clone A20), CD45.2 FITC (Clone 104). All antibodies were obtained from Biolegend. Samples were acquired on LSR Fortessa (BD) and flow cytometry data analysing by using FlowJo (Treestar, Ashland, OR, USA).

Single cell RNA sequencing analysis

Single cell RNA sequencing (scRNA seq) analysis was performed as described previously in Picelli et al. 2014 (Smart-seq2). Single ESLAM HSCs were sorted by FACS into 96-well PCR plates containing lysis buffer (0.2% Triton X-100 (Sigma), RNase inhibitor (SUPERase, Thermofisher), nuclease-free water (Thermo Fisher)) Illumina Nextera XT DNA preparation kit was used to prepare the libraries, which were pooled and run on the Illumina Hi-Seq4000 at the CRUK Cambridge Institute Genomics Core. Cells from which low-quality libraries with insufficient sequencing depths were generated were excluded by setting the threshold of number of mapped reads to $>2*10^5$, with mapped reads comprising nuclear genes, mitochondrial genes and ERCCs. A minimum threshold of 20% for reads mapping to known genes was set, in order to exclude empty wells and dead cells. In addition, the threshold for reads mapping to mitochondrial genes was >0.2, to ensure a minimum of 20% of reads to map to non-mitochondrial genes. Protein-coding genes were extracted for further processing. GEO accession number: GSE160131.

Lentiviral transduction of mouse HSCs

7000 ESLAM HSCs cells were isolated and split between 4 wells (1750 cells/well) of a 96-well plate (Corning). Following their isolation, cells were kept in 50 µL of medium (StemSpan, 10%FCS, 20ng/mL IL-11) and were supplemented with polybrene (Sigma) and pHIV-ZsGreen

CSTVR lentivirus supplied by Dr Alasdair Russell from Cancer Research UK (CRUK). Plates were centrifuged at 600g for 30 minutes, at 30°C, to promote infection, before being transferred into a 37°C incubator. Two days after, cells were collected from the wells and resorted for viability (7AAD-). Live cells (4001) were transplanted into 6 sub-lethally irradiated CD45.1 W41 recipient mice (for an approximate dose of 615 cells/mouse) and monitored for donor chimerism as described above, and GFP expression.

Isolation of human CB HSCs and *in vitro* assays

Cord blood samples were obtained from Cambridge Blood and Stem Cell Biobank (CBSB) with informed consent from healthy donors in accordance with regulated procedures approved by the relevant Research and Ethics Committees. Mononuclear cells (MNCs) were isolated using Lymphoprep (Axis Shield PLC, Dundee, UK) or Pancoll lymphocyte separating medium (Pancoll, PAN Biotech, Aidenbach, Germany). Blood was mixed with equal volume of PBS and layered on Lymphoprep/Pancoll. Layered blood was centrifuged at 1400 rpm for 25 min, at room temperature with the brake off. The MNC layer was carefully aspirated and washed with PBS, to remove any separating medium trace. Red cell lysis was subsequently performed by using red cell lysis buffer (Biolegend, San Diego, CA, USA (Biolegend)). MNCs were depleted of differentiated hematopoietic cells by using the human CD34 microbead kit (Miltenyi Biotec, Bergisch Gladbach, Germany) with the following modifications: all cells were resuspended in 90 µL PBS, 2% FCS / 10^8 cells, CD34 Microbeads were used at 30 µL/10^8 cells and FcR Blocking Reagents at 30 µL/10^8 cells. Cells were separated using the AutoMACS cell separation technology (Miltenyi Biotec).
CD34 enriched cells were stained with CD34 APC-Cy7 (Clone HIT2, Biolegend), CD38 PE-Cy7 (Clone HIT2, Biolegend), CD45RA FITC or PE (Clone HI100, Biolegend), CD90 APC or PE (Clone 5E10, Biolegend or Biosciences respectively), CD49f PE-Cy5 (Clone GoH3, Biosciences) and Zombie Aqua (Biolegend) was used as a cell viability marker. HSCs were sorted as CD34$^+$, CD38$^-$, CD45RA$^-$, CD19$^-$, CD49f$^+$, CD90$^+$ on a BD FACS Aria fusion sorter at the NIHR Cambridge BRC Cell Phenotyping Hub facility. The single cells were sorted into individual wells of a 96-well U-bottom plate, each well having been preloaded with 100µL medium.

Liquid cultures and clone size determination of human LT-HSCs

Single HSCs were sorted into 96-well U-bottom plates and cultured in 100µL StemSpan SFEM (STEMCELL) supplemented with 100 units/mL Penicillin and 100µg/mL Streptomycin (Pen/Strep, Sigma-Aldrich), 2mM L-Glutamine (Sigma-Aldrich), 10^{-4}M 2-Mercaptoethanol and 20 ng/mL IL-11 (Biotechne, Abingdon, UK (Bio-techne)), 300ng/mL stem Cell Factor (SCF, R&D)(added when specified), 10% FCS (added when specified). Cell survival was assessed by visual inspection on day 10 (the sorting day is determined as day 0).

Xenotransplantation and Peripheral Blood Analysis

10,862 LT-HSCs were isolated from CD34 enriched CB and cultured into a single well (U-bottom 96-well plate) for 7 days as described above for the single cell culture. On day 7, cell number was assessed by visual inspection and cells were serially diluted in PBS as following: ~110 cells split into 5 recipients (~22 cell per mouse), ~440 cells split into 4 recipients (~110 cells per mouse), ~654 cells split into 3 recipients (~218 cells per mouse). NSG mice were sub-lethally irradiated with a single dose (2.4 Gy) by Cesium irradiation. Twenty-four hours later mice were anesthetised with isoflurane and injected intrafemorally as previously described [29].

PB samples were collected in EDTA coated microvette tubes (Sarstedt AGF & Co, Nuembrecht, Germany). Blood (~100µL) was collected from the tail vein at 8, 12, and 20 weeks post-transplantation. Mice were sacrificed 20 weeks post-transplantation and BM cells were isolated by flushing the injected femur with PBS/FCS. Blood was transferred into polystyrene tubes (Becton Dickinson) tubes and diluted 1:1 with 2%FCS in PBS. 1 mL of Lymphoprep (STEMCELL) was carefully layered at the bottom of the tube and the tubes were centrifuge for 25 min at 500g (brake off). MNCs were collected, washed with PBS and resuspended in 50µL of PBS/FCS and transferred into a 96 u-bottom plate (Falcon) to stain. Cells were stained with the following lineage markers: CD19/FITC (clone HIB19, Biolegend), GlyA/PE (clone HIR2, BD), CD45/PE-Cy5 (clone HI30, Biolegend), CD14/PE-Cy7 (clone M5E2, Biolegend), CD33/APC (clone P67.6, BD), CD19/AF700 (clone HIB19, Biolegend) , CD3/APC-Cy7 (clone HIT3a, Biolegend), CD45/BV510 (clone HI30, Biolegend). Samples were acquired on LSR Fortessa (BD) and flow cytometry data were analysed by using FlowJo v10 (FLOWJO LLC, Ashland, OR, USA). To detect human engraftment, two distinct antibodies against CD45 were used, and cells were considered human if positive for both ($CD45^{++}$). Mice were considered successfully

repopulated if the percentage of ($CD45^{++}$) ≥ 0.01% (and at least 30 cells were recorded in these gates).

Statistical analysis

Computational analyses were performed in the R programming environment (version 3.6.3). Raw data was processed using the Seurat tool (version 3.2.0)(Butler *et al.*, 2018; Stuart *et al.*, 2019). The recommended standard processing pipeline was applied to perform log-normalisation (default settings) and identify highly variable genes (nfeatures=10,000). Subsequently, expression values were scaled using default parameters. Dimensionality reduction, including principal component analysis (PCA) and Uniform Manifold Approximation and Projection (UMAP) was performed using default Seurat tools. Differential gene expression was performed using negative binomial generalised linear models, as implemented by DESeq2 (version 1.26.0)(Love, Huber and Anders, 2014). Genes with adjusted *p-value* <0.05 and logFC >1.5 were considered significantly differentially expressed (Benjamini-Hochberg corrected). Cell cycle scoring was performed based on average expression of key cell cycle genes, as described previously(Tirosh *et al.*, 2016). Similarly, gene set scoring was computed for previously described HSC proliferation quiescence signatures (Venezia *et al.*, 2004). Such scoring was also applied to gene sets, previously identified as upregulated and downregulated in cells in a G0 state(Cheung and Rando, 2013). Batch effect testing and correction was performed to inform any potential influence of technical bias. Normalisation and variable gene scoring were computed for each batch separately, using variance stabilising transformation. Subsequently, separate batches were integrated using canonical correlation analysis (CCA) by computing integration anchors (parameters: dims = 1:30 and k.filter = 10)(Stuart *et al.*, 2019). A very limited batch correction was identified between Day 1 and Day 2 batches (Supplementary figure 1B). However, full data integration introduced extensive over-correction and downstream analysis was performed without batch correction (data not shown). All data visualisation was computed in R. To inspect downstream IL-11 signalling, the following curated pathways gene sets, as outlined in the gene set enrichment analysis database (Mootha *et al.*, 2003; Subramanian *et al.*, 2005) were retrieved: I) KEGG_JAK_STAT_SIGNALING_PATHWAY (M17411); II) BIOCARTA_NFKB_PATHWAY (M15285); III) HALLMARK_PI3K_AKT_MTOR_SIGNALING (M5923); KEGG_MAPK_SIGNALING_PATHWAY (M10792). Similarly,

KEGG_REGULATION_OF_AUTOPHAGY (M6382) and REACTOME_CELLULAR_SENESCENCE (M27188) were used. All gene sets were subsequently manually curated to exclude ligand and receptor -associated genes (Supplementary Table 1).

To compute gene ontology (GO) and KEGG pathway enrichment, gene symbols were converted to Entrez gene identifiers, using the mouse genome annotation database (org.Mm.eg.db, version 3.10.0). GO terms were extracted from the GO annotation database (GO.db, version 3.10.0). GO term enrichment and KEGG pathways analysis was computed using the Limma package (version 3.42.2). An adjusted *p-value* < 0.05 cutoff was set to determine GO term or KEGG pathway enrichment. Genes identified as significantly differentially expressed between cell types were used conduct pathway enrichment.

Gene set enrichment analysis (GSEA) was performed using the UC San Diego-Broad Institute GSEA software (version 4.0.3) (Mootha *et al.*, 2003; Subramanian *et al.*, 2005). Pre-ranked gene lists were computed based on differentially expressed genes. GSEA was computed using multiple databases, including GO biological processes, KEGG pathways and the Reactome database. Analysis parameter were set as follows: 1000 permutations, weighted enrichment, minimum 15 and maximum 500 genes annotated to gene set.

Supplementary appendix 1: Single-cell time-lapse imaging of single HSCs in hibernation cultures.

Supplementary table 1: JAK/STAT, MAPK, NKFB, PI3K/AKT gene sets

JAK/STAT, MAPK, NFKB, PI3K/AKT gene sets manually curated to exclude ligand- and receptor-associated genes. See also Supplementary Figure 1.

Supplementary Figure 1: Molecular profiling of HSC, hibHSC, HSC+SCF, hibHSC+SCF, related to Figure 5

(A) UMAPs depicting (I) cell type (HSC, blue dots; hibHSC, red dots; HSC+SCF, green dots; hibHSC+SCF, orange dots); (II) batches (batch 0, orange dots; batch 1, blue dots; batch 2, green dots; batch 3, pink dots); days batches were sequenced (day 1, purple dots; day 2, blue dots; day 3, orange dots). (B) MolO gene relative expression in HSC, HSC+SCF, hibHSC, hibHSC+SCF (C) Left panel, PCA of all cells coloured by computationally assigned cell cycle

category, right panel, the 4 cellular states are projected onto the PCA. The PCA was computed using cell cycle genes exclusively.

Supplementary Figure 2: HSC proliferation and quiescence signature genes, related to Figure 5

(A) Violin plots displaying individual proliferation scores by physiological condition (B) Gene Set Enrichment Analysis of the HSC proliferation signature (Venezia *et al.*, 2004), computed using DE genes of direct comparison of HSCs and HSC+SCF. (C) Heatmap of previously identified HSC-specific quiescence signature genes (Venezia *et al.*, 2004), sorted by cell type. (D, E) Gene sets upregulated in G0 cell populations and gene sets downregulated (anti-G0) were used to compute G0 and anti-G0 gene signature scores (Cheung and Rando, 2013). These were projected onto the UMAP depictions (see Figure 5A or Supplementary Figure 1A for reference).

Supplementary Figure 3: Autophagy, senescence, and IL-11RA gene signatures, related to Figure 5

(A) Autophagy gene signature scores projected onto the UMAP landscape and summarised in form of a violin plot. (B) Senescence gene signature depicted as described in (A). (C) Violin plot of the IL-11 receptor gene (IL-11RA1) and gene signature scores for core signalling pathways stimulated by IL-11. Includes: PI3K, NKFB, MAPK and JAK-STAT. (D) Violin plots of top differentially expressed PI3K pathway genes. (E) Top differentially expressed genes of the NF-kB pathway.

Supplementary Figure 4: Specific gene sets are altered during hibernation and SCF-stimulation, related to Figure 6

(A) Violin plots of normalised gene expression of the 13 upregulated genes in SCF-stimulated cells (HSC+SCF, hibHSC+SCF). (B) Volcano plot of differentially expressed genes, comparing HSCs and hibHSC. DE genes are marked in red (logFC>1 and adj *p-value* <0.05, Benjamini-Hochberg corrected). (C) Violin plots of normalised gene expression of genes of interest, downregulated in hibHSC compared to HSC.

Supplementary Figure 5: Genes of interests enriched in hibHSCs, related to Figure 6

UMAPs of selected genes of interests enriched in hibHSC (manually selected from DE gene set. The large majority of the hibHSCs appear in the upper right portion of the plot (see Figure 5A or Supplementary Figure 1A for reference).

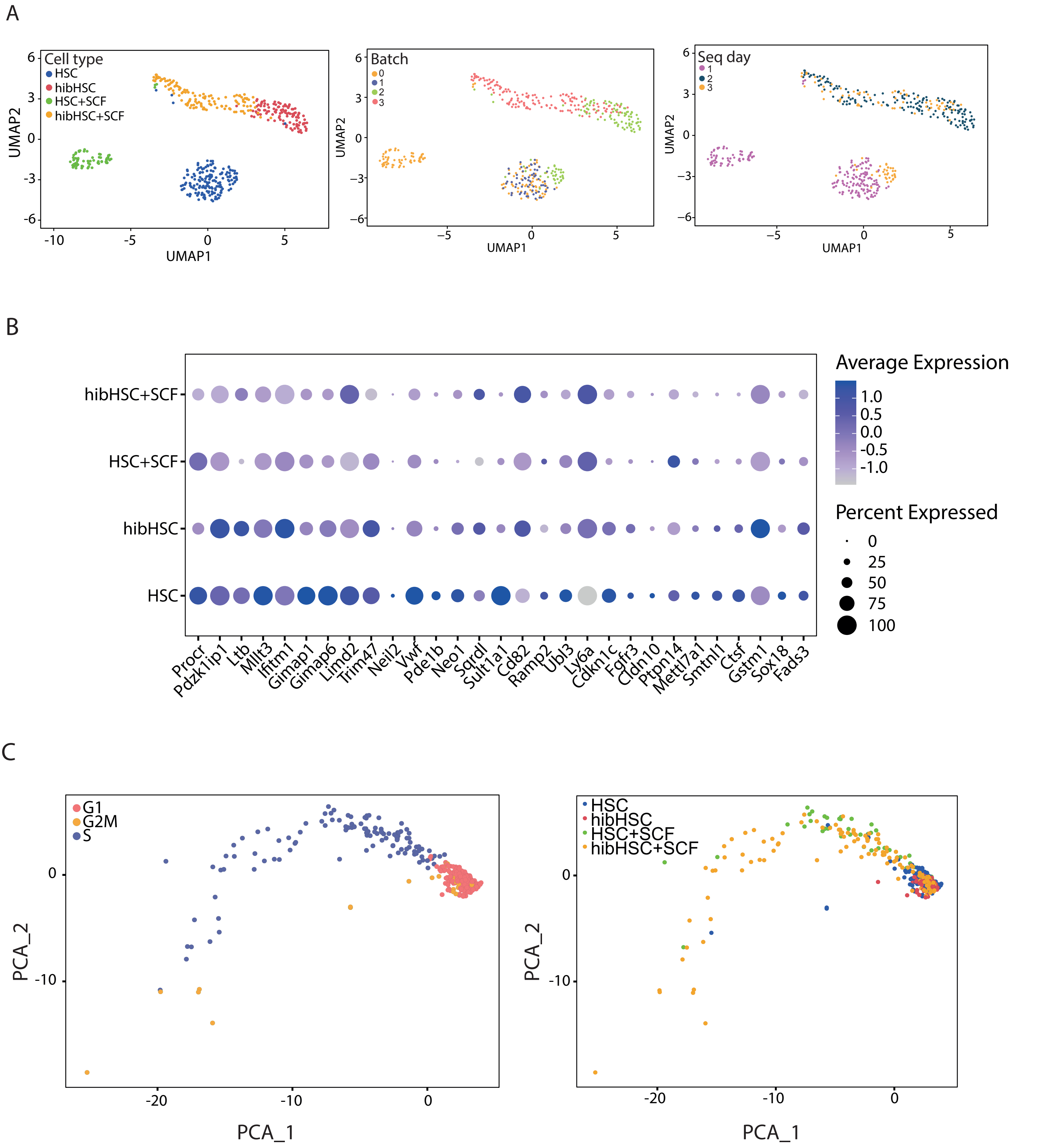

A
Cell type
HSC
hibHSC
HSC+SCF
hibHSC+SCF
UMAP1
UMAP2
Batch
0
1
2
3
Seq day
1
2
3
B
Average Expression
1.0
0.5
0.0
-0.5
-1.0
Percent Expressed
0
25
50
75
100
hibHSC+SCF
HSC+SCF
hibHSC
HSC
Procr
Pdzk1ip1
Ltb
Mllt3
Ifitm1
Gimap1
Gimap6
Limd2
Trim47
Neil2
Vwf
Pde1b
Neo1
Sqrdl
Sult1a1
Cd82
Ramp2
Ubl3
Ly6a
Cdkn1c
Fgfr3
Cldn10
Ptpn14
Mettl7a1
Smtnl1
Ctsf
Gstm1
Sox18
Fads3
C
G1
G2M
S
HSC
hibHSC
HSC+SCF
hibHSC+SCF
PCA_1
PCA_2

S1

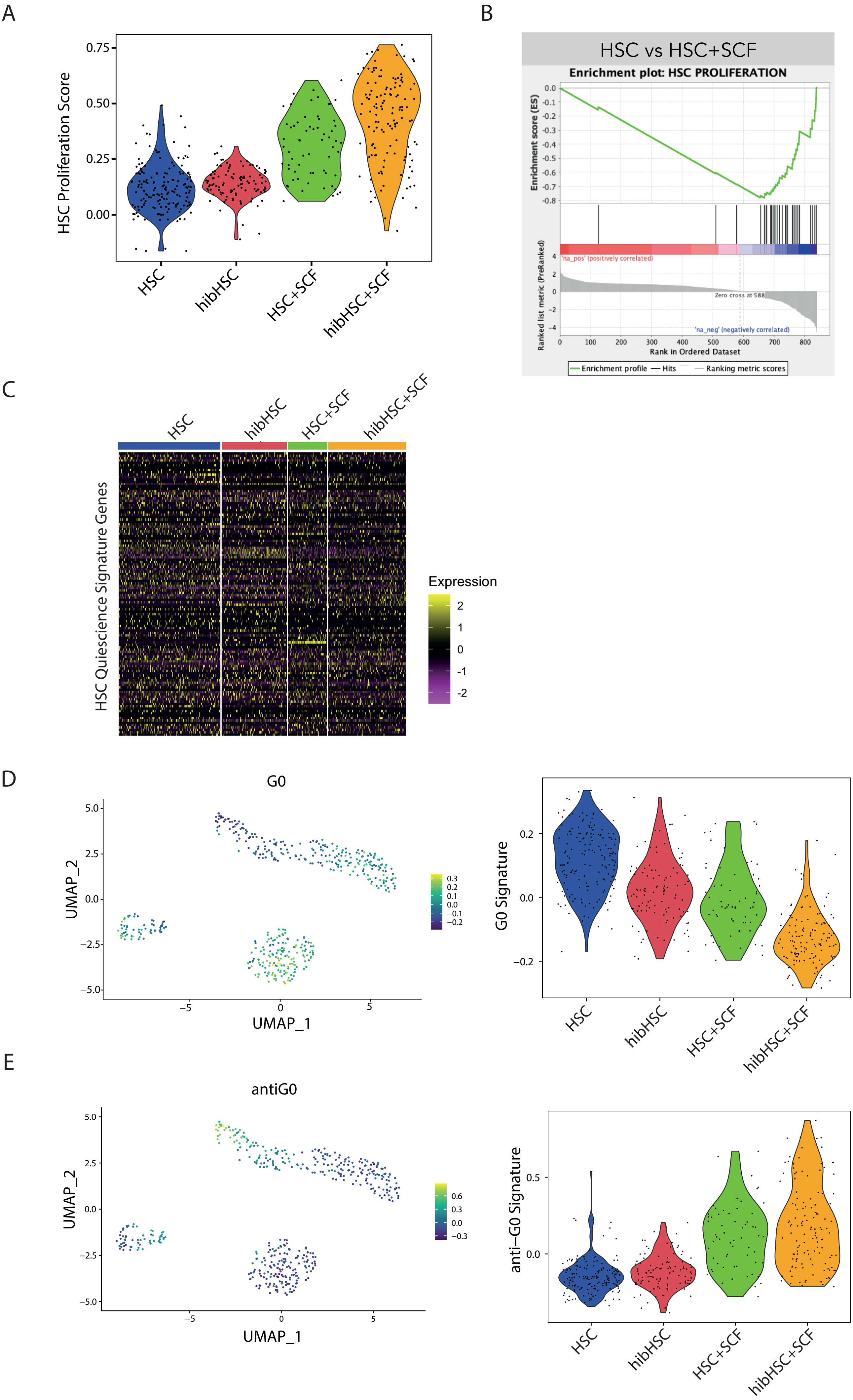
A
HSC Proliferation Score
HSC
hibHSC
HSC+SCF
hibHSC+SCF
B
HSC vs HSC+SCF
Enrichment plot: HSC PROLIFERATION
Enrichment score (ES)
Ranked list metric (PreRanked)
'na_pos' (positively correlated)
Zero cross at 588
'na_neg' (negatively correlated)
Rank in Ordered Dataset
Enrichment profile — Hits — Ranking metric scores
C
HSC Quiescience Signature Genes
Expression
D
G0
UMAP_2
UMAP_1
G0 Signature
E
antiG0
anti−G0 Signature

S2

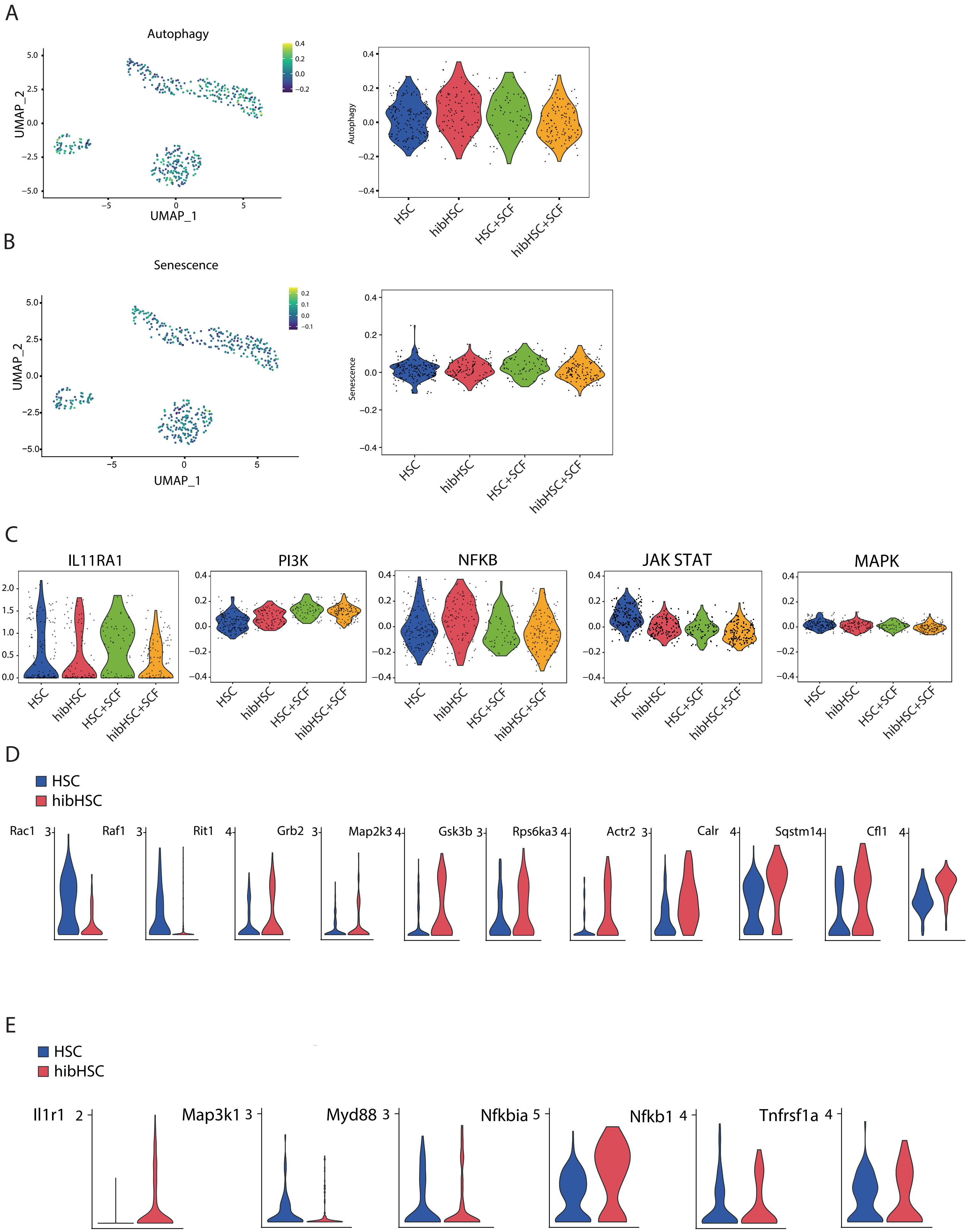
A
Autophagy
UMAP_2
UMAP_1
Autophagy
HSC
hibHSC
HSC+SCF
hibHSC+SCF
B
Senescence
UMAP_2
UMAP_1
Senescence
C
IL11RA1
PI3K
NFKB
JAK STAT
MAPK
D
HSC
hibHSC
Rac1
Raf1
Rit1
Grb2
Map2k3
Gsk3b
Rps6ka3
Actr2
Calr
Sqstm1
Cfl1
E
Il1r1
Map3k1
Myd88
Nfkbia
Nfkb1
Tnfrsf1a

S3

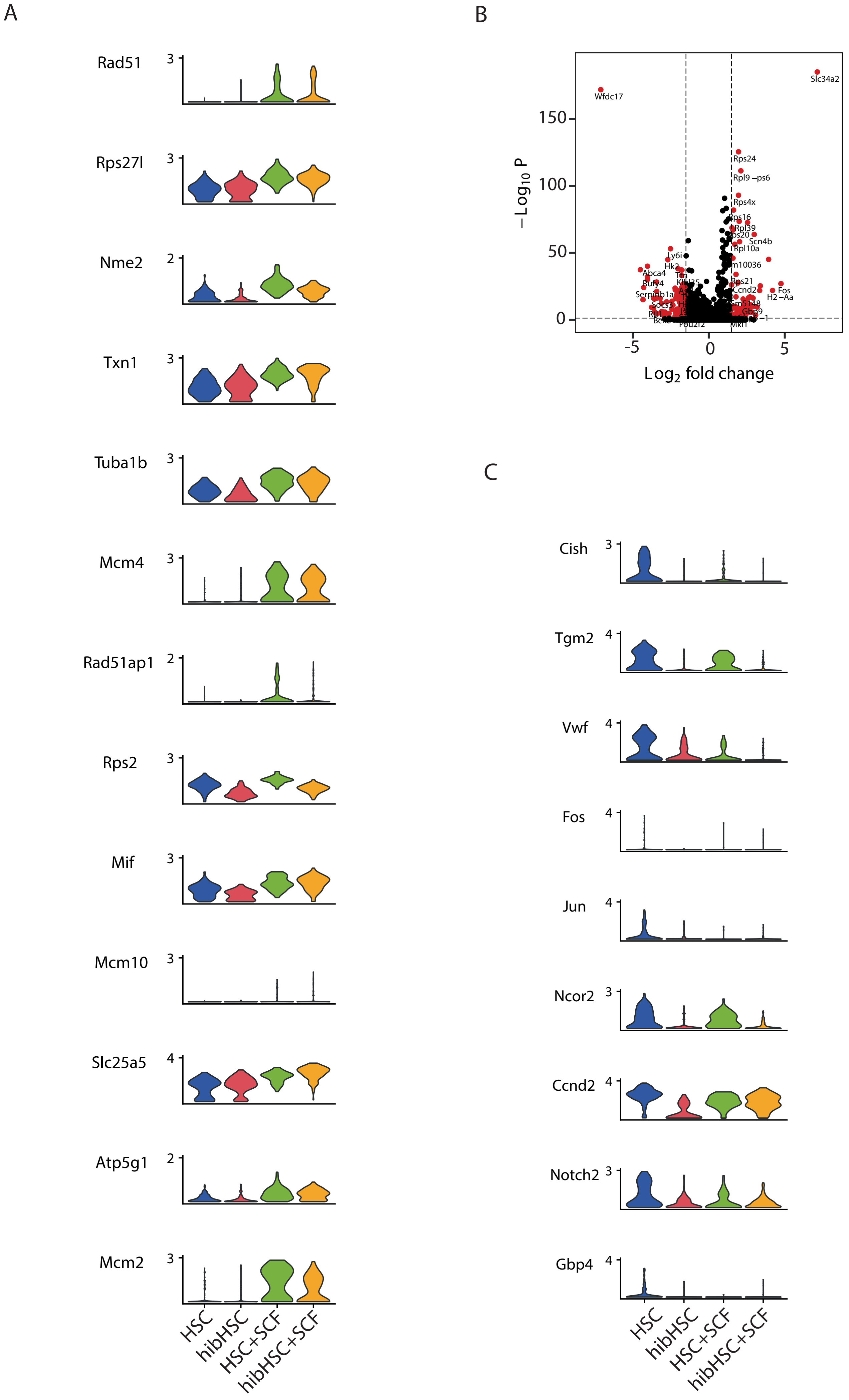
A
B
C
Rad51
Rps27l
Nme2
Txn1
Tuba1b
Mcm4
Rad51ap1
Rps2
Mif
Mcm10
Slc25a5
Atp5g1
Mcm2
HSC
hibHSC
HSC+SCF
hibHSC+SCF
−Log10 P
Log2 fold change
Wfdc17
Slc34a2
Rps24
Rpl9 −ps6
Rps4x
Rps16
Rpl39
Scn4b
Rpl10a
Ly6i
Hk2
Abca4
Rufy4
Serpinb1a
Socs3
Ccnd2
Rps21
Fos
H2−Aa
Gbp9
Pou2f2
Cish
Tgm2
Vwf
Fos
Jun
Ncor2
Ccnd2
Notch2
Gbp4

S4

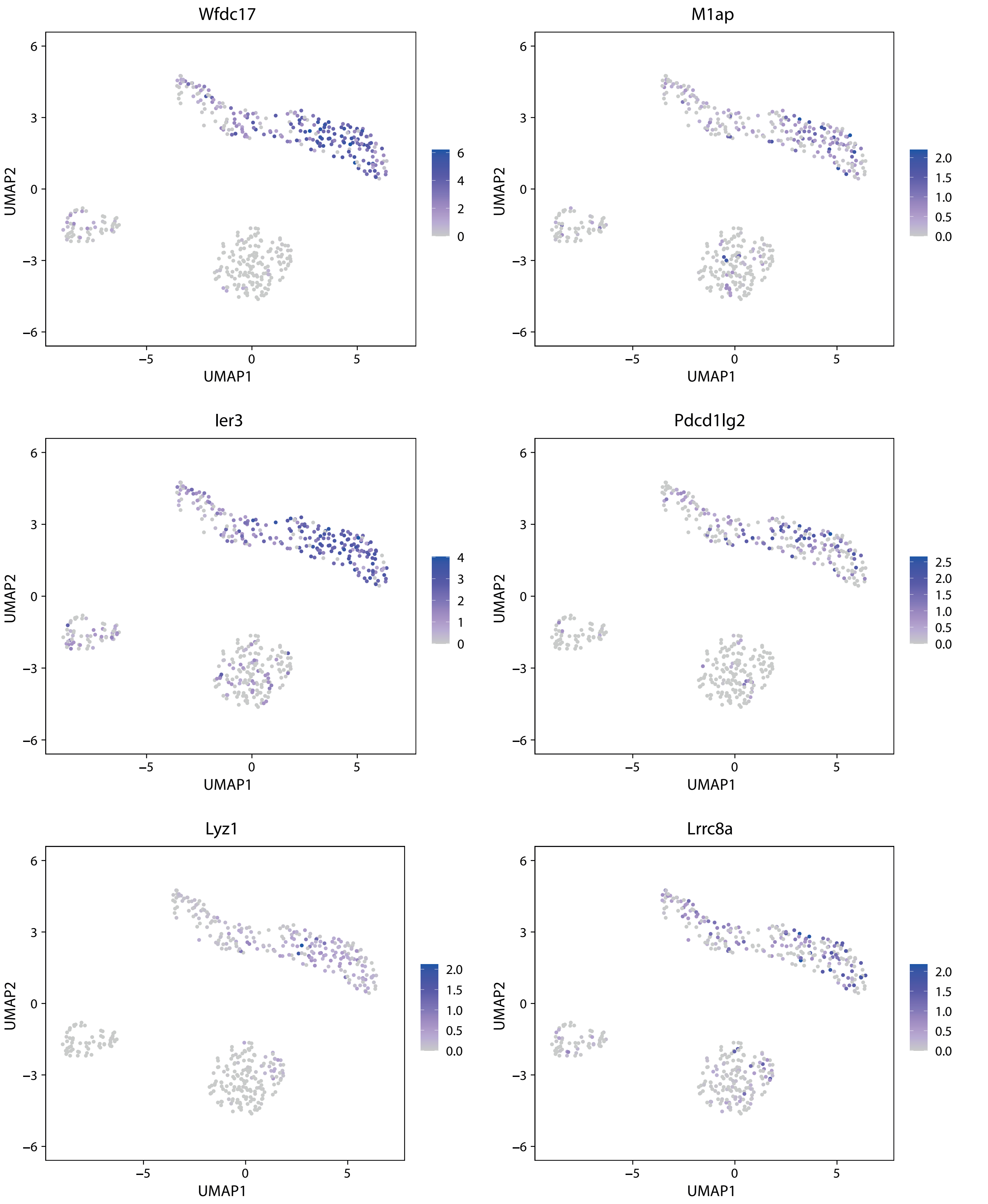
Wfdc17
M1ap
Ier3
Pdcd1lg2
Lyz1
Lrrc8a
UMAP1
UMAP2
6
4
2
0
2.0
1.5
1.0
0.5
0.0
4
3
2
1
0
2.5
2.0
1.5
1.0
0.5
0.0
−5
0
5
−6
−3
3
6

S5
