# Supplementary material for: Hematopoietic stem cells retain functional potential and molecular identity in hibernation cultures
Source: Stem Cell Reports. 2021 May 6;16(6):1614–28. doi: 10.1016/j.stemcr.2021.04.002 (PMC8190576; doi:10.1016/j.stemcr.2021.04.002)
Supplement: Document S2. Article plus supplemental information [file mmc4.pdf]

Stem Cell Reports
Resource
OPEN ACCESS

Hematopoietic stem cells retain functional potential and molecular identity in hibernation cultures

Caroline A. Oedekoven,[1,2,4] Miriam Belmonte,[1,2,4] Daniel Bode,[1,2] Fiona K. Hamey,[1,2] Mairi S. Shepherd,[1,2] James Lok Chi Che,[1,2] Grace Boyd,[3] Craig McDonald,[1,2] Serena Belluschi,[1,2] Evangelia Diamanti,[1,2] Hugo P. Bastos,[1,2] Katherine S. Bridge,[3] Berthold Göttgens,[1,2] Elisa Laurenti,[1,2,5] and David G. Kent[1,2,3,5,*]
[1]Wellcome MRC Cambridge Stem Cell Institute, University of Cambridge, Hills Road, Cambridge CB2 0XY, UK
[2]Department of Haematology, University of Cambridge, Cambridge CB2 0XY, UK
[3]York Biomedical Research Institute, Department of Biology, University of York, York YO10 5DD, UK
[4]These authors contributed equally
[5]These authors contributed equally
*Correspondence: david.kent@york.ac.uk
https://doi.org/10.1016/j.stemcr.2021.04.002

SUMMARY

Advances in the isolation and gene expression profiling of single hematopoietic stem cells (HSCs) have permitted in-depth resolution of their molecular program. However, long-term HSCs can only be isolated to near purity from adult mouse bone marrow, thereby precluding studies of their molecular program in different physiological states. Here, we describe a powerful 7-day HSC hibernation culture system that maintains HSCs as single cells in the absence of a physical niche. Single hibernating HSCs retain full functional potential compared with freshly isolated HSCs with respect to colony-forming capacity and transplantation into primary and secondary recipients. Comparison of hibernating HSC molecular profiles to their freshly isolated counterparts showed a striking degree of molecular similarity, further resolving the core molecular machinery of HSC self-renewal while also identifying key factors that are potentially dispensable for HSC function, including members of the AP1 complex (*Jun*, *Fos*, and *Ncor2*), *Sult1a1* and *Cish*. Finally, we provide evidence that hibernating mouse HSCs can be transduced without compromising their self-renewal activity and demonstrate the applicability of hibernation cultures to human HSCs.

INTRODUCTION

The blood-forming system is sustained by a rare subset of hematopoietic stem cells (HSCs) with the potential to differentiate into all mature blood cell types and to create equally potent daughter HSCs to maintain tissue homeostasis (Doulatov et al., 2012; Eaves, 2015; Ganuza et al., 2020; Laurenti and Göttgens, 2018). As the seed cells for the blood system, their clinical potential for cellular therapies is vast and the need to understand their molecular program in different physiological states is critical for their therapeutic application. Recently, cell culture conditions have been reported to produce large numbers of functional mouse and human HSCs (hHSCs) (Fares et al., 2017; Wilkinson et al., 2019) but, in all cases, the substantial majority of cells produced are non-HSCs (Bak et al., 2018; Gundry et al., 2016; Shepherd and Kent, 2019; Wagenblast et al., 2019).

In the absence of robust purification strategies for functional HSCs in culture, it becomes virtually impossible to study the molecular profile of HSCs removed from their *in vivo* microenvironment. Previous studies have highlighted the potential for retaining long-term HSC (LT-HSC) function in cultures with low amounts of proliferation in the absence of excessive cytokine-induced signaling (Kobayashi et al., 2019; Yamazaki et al, 2006, 2009), although these cultures were still predominantly non-HSCs. An *in vitro* system that could retain highly purified single HSCs would offer the potential to molecularly profile niche-independent HSCs and to resolve the essential components of self-renewal *in vitro*.

Here, we describe such a system, demonstrating that fully functional mouse LT-HSCs can be maintained in minimal cytokine conditions over a period of 7 days without undergoing cell division. This novel cell culture system preserves the core features of HSCs, including the speed of quiescence exit, subsequent cell-cycle kinetics, mature cell production, and HSC self-renewal activity in serial transplantation assays. The functional properties of these hibernating HSCs are virtually indistinguishable from freshly isolated HSCs and molecular profiling by single-cell RNA sequencing (scRNA-seq) shows a high degree of overlap with freshly isolated HSCs, but also reveals a number of molecular changes that identify genes potentially dispensable for retaining HSC function.

RESULTS

Single LT-HSCs can retain multipotency *in vitro* under minimal cytokine stimulation

Previous studies suggested that stem cell factor (SCF) and thrombopoietin (TPO) are essential for HSC self-renewal and proliferation, but potentially dispensable for stem

This is an open access article under the CC BY license (http://creativecommons.org/licenses/by/4.0/).

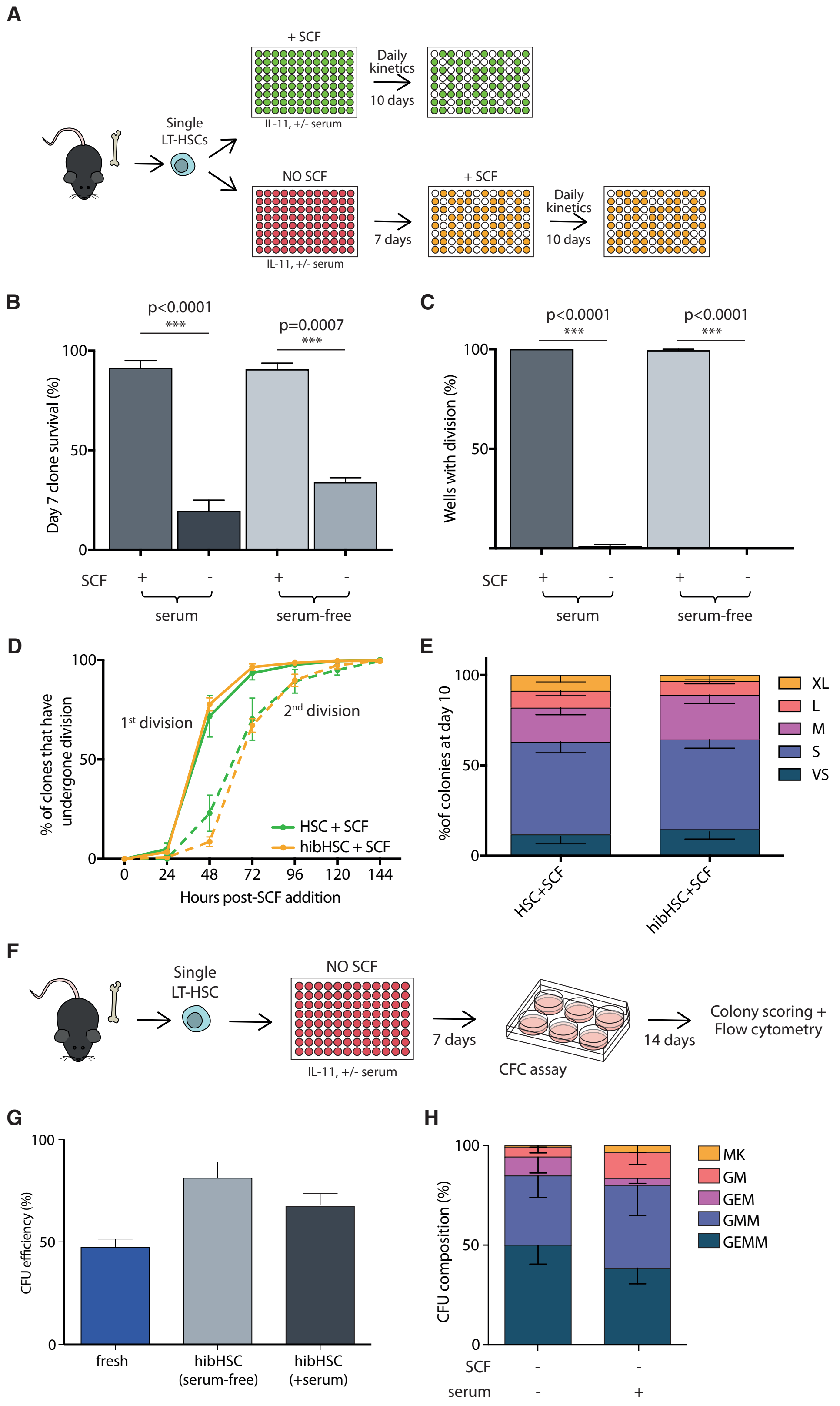

(legend on next page)

cell maintenance (Yamazaki et al., 2006, 2009; De Graaf and Metcalf, 2011). A number of studies use gp130 family members (e.g., interleukin-11 [IL-11], IL-6) in HSC maintenance conditions, including our own studies which typically use 20 ng/mL of IL-11 alongside 300 ng/mL of SCF (Kent et al., 2008b; Kent et al., 2013; Shepherd et al., 2018). To test the absence of SCF and TPO, we cultured single mouse bone marrow $CD45^{+}EPCR^{+}CD48^{-}CD150^{+}Sca1^{high}$ LT-HSCs, which are ~60% functional HSCs by single-cell transplantation (Wilson et al., 2015), in the presence of 20 ng/mL IL-11 alone in both serum-containing (Kent et al., 2013; Shepherd et al., 2018) and serum-free conditions (Wilkinson et al., 2019) (Figure 1A). Between 20% and 40% of single LT-HSCs survived 7 days of culture (Figure 1B), making them considerably more resilient to cytokine depletion than single sorted progenitor cell fractions ($Lin^{-}Sca1^{+}c\text{-}Kit^{+}$), where no cells survived past 2 days (data not shown). Interestingly, 99.2% (634 of 639 cells) of the surviving input LT-HSCs were maintained as single cells for the 7-day period (Figure 1C), and single-cell time-lapse imaging and tracking confirmed that cells did not undergo division followed by death of one daughter cell (Video S1). Together this prompted us to term the minimal cytokine condition as a "hibernation" condition, similar to the cellular state of LT-HSCs described after the addition of lipid raft inhibitors (Yamazaki et al., 2006).

To assess the functional potential of single LT-HSCs in the hibernation condition (hibHSCs), 300 ng/mL SCF was added to mirror cytokine combinations previously applied to freshly isolated LT-HSCs (Kent et al., 2008a; Kent et al., 2013; Shepherd et al., 2018). Time to first and second division was indistinguishable from freshly isolated LT-HSCs receiving SCF (Figure 1D), and clonal proliferation and survival over the subsequent 10 days was also similar, as indicated by clone size distribution being nearly identical to freshly isolated HSCs stimulated for 10 days (Figure 1E). In accordance with this, single hibHSCs also retained their multipotency in colony-forming cell (CFC) assays (Figures 1F and 1G) and 60%–70% of single cells generated at least three different lineages (Figure 1H), as determined by flow cytometry. Together, these data suggest that HSCs surviving cytokine depletion exist in a state of prolonged hibernation and can be revived to function indistinguishably from freshly isolated HSCs.

Hibernating HSCs are fully functional in transplantation assays

To assess whether cells cultured in the absence of SCF or TPO retained their HSC self-renewal expansion capability, single day 7 hibHSCs were transplanted and their repopulation capacity was compared with freshly isolated HSCs (Figure 2A); 62.5% (15/24) and 45.8% (13/29) of primary

Figure 1. Absence of SCF and TPO maintains HSCs as single multi-potent cells *in vitro*

(A) Single $CD45^{+}EPCR^{+}CD48^{-}CD150^{+}Sca1^{high}$ LT-HSCs were sorted into individual wells and cultured in the presence of IL-11, in serum-supplemented or serum-free medium and in the presence or absence of SCF. For SCF-supplemented cultures (green plate), daily cell counts were performed for 10 days. For cultures only containing IL-11 (red plate), HSCs were supplied with SCF on day 7 post-isolation after which daily cell counts were performed for an additional 10 days. In all cases, clone size was assessed at day 10 post-SCF addition.

(B) HSC survival is decreased in the absence of SCF compared with SCF-supplemented medium (+serum/+SCF n = 355, 5 biological replicates; +serum/−SCF n = 1,722, 7 independent experiments; −serum/+SCF, N = 144, 2 independent experiments, −serum/−SCF n = 284, 3 independent experiments).

(C) Numbers of wells with >2 cells were scored to determine the number of clones that had divided. At day 7 post-isolation, only culture conditions without SCF maintained HSCs as single cells.

(D) Cell division kinetics post-SCF addition. Entry into cell cycle was comparable between freshly isolated HSCs (green solid line) and cells that had been maintained as single cells for 7 days (orange solid line) in serum-supplemented media. Time to subsequent cell division (dotted lines) was not significantly different between conditions (SCF added at day 0, n = 355, 5 independent experiments; SCF added at day 7, n = 1,722, 7 independent experiments).

(E) Colony size was measured on day 10 post-SCF addition and no difference in clone size distribution was observed between HSCs cultured in the presence of SCF from day 0 and post-hibernation HSCs (day 7 + 10).

(F) Single LT-HSCs were cultured for 7 days in IL-11 alone, in serum-supplemented or serum-free medium. After 7 days, single hibernating LT-HSCs were individually transferred into a cytokine-rich methylcellulose CFC assay and cultured for an additional 14 days. On day 14, lineage composition of individual colonies was assessed by flow cytometry.

(G) Colony-forming efficiency for freshly isolated single LT-HSCs, single LT-HSCs cultured in serum-supplemented and serum-free hibernating cultures (fresh, n = 300, 3 biological replicates; serum-free, n = 121, 5 independent experiments; +serum, n = 230, 6 independent experiments).

(H) Colony subtype analysis showed that the majority of single cells (~80%) generated colonies of at least three lineages in colony-forming unit (CFU) assays (hibHSC serum-free, n = 70, 4 independent experiments; hibHSC + serum, n = 166, 3 independent experiments). Colonies were defined as MK (containing cells positive for megakaryocyte marker CD41), GM (containing cells positive for granulocyte/monocyte markers Gr1 and CD11b), GEM (positive for GM and erythrocyte markers Gr1, CD11b, and Ter-119), GMM (positive for GM and MK markers), and GEMM (positive for GM, MK, and E markers), as described in the Experimental procedures. Bars show mean with SEM. Unpaired t test: *p < 0.05, **p < 0.01, ***p < 0.001.

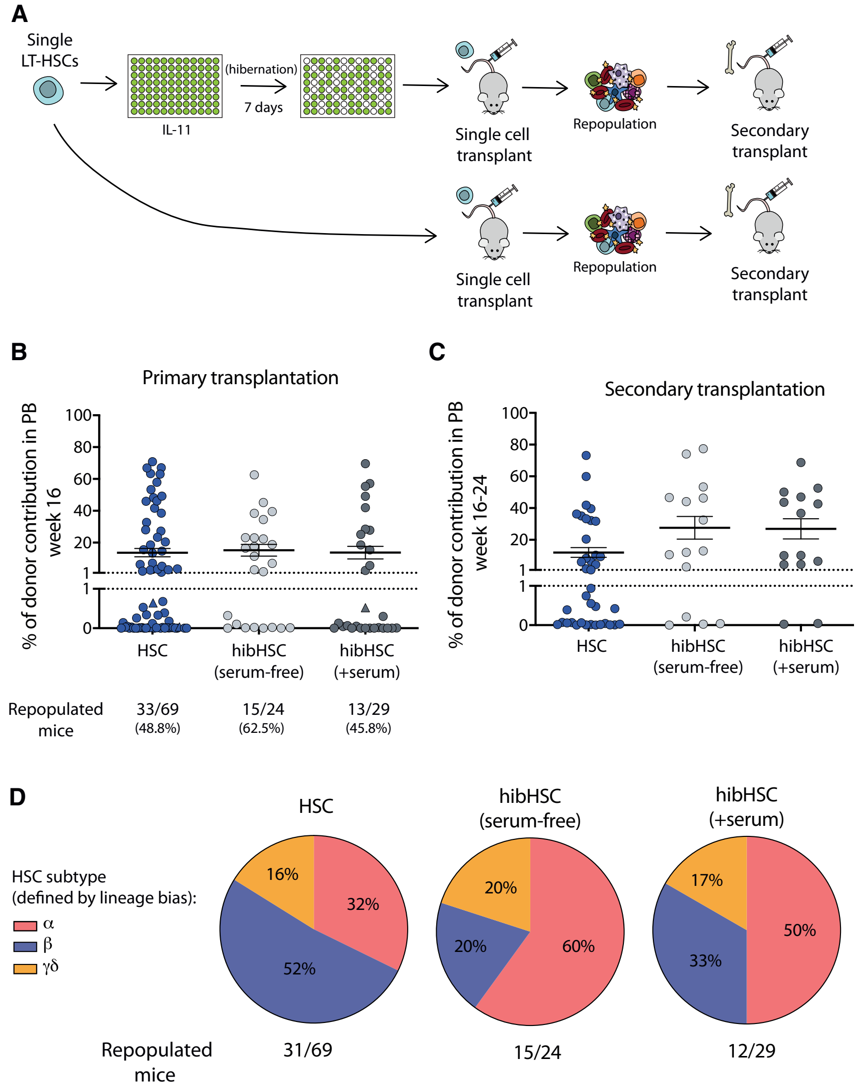

Figure 2. Hibernating HSCs maintain *in vivo* functional activity

(A) HSCs were cultured in hibernation conditions in either serum-supplemented or serum-free medium. Single fresh or day 7 hibernating LT-HSCs were transplanted into W41-CD45.1 recipients (fresh n = 69; serum-free n = 24; +serum n = 29). Secondary transplantations were undertaken in all mice with donor engraftment (>1%) at 16–24 weeks post-transplantation.

(B and C) Graphs show percent donor chimerism in the peripheral blood of primary (B) and secondary (C) recipient mice at 16–24 weeks post-transplantation. Recipients with chimerism >1% and at least 0.5% of GM, B, and T cells were considered to be repopulated. (Triangles represent mice where chimerism reached >1% at weeks 20–24 post-transplantation but had not done so by 16 weeks.)

(D) No significant difference was observed in the balance of mature cell outputs between freshly isolated and post-hibernation HSCs. Based on donor myeloid (M) to lymphoid (L) ratio at 16 weeks in primary recipients, the founder HSC was retrospectively assigned one of the following subtypes: α (alpha, M:L > 2), β (beta, M:L > 0.25 < 2), γ (gamma, M:L < 0.25), δ (delta, M:L < 0.25 and failure to contribute to myeloid lineage past 16 weeks) in accordance with Dykstra et al. (2007) (HSC n = 31/69; hibHSC (+serum) n = 12/29; hibHSC (serum-free) n = 15/24).

recipients transplanted with single hibHSCs (without serum and with serum, respectively) had >1% multi-lineage donor chimerism at 16–24 weeks post-transplantation compared with 48.8% (33/69) of freshly isolated HSCs (Figure 2B). Secondary transplantation efficiency was also high (Figure 2C), suggesting that the period of 7 days *in vitro* had no impact on HSC self-renewal. This was further supported by the observation of no significant differences in mature cell production between hibHSCs and freshly isolated HSCs, as determined by the relative proportions of the HSC subtype produced in single-cell transplantation experiments (Figure 2D). Notably, despite these high functional purities, the total yield of functional HSCs was slightly lower considering that some HSCs do not survive hibernation. These data provide formal evidence that, following 7 days of SCF and TPO depletion and in the complete absence of a supportive stem cell niche, LT-HSCs can retain full functional potential as assessed by serial transplantation.

High CD150 expression prospectively enriches for resilient HSCs

Since only a proportion of phenotypic LT-HSCs survive hibernation conditions, we used flow cytometric index-sort data to determine whether levels of specific cell surface markers might associate with survival. Expression levels of the SCF receptor (c-Kit) did not select for surviving HSCs, while higher CD45 and EPCR expression were modestly increased on hibHSCs compared with cells that did not survive hibernation conditions (data not shown). High CD150 expression strongly associated with higher survival at day 7 (Figure 3A). To verify whether CD150 could be used to prospectively enrich for resilient HSCs, single LT-HSCs were sorted as $CD150^{mid}$ or $CD150^{high}$

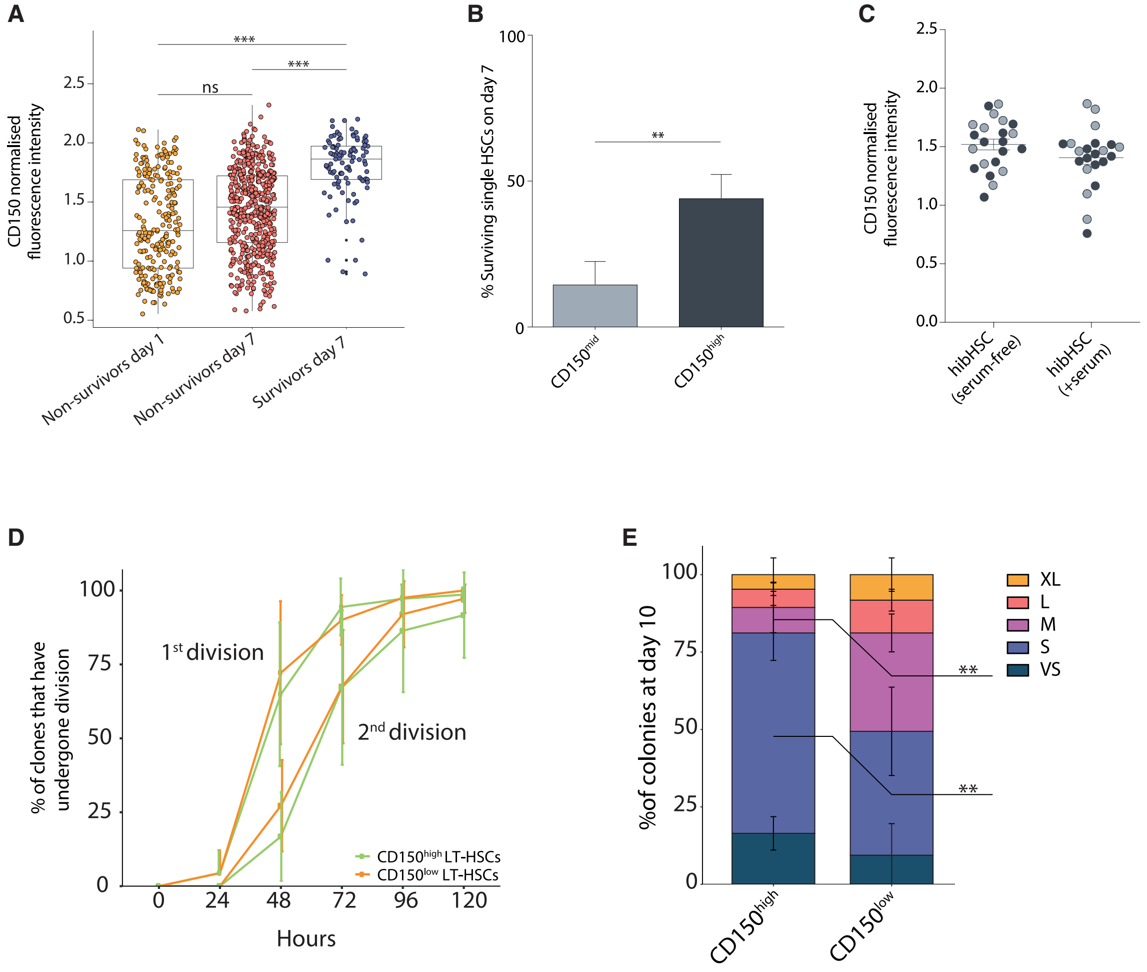

Figure 3. Higher expression of CD150 identifies resilient LT-HSCs

(A) Flow cytometric index-sort data were used to determine the CD150 expression level of LT-HSCs at the time of isolation. Cells that did not survive at day 1 and day 7 were compared with those that survived out to day 7, with the latter population of cells correlating with higher CD150 expression. A boxplot shows the median with interquartile range. Vertical lines represent outermost quartiles. Black dots, if present, are extreme outliers. Unpaired t test: $^{*}p < 0.05$, $^{**}p < 0.01$, $^{***}p < 0.001$.

(B) Prospectively sorted CD150^{high} LT-HSCs show 4.2-fold higher survival than CD150^{mid} LT-HSCs (n = 480, 5 independent experiments). Paired two-tailed t test.

(C) Hibernating HSCs in serum-free and serum-supplemented conditions were transplanted, and their CD150 levels retrospectively assessed. Cells able to repopulate a recipient (black) did not differ in initial CD150 expression levels compared with cells unable to repopulate (gray).

(D) HSCs with high or low expression of CD150 were determined using index-sorting data from freshly isolated HSCs that were cultured for 7 days in serum-free medium supplemented with 20 ng/mL IL-11 and 300 ng/mL SCF. Three biological replicates were analyzed, and in each case the top third and bottom third of CD150 expressers were analyzed as CD150^{high} and CD150^{low}, respectively. Daily cell counts were performed to assess cell division kinetics. Entry into cell cycle and the second division were not significantly altered between CD150^{high} and CD150^{low} LT-HSCs.

(E) Using the same experimental data from Figure 3D, colony sizes from single LT-HSCs were measured on day 10 and clone sizes from single LT-HSCs with high expression of CD150 were significantly reduced compared with those with low CD150 expression (bars show mean with SD. Sidak's multiple comparison test: $^{**}p < 0.01$).

and cultured in hibernation conditions. CD150^{high} HSCs show significantly higher (4.2-fold) survival on day 7 compared with CD150^{mid} HSCs, confirming that CD150 expression can enrich for phenotypic LT-HSCs that could survive hibernation conditions (Figure 3B). We next assessed whether CD150 levels on surviving LT-HSCs associated with successful transplantation and found no significant differences in CD150 intensity between HSCs that

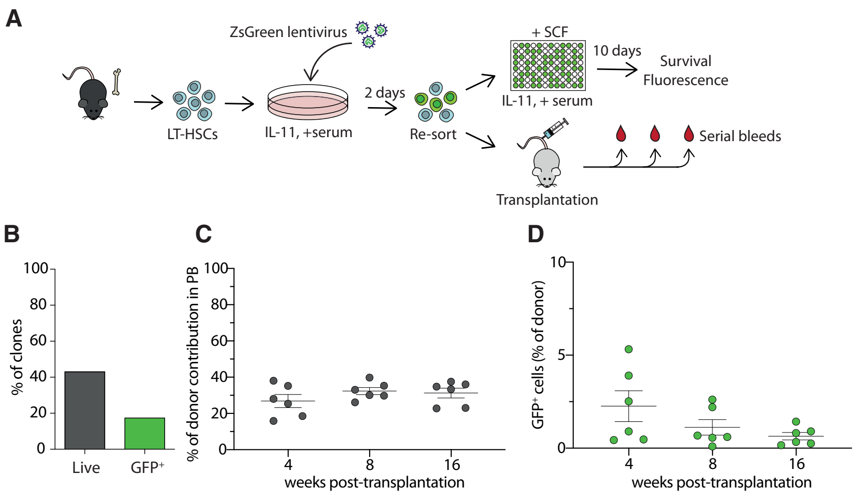

Figure 4. Single hibernating HSCs can be manipulated by lentiviral transduction

(A) $CD45^+EPCR^+CD48^-CD150^+$ (ESLAM) cells were isolated and transduced with ZsGreen lentivirus and cultured together for 2 days in StemSpan supplemented with 10% fetal calf serum and IL-11. Cells were collected and virus was removed by collecting and re-sorting the cells into single wells and cultured in SCF-supplemented media for additional 10 days. A total of 4,001 total viable cells (a mixture of transduced and non-transduced cells) were re-sorted and transplanted into W41-CD45.1 (n = 6 recipients), and donor contribution and GFP expression were assessed by serial bleeds and flow cytometry analysis.

(B) Graph shows the percentage of clones surviving after 10 days post-addition of SCF, and the green bar indicated the percentage of GFP^+ clones.

(C and D) Chimerism levels (20%–40%) were stable across all recipients at all time points (C), and an average of 1%–2% of donor cells was positive for GFP at 16 weeks post-transplantation (D). Bars show mean with SEM.

successfully repopulated recipients versus those that did not (Figure 3C). Interestingly, when we compared the cell division kinetics and 10-day colony size of single HSCs with high versus low expression of CD150, we observed smaller colonies from cells expressing high levels of CD150 (Figures 3D and 3E). Together these data suggest that, while higher CD150 expression can isolate cells enriched for resilient LT-HSCs with lower *in vitro* proliferation, the cells with lower CD150 expression that survive do not have compromised transplantation ability, which is supported by previous datasets examining CD150 expression in freshly isolated and transplanted HSCs (Beerman et al., 2010; Morita et al., 2010; Wilson et al., 2015).

Hibernating LT-HSCs can be transduced without undergoing division

To further explore the experimental and clinical potential of hibHSC culture conditions, we next assessed whether transgenes could be delivered during the hibernation period. Small bulk populations of LT-HSCs were isolated and exposed to a GFP-containing lentivirus for 2 days and then re-sorted into single-cell cultures to determine single-cell transduction efficiencies and survival (Figure 4A). After 10 days, 40% of the original sorted clones (284/657) successfully produced colonies, with ∼17.6% (50/284) of the surviving clones being GFP^+ (Figure 4B). In a second experiment to assess the *in vivo* functional potential of transduced hibernating LT-HSCs, bulk cells were transplanted following the 2-day transduction and assessed for GFP^+ donor cell repopulation at 4, 8, and 16 weeks post-transplantation (Figure 4A). All recipient mice were positive with initial reconstitution levels ranging from 2% to 6% GFP^+ cells (Figures 4C and 4D), and this contribution was stable throughout the monitoring period, although early time points appear slightly higher, suggesting that HSCs with less-durable self-renewal might be preferentially transduced. Together, these data demonstrate that lentiviral constructs can be successfully delivered to LT-HSCs in hibernation cultures without cell division.

Hibernating LT-HSCs share a core gene expression program with freshly isolated LT-HSCs

LT-HSCs deprived of SCF and TPO in hibernation conditions retain their functional properties, including the ability to reconstitute primary and secondary recipients (Figures 2B and 2C). Aside from IL-11, these LT-HSCs were cultured without signals from the hematopoietic niche or neighboring cells, making the transcriptome of these LT-HSCs a useful comparator for determining which genes might be dispensable for LT-HSC function. To address this question, we performed scRNA-seq on LT-HSCs cultured in serum-free hibernating conditions for 7 days (n = 106) and compared them to freshly isolated single LT-HSCs (n = 165) and also to LT-HSCs stimulated with SCF for 16 h (from both HSC + SCF [n = 63] and hibHSC + SCF [n = 127]) to determine the common pathways of activation upon SCF stimulation.

To determine broad differences between cell fractions, we performed dimensionality reduction using Uniform Manifold Approximation and Projection (UMAP) on single cells from all four conditions. Cells from each physiological setting clustered together in a unique space (Figures 5A and S1A). These data indicate that, while there is substantial similarity to the molecular profile of freshly isolated HSCs, there are some molecular changes that result from

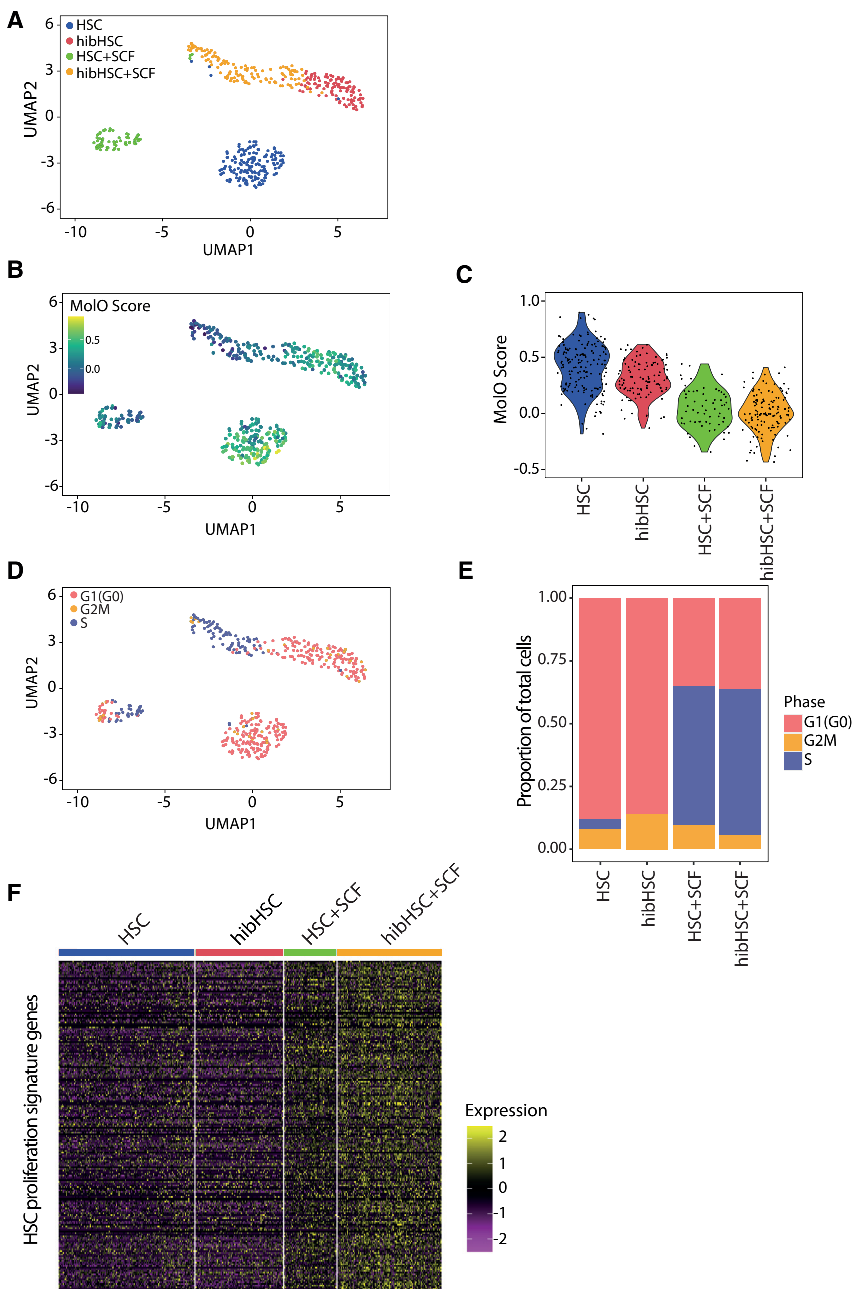

Figure 5. Gene expression profiling reveals a common transcriptional program between freshly isolated and hibernating HSCs

(A) Uniform Manifold Approximation and Projection (UMAP) of scRNA-seq profiles derived from four distinct populations (HSC, blue dots; hibHSC, red dots; HSC + SCF, green dots; hibHSC + SCF, orange dots).

(B) The HSC-specific Molecular Overlap (MolO) gene signature score was computed based on average expression of signature genes and projected onto the UMAP distribution.

(C) MolO scores for the individual HSCs in each physiological state with the HSCs and hibHSCs having the highest overall scores.

(D) Cell-cycle scores were computed for each cell and identified states were projected on the UMAP display from 5A (G1(G0), pink; G2/M, orange; S, blue).

(E) A proportional representation of cell-cycle stages of all cells within each distinct population (G1(G0), pink; G2/M, orange; S, blue).

(F) Heatmap of previously identified HSC-specific proliferation signature genes (Venezia et al., 2004) sorted by cell type with low expression in HSCs and hibHSCs and high expression in both sets of SCF-stimulated cells.

being removed from the *in vivo* microenvironment for 7 days.

To assess the similarity of hibHSCs to freshly isolated HSCs further, we compared the expression levels of key HSC regulators that comprise the previously reported Molecular Overlap (MolO) gene signature (Wilson et al., 2015). Overlaying MolO scores on the UMAP plot shows that the highest MolO scores are present in the freshly isolated HSCs, followed by the hibHSCs, and then their SCF-stimulated counterparts (Figure 5B). This pattern is mirrored in the violin plots displaying individual single-cell MolO scores by physiological condition (Figure 5C). Individual genes comprising the MolO score and their relative expression across the four biological states are provided in Figure S1B. The relatively high MolO scores in hibHSCs indicates the utility of the MolO score for identifying functional HSCs irrespective of their physiological state. The similarity in these molecular features also suggests that other factors must be contributing to the clear separation between freshly isolated HSCs and hibHSCs.

Another example of molecular similarity between hibHSCs and freshly isolated HSCs was evident when

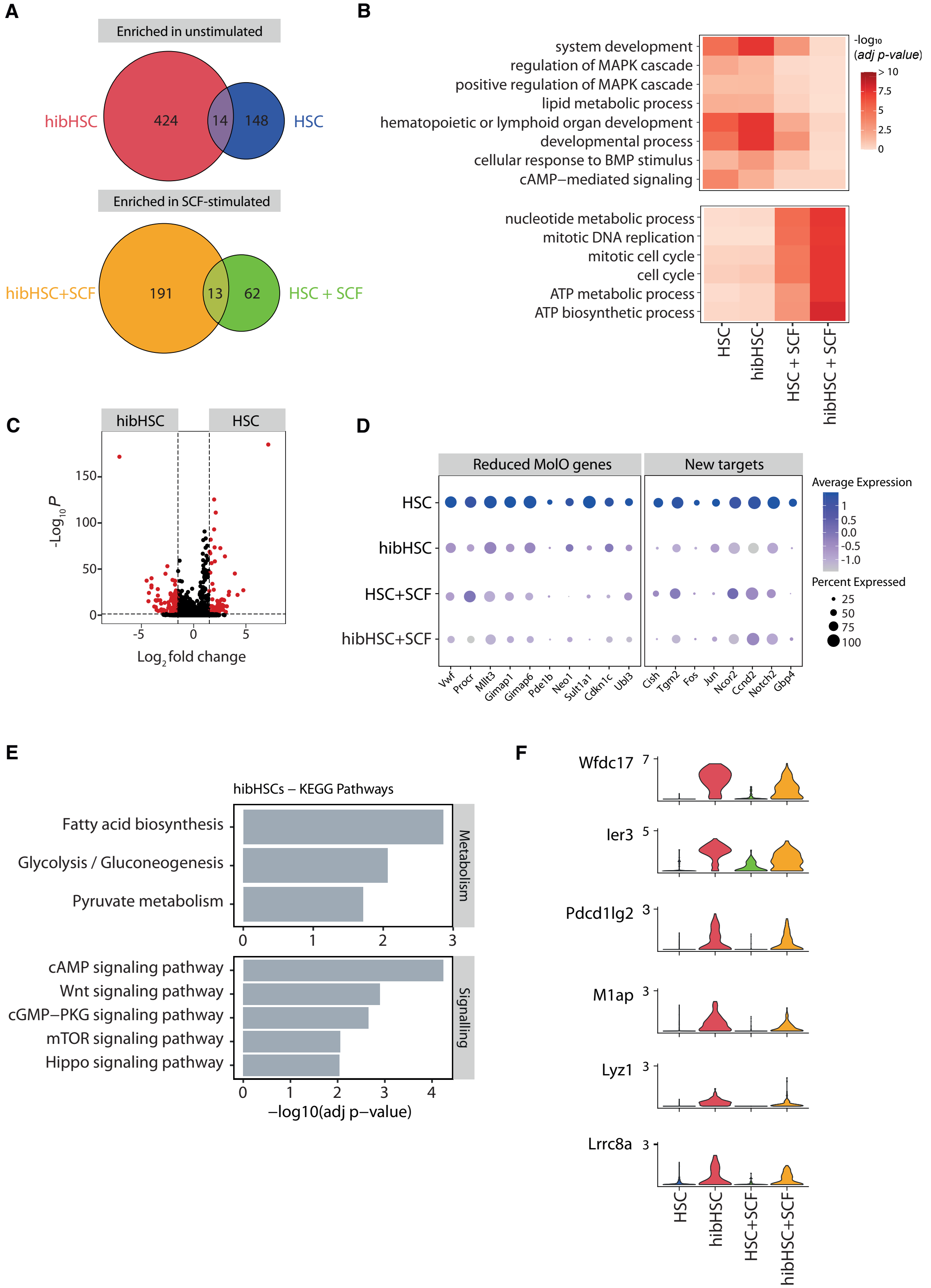

(legend on next page)

components of the cell-cycle machinery were assessed to predict the cell-cycle stage of each profiled LT-HSC (Nestorowa et al., 2016; Hamey and Göttgens, 2019). Again, UMAP clustering (Figure 5D) shows that cell-cycle status is not the primary driver of molecular differences between freshly isolated and hibHSCs, with the vast majority of cells in both cases being in the G_0/G_1 phase of the cell cycle (Figure S1C). Overall, more than 80% of freshly isolated HSCs and hibHSCs had molecular profiles consistent with being in the G_0/G_1 phase of the cell cycle (Figure 5E), whereas both SCF-stimulated HSC fractions had fewer than 40% G_0/G_1 cells. These data also accord with the cell-cycle kinetics observed in Figure 1D, where cells that divide early in the curve (i.e., between 20 and 30 h after stimulation) would be expected to have progressed to the S or G_2 phase by 16 h after stimulation. This is further emphasized by the heatmap in Figure 5E which displays the HSC proliferation gene signature from Venezia et al. (2004), where both freshly isolated and hibHSCs express low levels of proliferation-related genes (Figures 5F and S2A–S2E). Finally, we also assessed markers of autophagy and senescence and in neither case did we observe a significant enrichment (Figures S3A and S3B).

Hibernation cultures resolve common pathways of cytokine activation

Historically, the molecular impact of adding specific cytokines to HSCs has been performed following their direct isolation from the *in vivo* microenvironment. However, the impact that membrane dynamics, protein turnover, and transcriptional priming would have on the response of an HSC to a particular extracellular signal remains unclear. Hibernation cultures offer a different physiological state of highly purified HSCs from which to understand the direct impact of cytokine addition to a functional HSC. First, we observed the impact of culturing HSCs in IL-11 alone during the hibernation condition, allowing us to resolve the pathways activated or suppressed in response to IL-11 (Figures S3C–S3E). Next, using SCF as a stimulant, we profiled freshly isolated HSCs and hibHSCs to identify individual gene expression patterns associated with SCF stimulation (HSC + SCF, hibHSC + SCF). We first generated differentially expressed gene lists from the HSC versus HSC + SCF and hibHSC versus hibHSC + SCF (Figure 6A). Twenty-seven genes were commonly differentially expressed (13 up and 14 down) upon addition of SCF with an expected activation of ATP generation and nucleotide metabolism alongside a number of positive cell-cycle mediators (*Mcm2*, *Mcm4*, *Mcm10*, *Rad51*, and *Rad51ap1*) and a reduction in developmental and MAPK-mediated signaling (Figures 6B and S4A). In addition to these expected changes, we also identified SCF targets specifically induced in HSCs (Figure S4A) and show that expression of Mif (Ohta et al., 2012) (an inflammatory cytokine promoting survival and proliferation) and Txn1 (Schenk et al., 1994) (regulator of AP-1 signaling) are directly promoted upon SCF addition to functional HSCs.

Hibernating HSCs downregulate the AP1 complex and other stem cell regulators

Despite the strong overlap in cell-cycle and MolO gene signature expression, hibHSCs form a distinct cluster away from freshly isolated HSCs (Figures 5A and Figure S4B). While some of this distance could be attributable to downregulation of specific MolO genes (including *Sult1a1* and *Gimap1*, Figure 6D), global differential gene expression analysis between HSCs and hibHSCs identified 116 upregulated and 138 downregulated genes (Figure 6C). Among those additional genes whose expression was significantly reduced, a number of AP-1 complex members were identified, including *Jun* and *Fos* and their co-regulator *Ncor2* as well as molecules with previously described

Figure 6. Hibernating HSCs have a unique molecular profile of stress response

(A) Differential gene expression (DGE) was computed for two separate comparisons: (1) comparison of fresh HSCs (HSC) against SCF-stimulated HSCs (HSC + SCF); (2) comparison of hibernating HSC (hibHSCs) against hibHSCs post-SCF-stimulation (hibHSC + SCF) (negative binomial distribution, adjusted with Benjamini-Hochberg correction). Venn diagrams represent the number of genes commonly enriched in unstimulated populations (HSC and hibHSC) and SCF-stimulated populations (HSC + SCF and hibHSC + SCF) from both separate DGE computations.

(B) Gene ontology term enrichment was computed based on differentially expressed genes, as outlined in (A). Minimum p value > 0.05 to be considered significantly enriched.

(C) Volcano plot of differentially expressed genes (red dots), comparing fresh HSCs (HSC) and hibernating HSCs (hibHSC) (negative binomial distribution, adjusted with Benjamini-Hochberg correction).

(D) Dot plot representing the average normalized expression of genes across the four distinct populations. Genes of interest and MolO signature genes were selected from DGE in (C). The size of each dot indicates the proportion of cells with normalized expression level >0 (scaled expression represented by color intensity).

(E) KEGG pathway enrichment in unstimulated hibernating HSCs (hibHSC), showing selected metabolic and signal transduction pathways (enrichment cutoff: adjusted p value > 0.05).

(F) Violin plots of normalized gene expression of selected differentially expressed genes, enriched in unstimulated hibernating HSCs (hibHSCs).

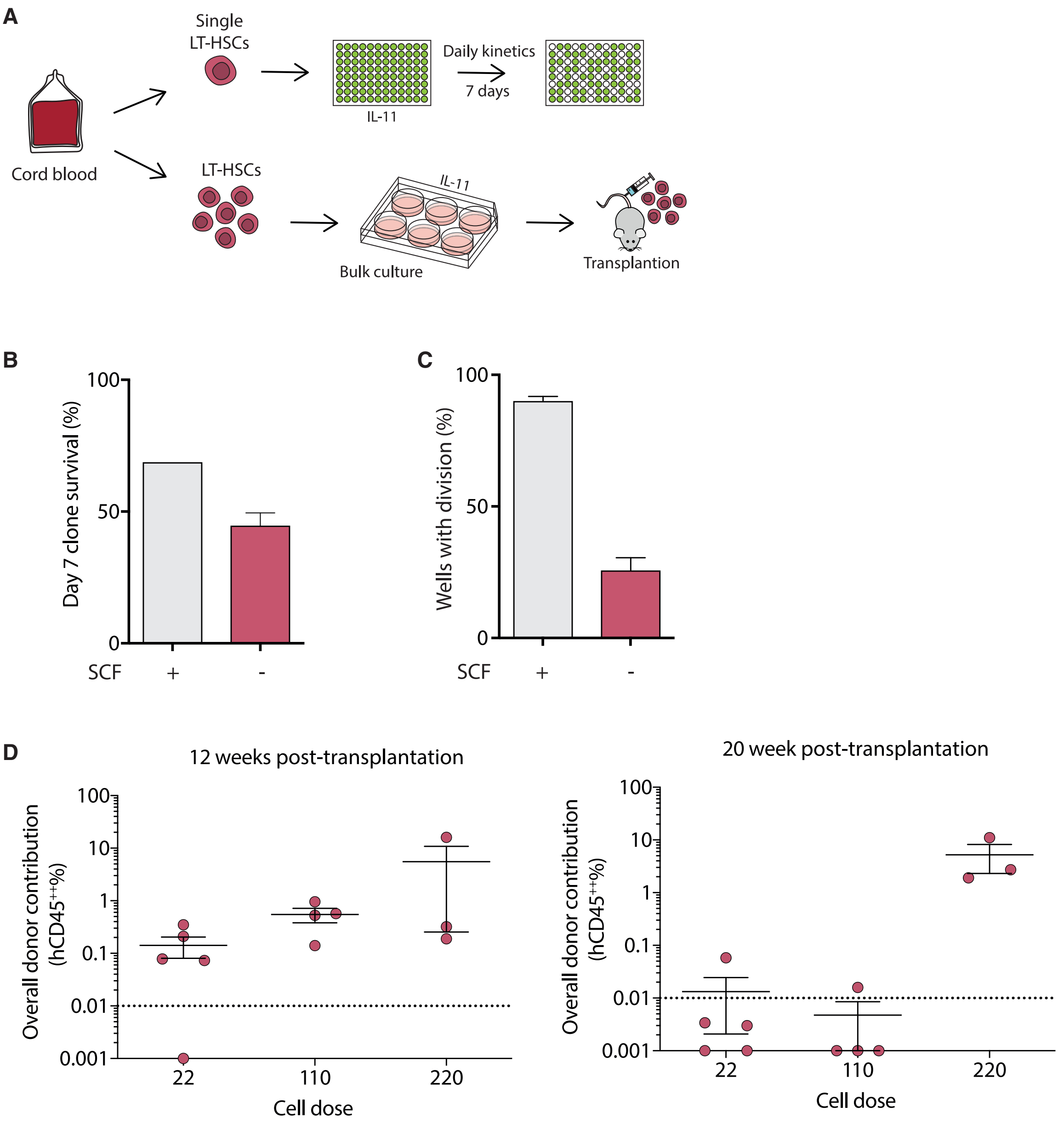

Figure 7. Hibernation conditions keep the majority of human HSCs as single cells

(A) Single human HSCs ($CD34^{+}CD38^{-}CD90^{+}CD45RA^{-}CD19^{-}CD49f^{+}$) from umbilical cord blood were sorted into individual wells and cultured in the presence of IL-11 with or without SCF. In parallel, human HSCs were bulk-cultured for 7 days in the absence of SCF and transplanted at three different cell doses (22, 110, and 218) into immunodeficient recipients and monitored for engraftment.

(B) Survival of HSCs in the presence or absence of SCF over 7 days, where absence results in 1.5-fold reduced survival compared with SCF-supplemented cultures (fresh n = 192; post-hibernation, n = 672; 5 independent experiments)

(C) The proportion of cells divided at 5–7 days in culture with and without the addition of SCF is displayed. Significantly more cells divide in the presence of SCF with the majority of cells in hibernation conditions remaining as single cells (fresh, three independent experiments, post-hibernation, five independent experiments). Bars show mean with SEM.

(D) The graphs show the percentage of human cell engraftment (%$CD45^{++}$) in PB from transplanted mice at 12 and 20 weeks post-transplantation (cell dose 22, n = 5; 110, n = 4; 218, n = 3). The threshold for events considered as positive was >0.01% with a minimum of 30 analyzed events. Non-engrafted mice shown below the dashed line. $CD45^{++}$ indicates cells positive for two distinct CD45 antibodies. Bars show mean with SEM.

roles in HSC biology, such as *Cish* (Schepers et al., 2012) and *Vwf* (Figures 6D and S4C). Since hibHSCs retain their functional properties *in vivo*, these data suggest that high levels of these genes are not a requirement for HSC function. On the other hand, pathways that were highly upregulated in hibHSCs were associated with stress response and nutrient deprivation, consistent with being kept in minimal cytokine conditions, and KEGG pathway analysis identified cAMP and mTOR signaling (Dhawan and Laxman, 2015) alongside glycolysis and fatty acid biosynthesis (Figure 6E). This accords with enrichment of HSC pro-survival genes *Ier3* and *Pdcd1lg2* expression in hibernating HSCs. Of additional interest, multiple HLF target genes, including *Lyz1* and *Lrrc8a*, were overexpressed in hibernated HSCs, potentially supporting the notion that HSCs are exerting a stress response to maintain survival/quiescence (Komorowska et al., 2017) in response to cytokine deprivation (Figures 6F and S5).

Human HSCs can be retained as single cells in hibernation conditions

To investigate whether cytokine deprivation had a similar effect on human HSCs (hHSCs), we isolated single human $CD34^{+}CD38^{-}CD90^{+}CD45RA^{-}CD19^{-}CD49f^{+}$ cells from cord blood and cultured them in serum-free medium with human IL-11 alone for 7 days (Figure 7A). Similar to mouse LT-HSCs, survival was lower with cytokine deprivation (Figure 7B) and, although some cells divided (~25.6%, Figure 7C), a large proportion remained as single cells compared with hHSCs under standard cytokine conditions (Belluschi et al., 2018; Ortmann et al., 2015). The fact that some hHSCs divided may be due to the starting purity or activation state of HSCs from cord blood. Upon transplantation of limited numbers of day 7 cultured hHSCs, repopulation was stable out to 20 weeks post-transplantation, but donor repopulation was below detection for the lowest-dose recipients (Figure 7D). Together these results demonstrate that IL-11 alone can maintain a proportion of multi-potent hHSCs in a non-dividing state, but further culture optimization would be required to support retention of large numbers of fully functional hHSCs.

DISCUSSION

Recent studies have produced a substantial amount of single-cell gene expression data from normal and malignant hematopoietic cells isolated from the mouse bone marrow (Shepherd and Kent, 2019). As a result, the transcriptional program of a quiescent "steady-state" LT-HSC is firmly established. Which genes drive individual LT-HSC properties (e.g., quiescence, self-renewal, differentiation, stress response) is much less well understood, and is complicated by only being able to obtain highly purified functional LT-HSCs from a single physiological state (i.e., quiescent cells from the bone marrow niche). Indeed, studies that have compared LT-HSCs to their downstream progenitors have identified "cell-cycle" changes as the dominant molecular feature separating LT-HSCs from non-HSCs (Passegué et al., 2005; Wilson et al., 2015). Hibernation cultures allow us to isolate and maintain functional LT-HSCs for prolonged periods of time in the absence of other cells without undergoing cell division or differentiation, thereby allowing the resolution of the common molecular program of HSCs in different physiological state. We identify molecules potentially dispensable for HSC function and a common molecular program of SCF activation in purified HSCs from distinct states. Finally, our study also resolves a debate about the impact of serum exposure on the cell fate of LT-HSCs (Domen and Weissman, 2000; Ieyasu et al., 2017; Rogers et al., 2008), showing that LT-HSCs can be cultured in the presence of serum for 7 days without undergoing differentiation or proliferation.

Distinct endogenous signaling pathways have been shown to regulate LT-HSC survival, self-renewal, and proliferation in both mouse (Wohrer et al., 2014) and human (Knapp et al., 2017). A similar cellular phenomenon of hibernation was observed when LT-HSCs were exposed to inhibitors that blocked lipid raft clustering (even in the presence of SCF) and remained undifferentiated as single cells for 5–7 days in culture (Yamazaki et al., 2006). Despite being deprived completely of TPO and SCF signaling, our hibernation cultures contain IL-11, without which all cells die within 48 h. One of the key pathways activated by IL-11 is gp130, which has been historically implicated in a wide array of stem cell systems, including mouse embryonic stem cells with LIF (Nichols et al., 2001), the *Drosophila* germ stem cell niche with Upd (Amoyel and Bach, 2012), mouse neural stem cells with CTNF and LIF (Shimazaki et al., 2001), mouse muscle stem cells with OSM (Sampath et al., 2018), and mouse HSCs with IL-6 and IL-11 (Yoshida et al., 1996; Audet et al., 2001). Of particular interest, OSM was shown to promote muscle cell engraftment without inducing proliferation (Sampath et al., 2018), lending additional support to the hypothesis that gp130 stimulants may regulate survival of quiescent stem cells in multiple stem cell systems.

Whereas other *in vitro* conditions have been shown to maintain mouse LT-HSCs, these systems uniformly create populations of cells in which LT-HSCs are the vast minority of the final culture (Bak et al., 2018; Gundry et al., 2016; Wagenblast et al., 2019; Wilkinson et al., 2019). In the absence of a robust *in vitro* LT-HSC purification strategy, molecular studies are therefore compromised by large numbers of contaminating non-HSCs. Our study

averts this issue by retaining functional LT-HSCs as single cells. The gene expression programs of single functional LT-HSCs in 7-day hibernation conditions show a high retention of known self-renewal regulators, and are consistent with the cells being in G_0/G_1. They also identify several regulators whose absence does not impact HSC engraftment or serially repopulation. One such set of factors was the AP1 complex, where expression of several members including *Jun*, *Fos*, and *Ncor2* was significantly reduced in hibernation cultures. This is potentially due to the hibernation cultures driving their extinguished expression and cells that do not have sufficient amounts of AP1 complex members do not survive. In contrast, i*n vivo* loss or reduced AP1 function leads to increased proliferation and differentiation (Santaguida et al., 2009). It may be that expression of these molecules is rescued upon transplantation when HSCs expand, although the SCF-induced entry into cell cycle does not on its own initiate their expression.

A previous study has reported that low cytokine concentration in culture facilitates the maintenance of engraftable mouse and hHSCs (Kobayashi et al., 2019) with reduced proliferation *in vitro* and this finding is supported by studies showing that slow-dividing LT-HSC clones were much more likely to retain HSC function (Dykstra et al., 2006; Laurenti et al., 2015). However, none of these studies were able to retain single LT-HSCs at high purities with indistinguishable properties from freshly isolated LT-HSCs, making it impossible to perform molecular studies on single functional HSCs or to manipulate them at the single-cell level. Hibernation cultures permit such analyses since single LT-HSCs do not lose any functional capacity with a highly similar, if not slightly improved, primary and secondary transplantation capacity compared with freshly isolated HSCs.

The finding that high CD150 expression levels prospectively identify resilient HSCs that survive hibernation are broadly consistent with data that implicate CD150 as a marker of LT-HSCs with more durable self-renewal capacity in serial transplantation assays (Beerman et al., 2010; Kent et al., 2009; Morita et al., 2010). The highest levels of CD150 also associated with a delayed engraftment in primary transplantations, an initial deficiency in making lymphoid cells (Kent et al., 2009; Morita et al., 2010), and an ability to create daughter HSCs with full multi-lineage potential (Dykstra et al., 2007; Komorowska et al., 2017). This further accords with the increased number of α-HSCs (myeloid-biased) observed in our transplantation data. The delay in engraftment observed generally in α-HSCs may be related to the dynamics of quiescence/activation of daughter LT-HSCs in a transplantation scenario and our *in vitro* hibernation system offers the chance to study HSC activation in a distinct physiological context with unprecedented resolution. This latter capacity is particularly important in the context of HSC transplantation where cells need to exit, and eventually return to, quiescence during any sort of *in vitro* culture period and subsequent reseeding of recipient bone marrow.

Optimization of hibernation cultures for manipulating highly purified LT-HSCs would also have a wide range of applications in experimental and clinical research. The knowledge that LT-HSCs are fully functional during hibernation offers the opportunity to manipulate them at the single-cell level with precise assessment of the impact of specific modifications. Our data show that genetic modification can be undertaken in hibernation cultures which could potentially set the stage for the delivery of multiple viral constructs during the culture period. This would permit studies of combinatorial genetic modifications in highly purified LT-HSCs, as opposed to a heterogeneous pool of stem and progenitor cells typically assayed in such protocols. Finally, we provide proof-of-principle evidence that hibernation cultures can be adapted to the human setting, offering substantial potential for implementing genetic modifications in hHSCs and setting the stage for more precise interrogation of the functional properties of individual LT-HSCs.

EXPERIMENTAL PROCEDURES

Mice

C57BL/6-Ly5.2 (wild type) were purchased from Charles River (Saffron Walden, Essex, UK). C57BL/6w41/w41-Ly5.1 (W41) were bred and maintained at the University of Cambridge. Full details are available in the supplemental information.

Isolation of mouse Sca1^{high} ESLAM HSCs, *in vitro* assays, and expression profiling

HSCs were isolated from the lineage-depleted cell suspension by using fluorescence-activated cell sorting using EPCR^{high}, CD45^{+}, Sca-1^{high}, CD48$^{low/neg}$, and CD150^{+} (or ESLAM), as described previously (Kent et al., 2009) with full details found in the supplemental information.

Bone marrow transplantation assays and analysis

Donor cells were obtained from C56BL/6J mice (CD45.2). Recipient mice were C57Bl6W41/W41 (W41) mice as described previously (Benz et al., 2012; Kent et al., 2009).

Full details of transplantation and peripheral blood analysis are in the supplemental information.

Lentiviral transduction of mouse HSCs

ESLAM HSCs (7,000 cells) were isolated and transduced with GFP-containing lentivirus; full details of the transduction method and assays are in the supplemental information.

Isolation of human cord blood HSCs and *in vitro* assays

Cord blood samples were obtained from the Cambridge Blood and Stem Cell Biobank with informed consent from healthy donors in accordance with regulated procedures approved by the relevant Research and Ethics Committees. Details of HSC isolation and *in vitro* assays are given in the supplemental information.

scRNA-seq

scRNA-seq analysis was performed as described previously in Picelli et al., 2014) (Smart-seq2), with full details given in the supplemental information. Data are publicly available using the GEO accession number: GSE160131. All code is available upon request.

Xenotransplantation and analysis

Donor cells were obtained from CD34-enriched cord blood samples. Recipient mice were NSG. Full details of transplantation and peripheral blood analysis are given in the supplemental information.

SUPPLEMENTAL INFORMATION

Supplemental information can be found online at https://doi.org/10.1016/j.stemcr.2021.04.002.

AUTHOR CONTRIBUTIONS

C.A.O., M.B., D.G.K., and E.L. conceived and designed the experiments. C.A.O., M.B., M.S.S., J.L.C.C., G.B., C.McD., and S.B. performed the experiments. C.A.O., M.B., D.B., F.K.H., E.D., and H.P.B. analyzed the data. M.B., D.B., and D.G.K. wrote the paper with input from E.L. and B.G.

DECLARATION OF INTERESTS

The authors declare no competing interests.

ACKNOWLEDGMENTS

We thank Reiner Schulte, Chiara Cossetti, and Gabriela Grondys-Kotarba of the CIMR Flow Cytometry core and Anna Petrunkina-Harrison in the NIHR BRC Cell Phenotyping Hub for technical assistance and suggestions; Tina Hamilton, Dean Pask, Nicole Mende, Emily Calderbank, Carys Johnson, Rebecca Hannah, and Winnie Lau for technical assistance; Sally Thomas and the Central Biomedical Services unit staff; Joanna Baxter and the Cambridge Blood and Stem Cell Biobank staff; and Nicola Wilson, Fernando Calero-Nieto, Robert Oostendorp, and Emmanuelle Passegué for helpful discussion. C.A.O. and D.B. were supported by Wellcome PhD Studentships, F.K.H. was supported by a Medical Research Council (MRC) PhD Studentship, M.S.S. was the recipient of a Biotechnology and Biological Sciences Research Council (BBSRC) Industrial Collaborative Award in Science and Engineering PhD Studentship, S.B. was supported by a CRUK Cambridge Cancer Centre PhD Studentship, and J.L.C.C. was supported by an MRC PhD Studentship under the University of Cambridge Doctoral Training Programme. Research in the B.G. lab is supported by Wellcome, Blood Cancer UK, and an MRC-AMED joint award (MR/V005502/1). E.L. is supported by a Wellcome/Royal Society Sir Henry Dale Fellowship (107630/Z/15/Z) and work in her laboratory is also supported by the European Hematology Association and BBSRC (BB/P002293/1). The D.G.K. laboratory is supported by a Blood Cancer UK Bennett Fellowship (15008), an ERC Starting Grant (ERC-2016-STG–715371), and an MRC-AMED joint award (MR/V005502/1). D.G.K., E.L., and B.G. are supported by a core support grant to the Wellcome MRC Cambridge Stem Cell Institute, Blood Cancer UK, the NIHR Cambridge Biomedical Research Centre, and the CRUK Cambridge Cancer Centre.

Received: March 8, 2021
Revised: April 7, 2021
Accepted: April 7, 2021
Published: May 6, 2021

REFERENCES

Amoyel, M., and Bach, E. (2012). Functions of the *Drosophila* JAK-STAT pathway: lessons from stem cells. JAK-STAT. https://doi.org/10.4161/jkst.21621.

Audet, J., Miller, C.L., Rose-John, S., Piret, J.M., and Eaves, C.J. (2001). Distinct role of gp130 activation in promoting self-renewal divisions by mitogenically stimulated murine hematopoietic stem cells. Proc. Natl. Acad. Sci. U S A *98*, 1757–1762. https://doi.org/10.1073/pnas.98.4.1757.

Bak, R.O., Dever, D.P., and Porteus, M.H. (2018). CRISPR/Cas9 genome editing in human hematopoietic stem cells. Nat. Protoc. https://doi.org/10.1038/nprot.2017.143.

Beerman, I., Bhattacharya, D., Zandi, S., Sigvardsson, M., Weissman, I.L., Bryder, D., and Rossi, D.J. (2010). Functionally distinct hematopoietic stem cells modulate hematopoietic lineage potential during aging by a mechanism of clonal expansion. Proc. Natl. Acad. Sci. U S A *107*, 5465–5470. https://doi.org/10.1073/pnas.1000834107.

Belluschi, S., Calderbank, E.F., Ciaurro, V., Pijuan-Sala, B., Santoro, A., Mende, N., Diamanti, E., Sham, K.Y.C., Wang, X., Lau, W.W.Y., et al. (2018). Myelo-lymphoid lineage restriction occurs in the human haematopoietic stem cell compartment before lymphoid-primed multipotent progenitors. Nat. Commun. *9*, 1–15. https://doi.org/10.1038/s41467-018-06442-4.

Benz, C., Copley, M.R., Kent, D.G., Wohrer, S., Cortes, A., Aghaeepour, N., Ma, E., Mader, H., Rowe, K., Day, C., et al. (2012). Hematopoietic stem cell subtypes expand differentially during development and display distinct lymphopoietic programs. *Cell Stem Cell* *10*, 273–283. https://doi.org/10.1016/j.stem.2012.02.007.

Dhawan, J., and Laxman, S. (2015). Decoding the stem cell quiescence cycle—lessons from yeast for regenerative biology. J. Cell Sci. *128*, 4467–4474. https://doi.org/10.1242/jcs.177758.

Domen, J., and Weissman, I.L. (2000). Hematopoietic stem cells need two signals to prevent apoptosis; BCL-2 can provide one of these, Kitl/c-Kit signaling the other. J. Exp. Med. https://doi.org/10.1084/jem.192.12.1707.

Doulatov, S., Notta, F., Laurenti, E., and Dick, J.E. (2012). Hematopoiesis: a human perspective. Cell Stem Cell, 120–136. https://doi.org/10.1016/j.stem.2012.01.006.

Dykstra, B., Ramunas, J., Kent, D., McCaffrey, L., Szumsky, E., Kelly, L., Farn, K., Blaylock, A., Eaves, C., and Jervis, E. (2006). High-

resolution video monitoring of hematopoietic stem cells cultured in single-cell arrays identifies new features of self-renewal. Proc. Natl. Acad. Sci. U S A. https://doi.org/10.1073/pnas.0602548103.

Dykstra, B., Kent, D., Bowie, M., McCaffrey, L., Hamilton, M., Lyons, K., Lee, S.J., Brinkman, R., and Eaves, C. (2007). Long-term propagation of distinct hematopoietic differentiation programs in vivo. Cell Stem Cell *1*, 218–229. https://doi.org/10.1016/j.stem.2007.05.015.

Eaves, C.J. (2015). Hematopoietic stem cells: concepts, definitions, and the new reality. Blood *125*, 2605–2613. https://doi.org/10.1182/blood-2014-12-570200.

Fares, I., Chagraoui, J., Lehnertz, B., MacRae, T., Mayotte, N., Tomellini, E., Aubert, L., Roux, P.P., and Sauvageau, G. (2017). EPCR expression marks UM171-expanded CD34+ cord blood stem cells. Blood *129*, 3344–3351. https://doi.org/10.1182/blood-2016-11-750729.

Ganuza, M., Hall, T., Obeng, E.A., and McKinney-Freeman, S. (2020). Clones assemble! the clonal complexity of blood during ontogeny and disease. Exp. Hematol. *83*, 35–47. https://doi.org/10.1016/j.exphem.2020.01.009.

De Graaf, C.A., and Metcalf, D. (2011). 'Thrombopoietin and Hematopoietic Stem Cells', *Cell Cycle* (Taylor and Francis Inc.), pp. 1582–1589. https://doi.org/10.4161/cc.10.10.15619.

Gundry, M.C., Brunetti, L., Lin, A., Mayle, A.E., Kitano, A., Wagner, D., Hsu, J.I., Hoegenauer, K.A., Rooney, C.M., Goodell, M.A., and Nakada, D. (2016). Highly Efficient Genome Editing of Murine and Human Hematopoietic Progenitor Cells by CRISPR/Cas9. Cell Rep. https://doi.org/10.1016/j.celrep.2016.09.092.

Hamey, F.K., and Göttgens, B. (2019). Machine learning predicts putative hematopoietic stem cells within large single-cell transcriptomics data sets. Exp. Hematol. *78*, 11–20. https://doi.org/10.1016/j.exphem.2019.08.009.

Ieyasu, A., Ishida, R., Kimura, T., Morita, M., Wilkinson, A.C., Sudo, K., Nishimura, T., Ohehara, J., Tajima, Y., Lai, C.Y., et al. (2017). An all-recombinant protein-based culture system specifically identifies hematopoietic stem cell maintenance factors. Stem Cell Reports. https://doi.org/10.1016/j.stemcr.2017.01.015.

Kent, D., Copley, M., Benz, C., Dykstra, B., Bowie, M., and Eaves, C. (2008a). 'Regulation of hematopoietic stem cells by the steel factor/KIT signaling pathway. Clin. Cancer Res., 1926–1930. https://doi.org/10.1158/1078-0432.CCR-07-5134.

Kent, D.G., Dykstra, B.J., Cheyne, J., Ma, E., and Eaves, C.J. (2008b). Steel factor coordinately regulates the molecular signature and biologic function of hematopoietic stem cells. Blood *112*, 560–567. https://doi.org/10.1182/blood-2007-10-117820.

Kent, D.G., Copley, M.R., Benz, C., Wöhrer, S., Dykstra, B.J., Ma, E., Cheyne, J., Zhao, Y., Bowie, M.B., Zhao, Y., et al. (2009). Prospective isolation and molecular characterization of hematopoietic stem cells with durable self-renewal potential. Blood *113*, 6342–6350. https://doi.org/10.1182/blood-2008-12-192054.

Kent, D.G., Li, J., Tanna, H., Fink, J., Kirschner, K., Pask, D.C., Silber, Y., Hamilton, T.L., Sneade, R., Simons, B.D., and Green, A.R. (2013). Self-renewal of single mouse hematopoietic stem cells is reduced by JAK2V617F without compromising progenitor cell expansion. PLoS Biol. *11*, e1001576. https://doi.org/10.1371/journal.pbio.1001576.

Knapp, D.J.H.F., Hammond, C.A., Miller, P.H., Rabu, G.M., Beer, P.A., Ricicova, M., Lecault, V., Da Costa, D., VanInsberghe, M., Cheung, A.M., et al. (2017). Dissociation of survival, proliferation, and state control in human hematopoietic stem cells. Stem Cell Reports. https://doi.org/10.1016/j.stemcr.2016.12.003.

Kobayashi, H., Morikawa, T., Okinaga, A., Hamano, F., Hashidate-Yoshida, T., Watanuki, S., Hishikawa, D., Shindou, H., Arai, F., Kabe, Y., et al. (2019). Environmental optimization enables maintenance of quiescent hematopoietic stem cells ex vivo. Cell Rep. *28*, 145–158.e9. https://doi.org/10.1016/j.celrep.2019.06.008.

Komorowska, K., Doyle, A., Wahlestedt, M., Subramaniam, A., Debnath, S., Chen, J., Soneji, S., Van Handel, B., Mikkola, H.K.A., Miharada, K., et al. (2017). Hepatic leukemia factor maintains quiescence of hematopoietic stem cells and protects the stem cell pool during regeneration. Cell Rep. *21*, 3514–3523. https://doi.org/10.1016/j.celrep.2017.11.084.

Laurenti, E., Frelin, C., Xie, S., Ferrari, R., Dunant, C.F., Zandi, S., Neumann, A., Plumb, I., Doulatov, S., Chen, J., et al. (2015). CDK6 levels regulate quiescence exit in human hematopoietic stem cells. Cell Stem Cell. https://doi.org/10.1016/j.stem.2015.01.017.

Laurenti, E., and Göttgens, B. (2018). From haematopoietic stem cells to complex differentiation landscapes. Nature, 418–426. https://doi.org/10.1038/nature25022.

Morita, Y., Ema, H., and Nakauchi, H. (2010). Heterogeneity and hierarchy within the most primitive hematopoietic stem cell compartment. J. Exp. Med. *207*, 1173–1182. https://doi.org/10.1084/jem.20091318.

Nestorowa, S., Hamey, F.K., Pijuan Sala, B., Diamanti, E., Shepherd, M., Laurenti, E., Wilson, N.K., Kent, D.G., and Göttgens, B. (2016). A single-cell resolution map of mouse hematopoietic stem and progenitor cell differentiation. Blood *128*, e20–e31. https://doi.org/10.1182/blood-2016-05-716480.

Nichols, J., Chambers, I., Taga, T., and Smith, A. (2001). Physiological rationale for responsiveness of mouse embryonic stem cells to gp130 cytokines. Development.

Ohta, S., Misawa, A., Fukaya, R., Inoue, S., Kanemura, Y., Okano, H., Kawakami, Y., and Toda, M. (2012). Macrophage migration inhibitory factor (MIF) promotes cell survival and proliferation of neural stem/progenitor cells. Development *139*. https://doi.org/10.1242/jcs.102210.

Ortmann, C.A., Kent, D.G., Nangalia, J., Silber, Y., Wedge, D.C., Grinfeld, J., Baxter, E.J., Massie, C.E., Papaemmanuil, E., Menon, S., et al. (2015). Effect of mutation order on myeloproliferative neoplasms. New Engl. J. Med. *372*, 601–612. https://doi.org/10.1056/NEJMoa1412098.

Passegué, E., Wagers, A.J., Giuriato, S., Anderson, W.C., and Weissman, I.L. (2005). Global analysis of proliferation and cell cycle gene expression in the regulation of hematopoietic stem and progenitor cell fates. J. Exp. Med. *202*, 1599–1611. https://doi.org/10.1084/jem.20050967.

Picelli, S., Faridani, O.R., Björklund, A.K., Winberg, G., Sagasser, S., and Sandberg, R. (2014). Full-length RNA-seq from single cells

using Smart-seq2. Nat Protoc. *9*, 171–181. https://doi.org/10.1038/nprot.2014.006.

Rogers, I.M., Yamanaka, N., and Casper, R.F. (2008). A simplified procedure for hematopoietic stem cell amplification using a serum-free, feeder cell-free culture system. Biol. Blood Marrow Transplant. https://doi.org/10.1016/j.bbmt.2008.06.002.

Sampath, S.C., Sampath, S.C., Ho, A.T.V., Corbel, S.Y., Millstone, J.D., Lamb, J., Walker, J., Kinzel, B., Schmedt, C., and Blau, H.M. (2018). Induction of muscle stem cell quiescence by the secreted niche factor Oncostatin M. Nat. Commun. https://doi.org/10.1038/s41467-018-03876-8.

Santaguida, M., Schepers, K., King, B., Sabnis, A.J., Forsberg, E.C., Attema, J.L., Braun, B.S., and Passegué, E. (2009). JunB protects against myeloid malignancies by limiting hematopoietic stem cell proliferation and differentiation without affecting self-renewal. Cancer Cell *15*, 341–352. https://doi.org/10.1016/j.ccr.2009.02.016.

Schenk, H., Klein, M., Erdbrügger, W., Dröge, W., and Schulze-Osthoff, K. (1994). Distinct effects of thioredoxin and antioxidants on the activation of transcription factors NF-κB and AP-1. Proc. Natl. Acad. Sci. U S A *91*, 1672–1676. https://doi.org/10.1073/pnas.91.5.1672.

Schepers, H., Wierenga, A.T., Vellenga, E., and Schuringa, J.J. (2012). STAT5-mediated self-renewal of normal hematopoietic and leukemic stem cells. JAK-STAT *1*, 13–25. https://doi.org/10.4161/jkst.19316.

Shepherd, M.S., Li, J., Wilson, N.K., Oedekoven, C.A., Li, J., Belmonte, M., Fink, J., Prick, J.C.M., Pask, D.C., Hamilton, T.L., et al. (2018). Single-cell approaches identify the molecular network driving malignant hematopoietic stem cell self-renewal. Blood *132*. https://doi.org/10.1182/blood-2017-12-821066.

Shepherd, M.S., and Kent, D.G. (2019). Emerging single-cell tools are primed to reveal functional and molecular heterogeneity in malignant hematopoietic stem cells. Curr. Opin. Hematol. *26*, 214–221. https://doi.org/10.1097/MOH.0000000000000512.

Shimazaki, T., Shingo, T., and Weiss, S. (2001). The ciliary neurotrophic factor/leukemia inhibitory factor/gp130 receptor complex operates in the maintenance of mammalian forebrain neural stem cells. J. Neurosci. https://doi.org/10.1523/jneurosci.21-19-07642.2001.

Venezia, T.A., Merchant, A.A., Ramos, C.A., Whitehouse, N.L., Young, A.S., Shaw, C.A., and Goodell, M.A. (2004). 'Molecular signatures of proliferation and quiescence in hematopoietic stem cells. PLoS Biol. *2*, e301. https://doi.org/10.1371/journal.pbio.0020301.

Wagenblast, E., Azkanaz, M., Smith, S.A., Shakib, L., McLeod, J.L., Krivdova, G., Araújo, J., Shultz, L.D., Gan, O.I., Dick, J.E., and Lechman, E.R. (2019). Functional profiling of single CRISPR/Cas9-edited human long-term hematopoietic stem cells. Nat. Commun. https://doi.org/10.1038/s41467-019-12726-0.

Wilkinson, A.C., Ishida, R., Kikuchi, M., Sudo, K., Morita, M., Crisostomo, R.V., Yamamoto, R., Loh, K.M., Nakamura, Y., Watanabe, M., et al. (2019). Long-term ex vivo haematopoietic-stem-cell expansion allows nonconditioned transplantation. Nature *571*, 117–121. https://doi.org/10.1038/s41586-019-1244-x.

Wilson, N.K., Kent, D.G., Buettner, F., Shehata, M., Macaulay, I.C., Calero-Nieto, F.J., Sánchez Castillo, M., Oedekoven, C.A., Diamanti, E., Schulte, R., et al. (2015). Combined single-cell functional and gene expression analysis resolves heterogeneity within stem cell populations. Cell Stem Cell *16*, 712–724. https://doi.org/10.1016/j.stem.2015.04.004.

Wohrer, S., Knapp, D.J., Copley, M.R., Benz, C., Kent, D.G., Rowe, K., Babovic, S., Mader, H., Oostendorp, R.A., and Eaves, C.J. (2014). Distinct stromal cell factor combinations can separately control hematopoietic stem cell survival, proliferation, and self-renewal. Cell Rep. *7*, 1956–1967. https://doi.org/10.1016/j.celrep.2014.05.014.

Yamazaki, S., Iwama, A., Takayanagi, S., Morita, Y., Eto, K., Ema, H., and Nakauchi, H. (2006). Cytokine signals modulated via lipid rafts mimic niche signals and induce hibernation in hematopoietic stem cells. EMBO J. *25*, 3515–3523. https://doi.org/10.1038/sj.emboj.7601236.

Yamazaki, S., Iwama, A., Takayanagi, S., Eto, K., Ema, H., and Nakauchi, H. (2009). TGF- 2 as a candidate bone marrow niche signal to induce hematopoietic stem cell hibernation. Blood *113*, 1250–1256. https://doi.org/10.1182/blood-2008-04-146480.

Yoshida, K., Taga, T., Saito, M., Suematsu, S., Kumanogoh, A., Tanaka, T., Fujiwara, H., Hirata, M., Yamagami, T., Nakahata, T., et al. (1996). Targeted disruption of gp130, a common signal transducer for the interleukin 6 family of cytokines, leads to myocardial and hematological disorders. Proc. Natl. Acad. Sci. U S A *93*, 407–411. https://doi.org/10.1073/pnas.93.1.407.

Stem Cell Reports, Volume *16*

Supplemental Information

Hematopoietic stem cells retain functional potential and molecular identity in hibernation cultures

Caroline A. Oedekoven, Miriam Belmonte, Daniel Bode, Fiona K. Hamey, Mairi S. Shepherd, James Lok Chi Che, Grace Boyd, Craig McDonald, Serena Belluschi, Evangelia Diamanti, Hugo P. Bastos, Katherine S. Bridge, Berthold Göttgens, Elisa Laurenti, and David G. Kent

Supplementary Data Items

Mice

C57BL/6-Ly5.2 (WT) were purchased from Charles River Laboratory (Saffron Walden, Essex, UK). C57BL/6w41/w41-Ly5.1 (W41) were bred and maintained at the University of Cambridge. *NOD.Cg-Prkdc^{scid}Il2rg^{tm1Wjl}/SzJ* (NSG) mice were obtained from Charles River or bred in-house. Mice were maintained in the Central Biomedical Service (CBS) animal facility of Cambridge University and housed in specific pathogen-free environment, according to institutional guidelines.All the procedures performed were in compliance with the guidance on the operation of ASPA (Animals Scientific Procedures Act 1986), following ethical review by the University of Cambridge Animal Welfare and Ethical Review Body (AWERB).

Isolation of mouse Sca1^{high} ESLAM HSCs and *in vitro* assays

Bone marrow cells were isolated from spine, sternum, femora, tibiae and pelvic bones of both hind legs of WT mice. Bones were crushed in 2% Fetal Calf Serum (FCS, STEMCELL or Sigma Aldrich (Sigma)) and 1mM EDTA (Sigma) in PBS (Sigma). Red cell lysis was performed by treatment with Ammonium Chloride (NH_4Cl, STEMCELL). Depletion of mature lineage cells was performed using EasySep mouse hematopoietic progenitor cell enrichment kit (STEMCELL). HSCs were isolated from the lineage depleted cell suspension by using fluorescence-activated cell sorting (FACS) using EPCR^{high}, CD45^{+}, Sca-1^{high}, CD48$^{low/neg}$, CD150^{+} (or “ESLAM”), as described previously (Kent *et al.*, 2009), using CD45 FITC (Clone 30-F1,1 BD Biosciences, San Jose CA, USA (BD)), EPCR PE (Clone RMEPCR1560, STEMCELL), CD150 Pacific Blue (PB) or PE-Cy7 (Clone TC15-12F12.2, both from Biolegend, San Diego, USA (Biolegend)), CD48 APC (Clone HM48-1, Biolegend), Sca-1 Brilliant Violet (BV) 421 (Clone D7, Biolegend) and 7-Aminoactinomycin D (7AAD) (Life Technologies, Carlsbad, CA, USA (Life Technologies)). The cells were sorted in either purity or single sort mode on an Influx cell sorter (BD Biosciences, San Jose, CA, USA (BD)) using the following filter sets 488 530/40 (for FITC), 561 585/29 (for PE), 405 460/50 (for BV421), 640 670/30 (for APC), 561 750LP (for PE/Cy7), 640 750LP (for APC/Cy7), 405 520/35 (for BV510), 640 720/40 (for AF700), and 561 670/30 (for 7-AAD) or 405 450/50 (for DAPI). When single HSCs were required, the single-cell deposition unit of the sorter was used to place 1 cell into each well of a round bottom 96-well plate, each

well having been preloaded with 50uL medium which would be topped up with 50uL medium with 2X cytokines.

Normalisation of single cell index-sorting data

Surface marker intensity of single ESLAM HSCs across experiments were normalised and batch corrected by using the flowCore (version 1.42.3) and sva (version 3.24.4) R packages. HSCs were sorted in 96-well format and each plate was considered as an independent batch prior to batch correction. All recorded surface markers were arranged in a flow frame and subject to logicle transformation prior to batch correction. The analysis was computed in R (version 3.4.2) and performed by Daniel Bode. The original script was developed by Blanca Pijuan Sala.

Liquid cultures and clone size determination of mouse HSCs

Single HSCs were sorted and cultured into 100µL StemSpan SFEM (STEMCELL) supplemented with 300 ng/mL SCF (R&D Systems, Bio-Techne, Minneapolis, MI, USA, (R&D)), 20ng/mL human Interleukin-11 (IL-11, R&D), 2 mM L-Glutamine (Sigma), 1000 U/mL-100 µg/mL Penicillin-Streptomycin (Sigma), 100µM 2-Mercaptoethanol (Life Technologies). SCF concentration was 300ng/mL unless stated otherwise. 10% of FCS was supplemented when stated. For serum-free cultures, cells were sorted into Ham's F12 nutrient mixture (Gibco, ThermoFisher, Waltham, MA, USA (Gibco)) supplemented with 20 ng/mL human IL-11 (R&D), 300 ng/mL SCF (SCT or R&D), 2 mM L-Glutamine (Sigma), 1000U/mL-100 µg/mL Penicillin-Streptomycin (Sigma), 1% ITS-X (Insulin-Transferrin Selenium-Ethanolamine, Gibco), 100 mM HEPES (4-(2-hydroxyethyl)-1 piperazineethanesulfonic acid, Sigma), 100 mg/mL human serum albumin (HSA, Albumin Bioscience, Huntsville, AL, USA).

Cells were cultured at 37°C, 5% CO_2, 20% O_2. Cell counts were performed every 22-24 hours and cell cycle kinetics determined for the first and second division by visual inspection, scoring wells as having 1, 2, or 3-4 cells. Clone size at day 10 post-isolation was scored as very small (less than 50 cells), small (50-500 cells), medium (500-10,000 cells), or large (10,000 or more cells).

Time lapse of single mouse HSCs

Single cells were sorted into a 96 well plate and imaged on a Leica DMI3000 B microscope, housed inside an Okolab CO2 microscope cage incubator system. Custom written LabVIEW software was used to control a Prior Proscan III nanopositioning stage and acquire images via a Hamamatsu Orca Flash 4.0 camera. Cells were imaged every 50 minutes for the first 7 days, the fastest time resolution achievable with the system while allowing enough time for the autofocus routine to correctly execute at all 96 wells. On day 7, the plate was removed and 300ng/mL SCF was added to the 67 wells where there was a possibility of a viable cell, determined by eye. The reduction in well number allowed for an increase in time resolution to 35 minutes. By day 11, imaged well number was further reduced to 17 wells as it became more apparent in which wells cells were still viable. This allowed for a corresponding increase in time resolution to 20 minutes. Imaging continued until day 14.

Colony-forming assays of mouse HSCs

Single cultured cells (hibernated HSCs) were transferred from liquid culture into 600 µl of MethoCult GF M3434 (STEMCELL). Freshly isolated HSCs were isolated by FACS sorting (as described above) and plated into 3 mL Methocult GF M3434 (STEMCELL) and split across 2 wells of 6-well plates. Cells were cultured for 14 days and colony number was assessed by visual inspection and colony type scored by antibody staining with CD41 FITC (Clone MWReg30), CD61 PE (Clone 2C9.G2 (HMβ3-1), Ter119 PE-Cy7 (Clone TER-119), CD45.2 APC-Cy7 (Clone 104), Ly6G/Gr1 BV421 (Clone 1A8), CD11b/Mac1 APC (Clone M1/70). Samples were acquired on LSR Fortessa (BD) and flow cytometry data analysing by using FlowJo (Treestar, Ashland, OR, USA).

Bone Marrow Transplantation Assay and Peripheral Blood Analysis

Donor cells were obtained from C56BL/6J mice (CD45.2). Recipient mice were C57Bl6W41/W41 (W41) mice as described previously (Kent *et al.*, 2009; Benz *et al.*, 2012).
Recipient mice were sub-lethally irradiated with a single dose (400cGy) of Cesium irradiation and all transplants were performed by intravenous tail vein injection using a 29.5G insulin syringe. Single HSCs were deposited by FACS into 100µL of medium in a 96-well U-bottom plate. All liquid was subsequently mixed with extra 100µL of PBS and aspirated into the insulin syringe (avoiding air bubbles) and injected into the tail vein. For secondary transplantations, whole bone marrow was obtained from primary recipient by flushing tibiae and femurs with

PBS + 2%FCS. Red cell lysis was performed and an equivalent of one femur (~2x10^7 cells) of each donor mouse was transplanted into at least two secondary recipients.

PB samples were collected in EDTA coated microvette tubes (Sarstedt AGF & Co, Nuembrecht, Germany). Blood was collected from the tail vein at week 8, 12, 16, 20, 24, post-transplantation, unless otherwise stated. Red cell lysis was performed by using NH_4Cl and samples were subsequently analysed for repopulation levels as previously described (Kent et al. 2016; Wilson et al. 2015). Cells were stained for lineage markers using Ly6g BV421 (Clone 1A8), B220 APC (Clone RA3-6B2), CD3e PE (Clone 17A2), CD11b/Mac1 PE-Cy7 or BV605 (Clone M1/70), CD45.1 AF700 (Clone A20), CD45.2 FITC (Clone 104). All antibodies were obtained from Biolegend. Samples were acquired on LSR Fortessa (BD) and flow cytometry data analysing by using FlowJo (Treestar, Ashland, OR, USA).

Single cell RNA sequencing analysis

Single cell RNA sequencing (scRNA seq) analysis was performed as described previously in Picelli et al. 2014 (Smart-seq2). Single ESLAM HSCs were sorted by FACS into 96-well PCR plates containing lysis buffer (0.2% Triton X-100 (Sigma), RNase inhibitor (SUPERase, Thermofisher), nuclease-free water (Thermo Fisher)) Illumina Nextera XT DNA preparation kit was used to prepare the libraries, which were pooled and run on the Illumina Hi-Seq4000 at the CRUK Cambridge Institute Genomics Core. Cells from which low-quality libraries with insufficient sequencing depths were generated were excluded by setting the threshold of number of mapped reads to $>2*10^5$, with mapped reads comprising nuclear genes, mitochondrial genes and ERCCs. A minimum threshold of 20% for reads mapping to known genes was set, in order to exclude empty wells and dead cells. In addition, the threshold for reads mapping to mitochondrial genes was >0.2, to ensure a minimum of 20% of reads to map to non-mitochondrial genes. Protein-coding genes were extracted for further processing. GEO accession number: GSE160131.

Lentiviral transduction of mouse HSCs

7000 ESLAM HSCs cells were isolated and split between 4 wells (1750 cells/well) of a 96-well plate (Corning). Following their isolation, cells were kept in 50 µL of medium (StemSpan, 10%FCS, 20ng/mL IL-11) and were supplemented with polybrene (Sigma) and pHIV-ZsGreen

CSTVR lentivirus supplied by Dr Alasdair Russell from Cancer Research UK (CRUK). Plates were centrifuged at 600g for 30 minutes, at 30°C, to promote infection, before being transferred into a 37°C incubator. Two days after, cells were collected from the wells and resorted for viability (7AAD-). Live cells (4001) were transplanted into 6 sub-lethally irradiated CD45.1 W41 recipient mice (for an approximate dose of 615 cells/mouse) and monitored for donor chimerism as described above, and GFP expression.

Isolation of human CB HSCs and *in vitro* assays

Cord blood samples were obtained from Cambridge Blood and Stem Cell Biobank (CBSB) with informed consent from healthy donors in accordance with regulated procedures approved by the relevant Research and Ethics Committees. Mononuclear cells (MNCs) were isolated using Lymphoprep (Axis Shield PLC, Dundee, UK) or Pancoll lymphocyte separating medium (Pancoll, PAN Biotech, Aidenbach, Germany). Blood was mixed with equal volume of PBS and layered on Lymphoprep/Pancoll. Layered blood was centrifuged at 1400 rpm for 25 min, at room temperature with the brake off. The MNC layer was carefully aspirated and washed with PBS, to remove any separating medium trace. Red cell lysis was subsequently performed by using red cell lysis buffer (Biolegend, San Diego, CA, USA (Biolegend)). MNCs were depleted of differentiated hematopoietic cells by using the human CD34 microbead kit (Miltenyi Biotec, Bergisch Gladbach, Germany) with the following modifications: all cells were resuspended in 90 µL PBS, 2% FCS / 10^8 cells, CD34 Microbeads were used at 30 µL/10^8 cells and FcR Blocking Reagents at 30 µL/10^8 cells. Cells were separated using the AutoMACS cell separation technology (Miltenyi Biotec).

CD34 enriched cells were stained with CD34 APC-Cy7 (Clone HIT2, Biolegend), CD38 PE-Cy7 (Clone HIT2, Biolegend), CD45RA FITC or PE (Clone HI100, Biolegend), CD90 APC or PE (Clone 5E10, Biolegend or Biosciences respectively), CD49f PE-Cy5 (Clone GoH3, Biosciences) and Zombie Aqua (Biolegend) was used as a cell viability marker. HSCs were sorted as $CD34^+$, $CD38^-$, $CD45RA^-$, $CD19^-$, $CD49f^+$, $CD90^+$ on a BD FACS Aria fusion sorter at the NIHR Cambridge BRC Cell Phenotyping Hub facility. The single cells were sorted into individual wells of a 96-well U-bottom plate, each well having been preloaded with 100µL medium.

Liquid cultures and clone size determination of human LT-HSCs

Single HSCs were sorted into 96-well U-bottom plates and cultured in 100µL StemSpan SFEM (STEMCELL) supplemented with 100 units/mL Penicillin and 100µg/mL Streptomycin (Pen/Strep, Sigma-Aldrich), 2mM L-Glutamine (Sigma-Aldrich), 10^{-4}M 2-Mercaptoethanol and 20 ng/mL IL-11 (Biotechne, Abingdon, UK (Bio-techne)), 300ng/mL stem Cell Factor (SCF, R&D)(added when specified), 10% FCS (added when specified). Cell survival was assessed by visual inspection on day 10 (the sorting day is determined as day 0).

Xenotransplantation and Peripheral Blood Analysis

10,862 LT-HSCs were isolated from CD34 enriched CB and cultured into a single well (U-bottom 96-well plate) for 7 days as described above for the single cell culture. On day 7, cell number was assessed by visual inspection and cells were serially diluted in PBS as following: ~110 cells split into 5 recipients (~22 cell per mouse), ~440 cells split into 4 recipients (~110 cells per mouse), ~654 cells split into 3 recipients (~218 cells per mouse). NSG mice were sub-lethally irradiated with a single dose (2.4 Gy) by Cesium irradiation. Twenty-four hours later mice were anesthetised with isoflurane and injected intrafemorally as previously described [29].

PB samples were collected in EDTA coated microvette tubes (Sarstedt AGF & Co, Nuembrecht, Germany). Blood (~100µL) was collected from the tail vein at 8, 12, and 20 weeks post-transplantation. Mice were sacrificed 20 weeks post-transplantation and BM cells were isolated by flushing the injected femur with PBS/FCS. Blood was transferred into polystyrene tubes (Becton Dickinson) tubes and diluted 1:1 with 2%FCS in PBS. 1 mL of Lymphoprep (STEMCELL) was carefully layered at the bottom of the tube and the tubes were centrifuge for 25 min at 500g (brake off). MNCs were collected, washed with PBS and resuspended in 50µL of PBS/FCS and transferred into a 96 u-bottom plate (Falcon) to stain. Cells were stained with the following lineage markers: CD19/FITC (clone HIB19, Biolegend), GlyA/PE (clone HIR2, BD), CD45/PE-Cy5 (clone HI30, Biolegend), CD14/PE-Cy7 (clone M5E2, Biolegend), CD33/APC (clone P67.6, BD), CD19/AF700 (clone HIB19, Biolegend) , CD3/APC-Cy7 (clone HIT3a, Biolegend), CD45/BV510 (clone HI30, Biolegend). Samples were acquired on LSR Fortessa (BD) and flow cytometry data were analysed by using FlowJo v10 (FLOWJO LLC, Ashland, OR, USA). To detect human engraftment, two distinct antibodies against CD45 were used, and cells were considered human if positive for both (CD45^{++}). Mice were considered successfully

repopulated if the percentage of (CD45^{++}) $\geq$ 0.01% (and at least 30 cells were recorded in these gates).

Statistical analysis

Computational analyses were performed in the R programming environment (version 3.6.3). Raw data was processed using the Seurat tool (version 3.2.0)(Butler *et al.*, 2018; Stuart *et al.*, 2019). The recommended standard processing pipeline was applied to perform log-normalisation (default settings) and identify highly variable genes (nfeatures=10,000). Subsequently, expression values were scaled using default parameters. Dimensionality reduction, including principal component analysis (PCA) and Uniform Manifold Approximation and Projection (UMAP) was performed using default Seurat tools. Differential gene expression was performed using negative binomial generalised linear models, as implemented by DESeq2 (version 1.26.0)(Love, Huber and Anders, 2014). Genes with adjusted *p-value* <0.05 and logFC >1.5 were considered significantly differentially expressed (Benjamini-Hochberg corrected). Cell cycle scoring was performed based on average expression of key cell cycle genes, as described previously(Tirosh *et al.*, 2016). Similarly, gene set scoring was computed for previously described HSC proliferation quiescence signatures (Venezia *et al.*, 2004). Such scoring was also applied to gene sets, previously identified as upregulated and downregulated in cells in a G0 state(Cheung and Rando, 2013). Batch effect testing and correction was performed to inform any potential influence of technical bias. Normalisation and variable gene scoring were computed for each batch separately, using variance stabilising transformation. Subsequently, separate batches were integrated using canonical correlation analysis (CCA) by computing integration anchors (parameters: dims = 1:30 and k.filter = 10)(Stuart *et al.*, 2019). A very limited batch correction was identified between Day 1 and Day 2 batches (Supplementary figure 1B). However, full data integration introduced extensive over-correction and downstream analysis was performed without batch correction (data not shown). All data visualisation was computed in R. To inspect downstream IL-11 signalling, the following curated pathways gene sets, as outlined in the gene set enrichment analysis database (Mootha *et al.*, 2003; Subramanian *et al.*, 2005) were retrieved: I) KEGG_JAK_STAT_SIGNALING_PATHWAY (M17411); II) BIOCARTA_NFKB_PATHWAY (M15285); III) HALLMARK_PI3K_AKT_MTOR_SIGNALING (M5923); KEGG_MAPK_SIGNALING_PATHWAY (M10792). Similarly,

KEGG_REGULATION_OF_AUTOPHAGY (M6382) and REACTOME_CELLULAR_SENESCENCE (M27188) were used. All gene sets were subsequently manually curated to exclude ligand and receptor -associated genes (Supplementary Table 1).

To compute gene ontology (GO) and KEGG pathway enrichment, gene symbols were converted to Entrez gene identifiers, using the mouse genome annotation database (org.Mm.eg.db, version 3.10.0). GO terms were extracted from the GO annotation database (GO.db, version 3.10.0). GO term enrichment and KEGG pathways analysis was computed using the Limma package (version 3.42.2). An adjusted *p-value* < 0.05 cutoff was set to determine GO term or KEGG pathway enrichment. Genes identified as significantly differentially expressed between cell types were used conduct pathway enrichment.

Gene set enrichment analysis (GSEA) was performed using the UC San Diego-Broad Institute GSEA software (version 4.0.3) (Mootha *et al.*, 2003; Subramanian *et al.*, 2005). Pre-ranked gene lists were computed based on differentially expressed genes. GSEA was computed using multiple databases, including GO biological processes, KEGG pathways and the Reactome database. Analysis parameter were set as follows: 1000 permutations, weighted enrichment, minimum 15 and maximum 500 genes annotated to gene set.

Supplementary appendix 1: Single-cell time-lapse imaging of single HSCs in hibernation cultures.

Supplementary table 1: JAK/STAT, MAPK, NKFB, PI3K/AKT gene sets

JAK/STAT, MAPK, NFKB, PI3K/AKT gene sets manually curated to exclude ligand- and receptor-associated genes. See also Supplementary Figure 1.

Supplementary Figure 1: Molecular profiling of HSC, hibHSC, HSC+SCF, hibHSC+SCF, related to Figure 5

(A) UMAPs depicting (I) cell type (HSC, blue dots; hibHSC, red dots; HSC+SCF, green dots; hibHSC+SCF, orange dots); (II) batches (batch 0, orange dots; batch 1, blue dots; batch 2, green dots; batch 3, pink dots); days batches were sequenced (day 1, purple dots; day 2, blue dots; day 3, orange dots). (B) MolO gene relative expression in HSC, HSC+SCF, hibHSC, hibHSC+SCF (C) Left panel, PCA of all cells coloured by computationally assigned cell cycle

category, right panel, the 4 cellular states are projected onto the PCA. The PCA was computed using cell cycle genes exclusively.

Supplementary Figure 2: HSC proliferation and quiescence signature genes, related to Figure 5

(A) Violin plots displaying individual proliferation scores by physiological condition (B) Gene Set Enrichment Analysis of the HSC proliferation signature (Venezia *et al.*, 2004), computed using DE genes of direct comparison of HSCs and HSC+SCF. (C) Heatmap of previously identified HSC-specific quiescence signature genes (Venezia *et al.*, 2004), sorted by cell type. (D, E) Gene sets upregulated in G0 cell populations and gene sets downregulated (anti-G0) were used to compute G0 and anti-G0 gene signature scores (Cheung and Rando, 2013). These were projected onto the UMAP depictions (see Figure 5A or Supplementary Figure 1A for reference).

Supplementary Figure 3: Autophagy, senescence, and IL-11RA gene signatures, related to Figure 5

(A) Autophagy gene signature scores projected onto the UMAP landscape and summarised in form of a violin plot. (B) Senescence gene signature depicted as described in (A). (C) Violin plot of the IL-11 receptor gene (IL-11RA1) and gene signature scores for core signalling pathways stimulated by IL-11. Includes: PI3K, NKFB, MAPK and JAK-STAT. (D) Violin plots of top differentially expressed PI3K pathway genes. (E) Top differentially expressed genes of the NF-kB pathway.

Supplementary Figure 4: Specific gene sets are altered during hibernation and SCF-stimulation, related to Figure 6

(A) Violin plots of normalised gene expression of the 13 upregulated genes in SCF-stimulated cells (HSC+SCF, hibHSC+SCF). (B) Volcano plot of differentially expressed genes, comparing HSCs and hibHSC. DE genes are marked in red (logFC>1 and adj *p-value* <0.05, Benjamini-Hochberg corrected). (C) Violin plots of normalised gene expression of genes of interest, downregulated in hibHSC compared to HSC.

Supplementary Figure 5: Genes of interests enriched in hibHSCs, related to Figure 6

UMAPs of selected genes of interests enriched in hibHSC (manually selected from DE gene set. The large majority of the hibHSCs appear in the upper right portion of the plot (see Figure 5A or Supplementary Figure 1A for reference).

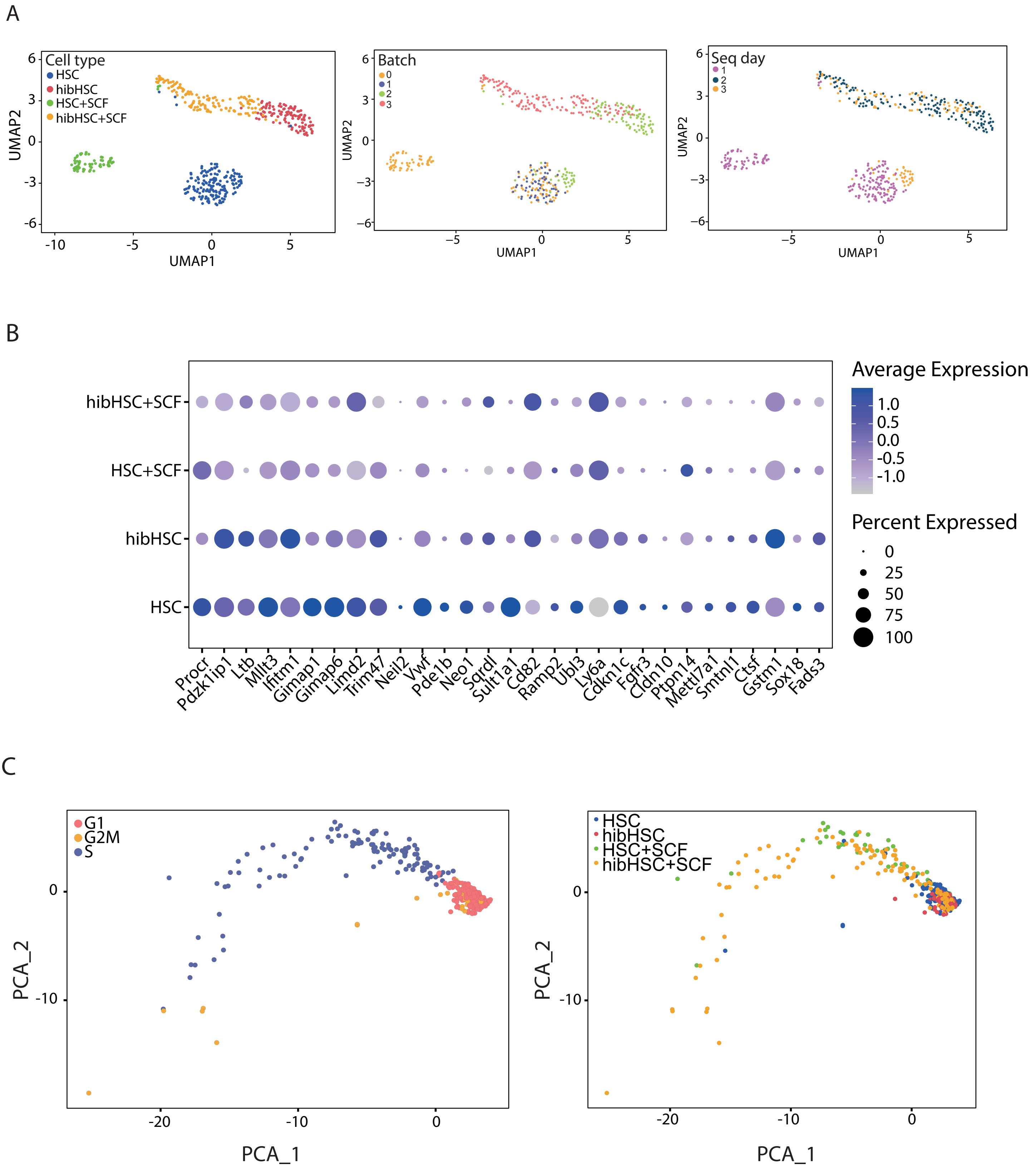
A
Cell type
HSC
hibHSC
HSC+SCF
hibHSC+SCF
UMAP1
UMAP2
Batch
0
1
2
3
Seq day
1
2
3
B
Average Expression
1.0
0.5
0.0
-0.5
-1.0
Percent Expressed
0
25
50
75
100
hibHSC+SCF
HSC+SCF
hibHSC
HSC
Procr
Pdzk1ip1
Ltb
Mllt3
Ifitm1
Gimap1
Gimap6
Limd2
Trim47
Neil2
Vwf
Pde1b
Neo1
Sqrdl
Sult1a1
Cd82
Ramp2
Ubl3
Ly6a
Cdkn1c
Fgfr3
Cldn10
Ptpn14
Mettl7a1
Smtnl1
Ctsf
Gstm1
Sox18
Fads3
C
G1
G2M
S
HSC
hibHSC
HSC+SCF
hibHSC+SCF
PCA_1
PCA_2

S1

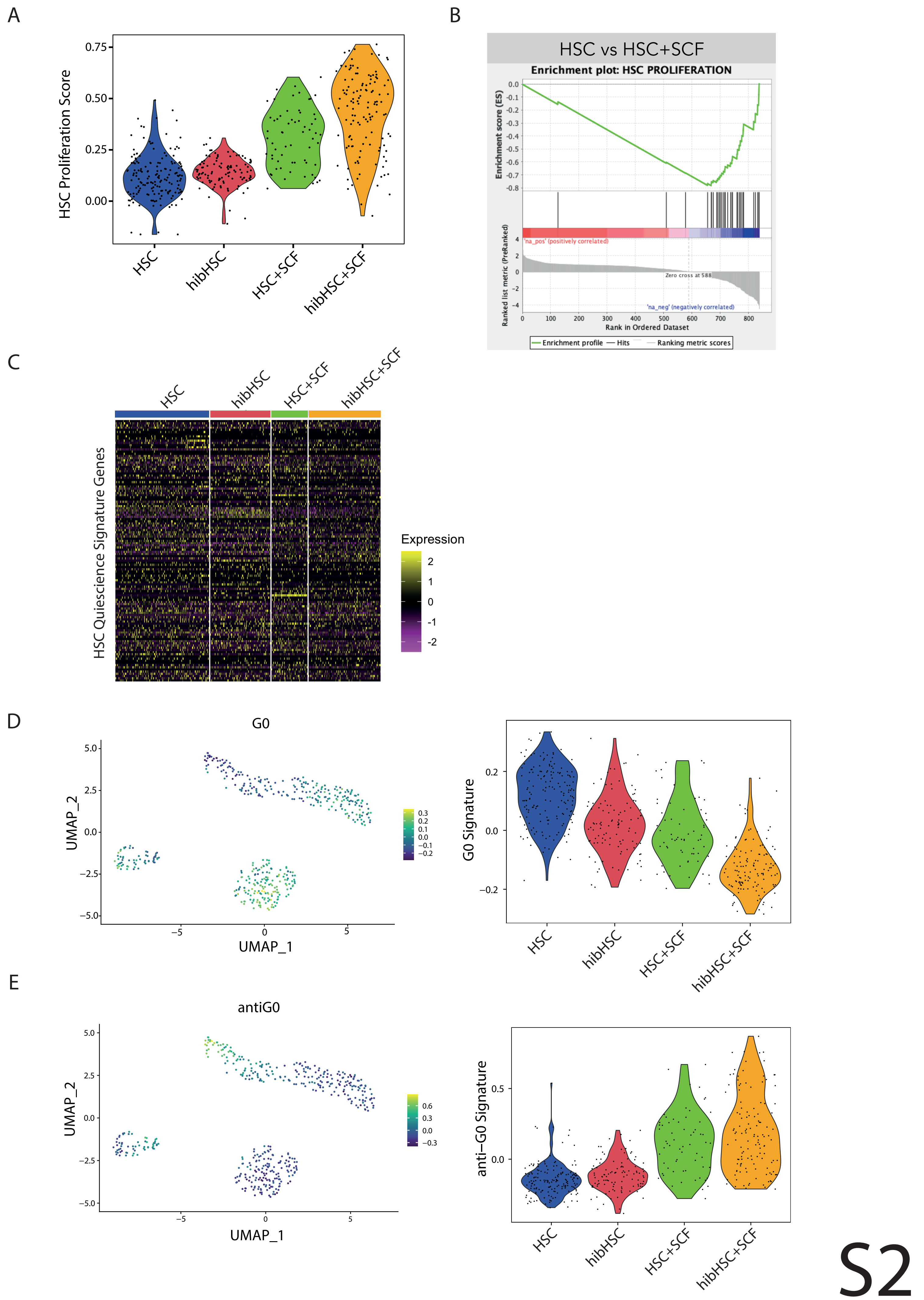
A
B
HSC vs HSC+SCF
Enrichment plot: HSC PROLIFERATION
C
D
E
HSC Proliferation Score
HSC
hibHSC
HSC+SCF
hibHSC+SCF
Enrichment score (ES)
Ranked list metric (PreRanked)
Rank in Ordered Dataset
'na_pos' (positively correlated)
'na_neg' (negatively correlated)
Zero cross at 588
Enrichment profile — Hits — Ranking metric scores
HSC Quiescience Signature Genes
Expression
G0
UMAP_1
UMAP_2
G0 Signature
antiG0
anti−G0 Signature

S2

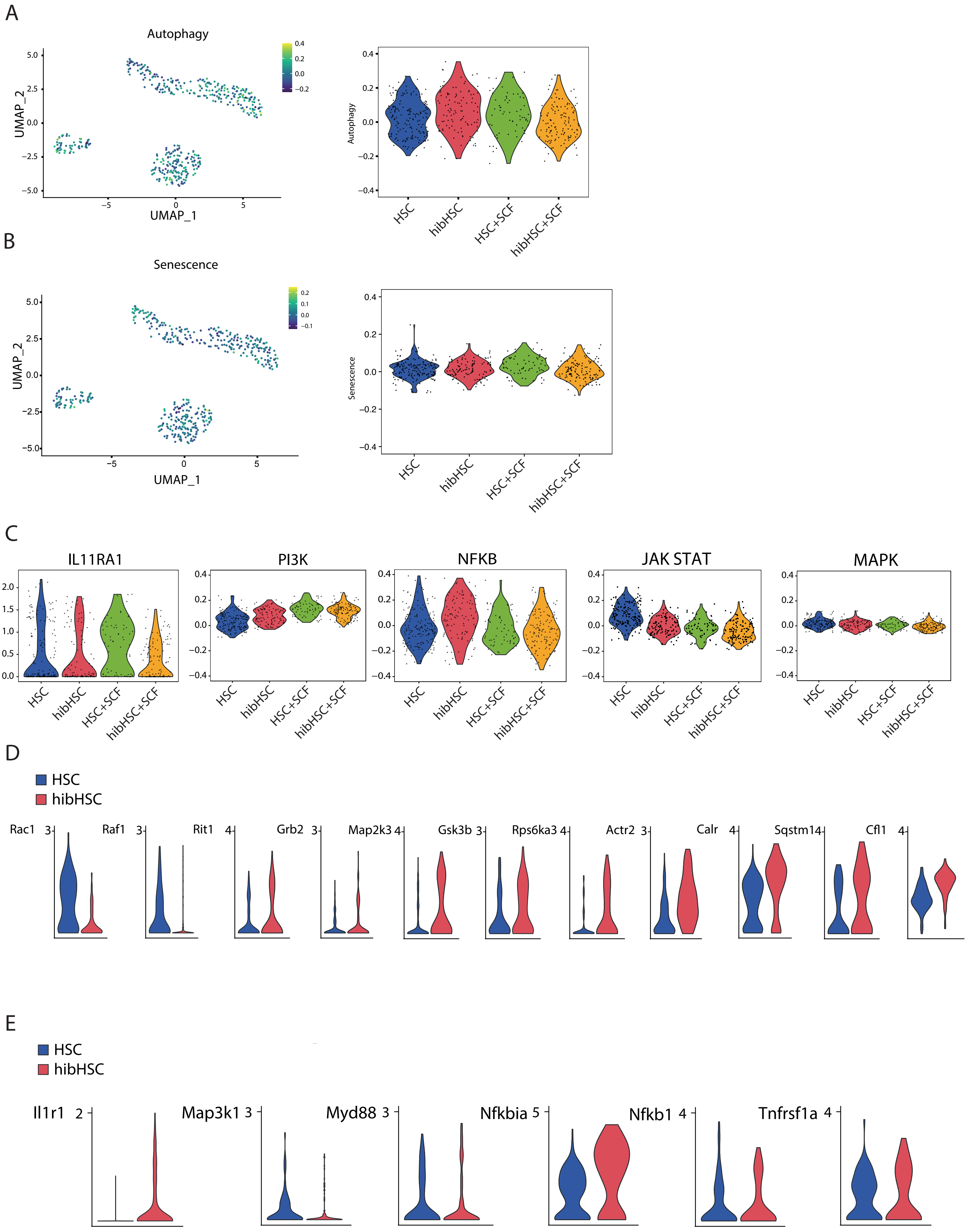
A
Autophagy
UMAP_2
UMAP_1
Autophagy
HSC
hibHSC
HSC+SCF
hibHSC+SCF
B
Senescence
UMAP_2
UMAP_1
Senescence
HSC
hibHSC
HSC+SCF
hibHSC+SCF
C
IL11RA1
PI3K
NFKB
JAK STAT
MAPK
HSC
hibHSC
HSC+SCF
hibHSC+SCF
D
HSC
hibHSC
Rac1
Raf1
Rit1
Grb2
Map2k3
Gsk3b
Rps6ka3
Actr2
Calr
Sqstm1
Cfl1
E
HSC
hibHSC
Il1r1
Map3k1
Myd88
Nfkbia
Nfkb1
Tnfrsf1a

S3

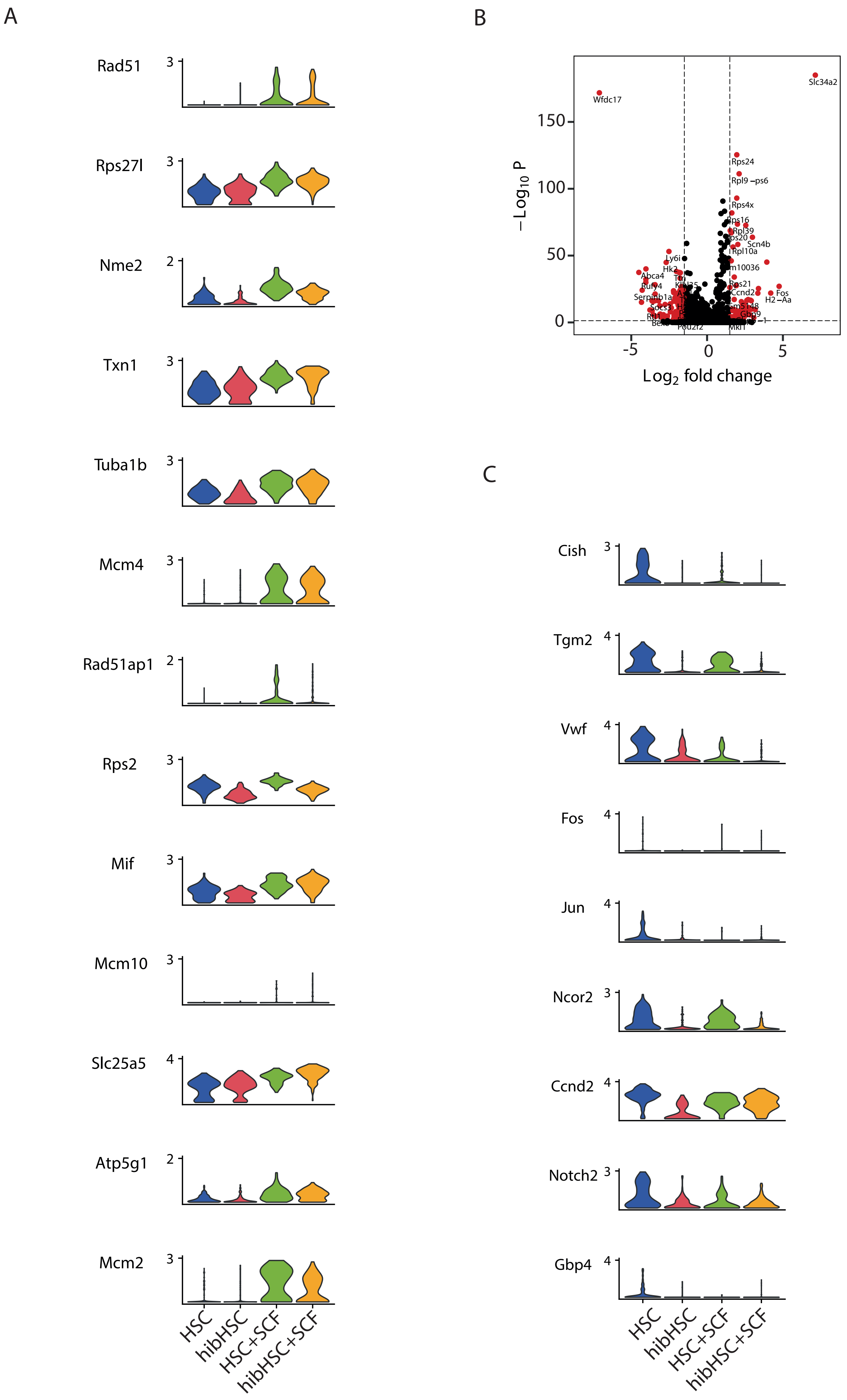
A
B
C
Rad51
Rps27l
Nme2
Txn1
Tuba1b
Mcm4
Rad51ap1
Rps2
Mif
Mcm10
Slc25a5
Atp5g1
Mcm2
HSC
hibHSC
HSC+SCF
hibHSC+SCF
−Log10 P
Log2 fold change
150
100
50
0
-5
0
5
Wfdc17
Slc34a2
Rps24
Rpl9 −ps6
Rps4x
Rpl39
Scn4b
Rpl10a
Ly6i
Hk2
Abca4
Rufy4
Serpinb1a
Socs3
Rps21
Ccnd2
Fos
H2−Aa
Pou2f2
Cish
Tgm2
Vwf
Fos
Jun
Ncor2
Ccnd2
Notch2
Gbp4

S4

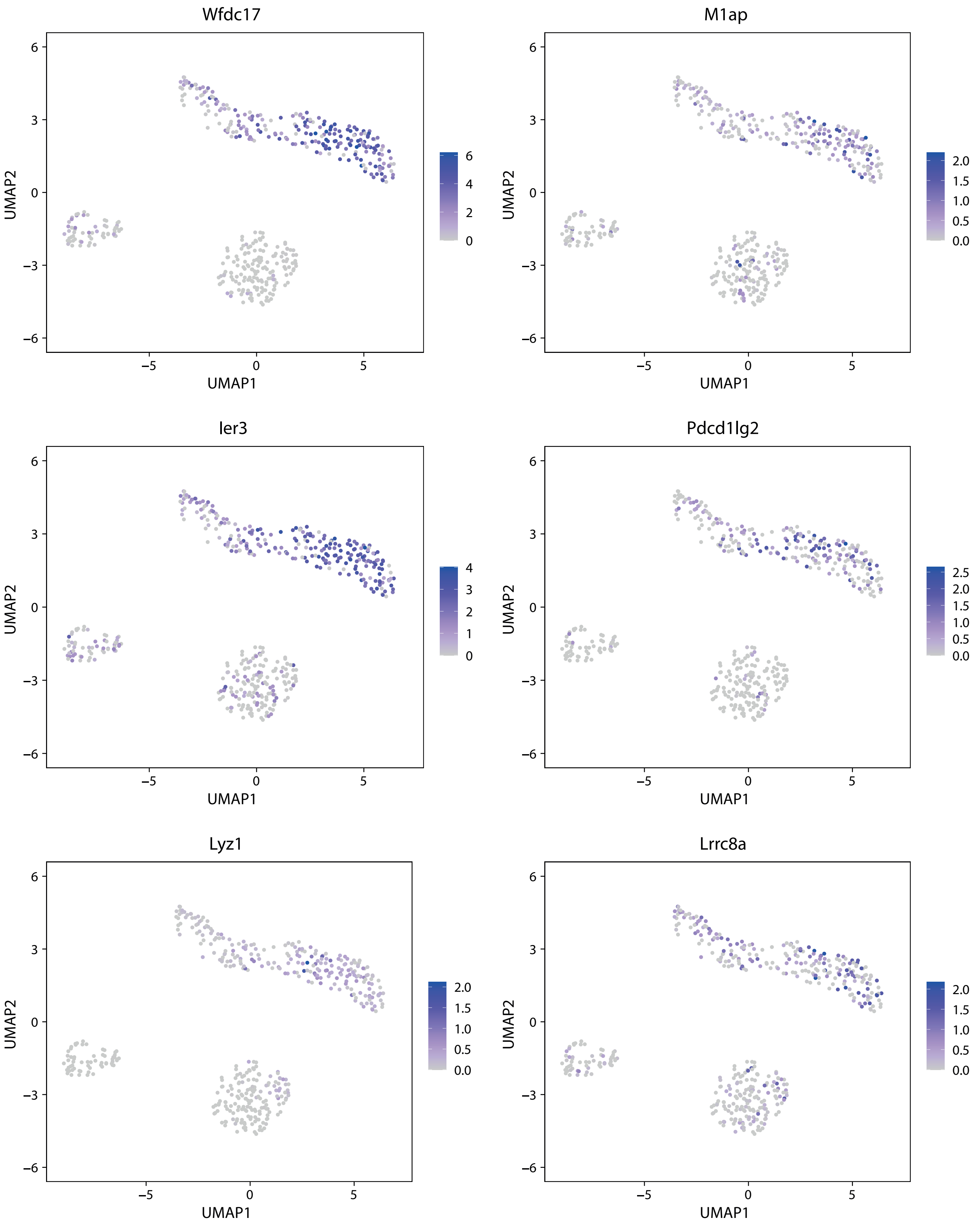
Wfdc17
M1ap
Ier3
Pdcd1lg2
Lyz1
Lrrc8a
UMAP1
UMAP2
6
3
0
−3
−6
−5
0
5
4
2
2.0
1.5
1.0
0.5
0.0
3
1
2.5

S5
